# Direct Aerobic Generation of a Ferric Hydroperoxo Intermediate Via a Preorganized Secondary Coordination Sphere

**DOI:** 10.1021/jacs.1c06911

**Published:** 2021-10-26

**Authors:** Kate A. Jesse, Sophie W. Anferov, Kelsey A. Collins, Juan A. Valdez-Moreira, Maia E. Czaikowski, Alexander S. Filatov, John S. Anderson

**Affiliations:** † Department of Chemistry, The University of Chicago, Chicago, Illinois 60637, United States; ‡ Department of Chemistry, Northwestern University, Evanston, Illinois 60208, United States; § Department of Chemistry, Indiana University, Bloomington, Indiana 47405, United States

## Abstract

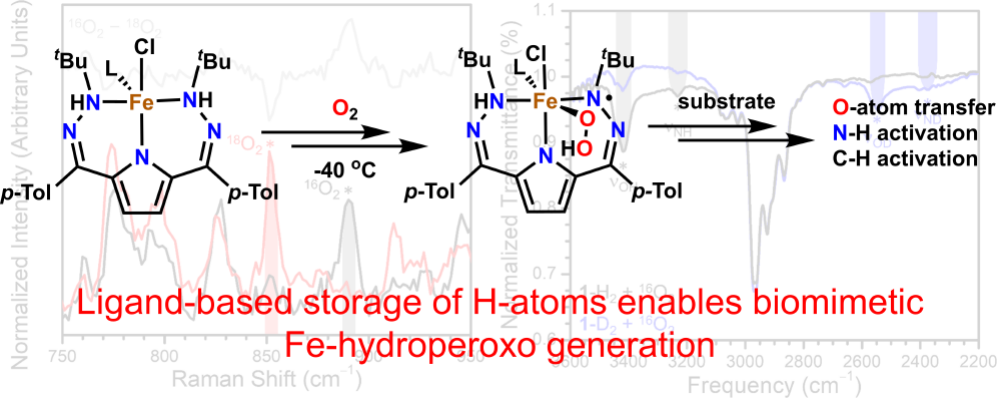

Enzymes exert control over the reactivity of metal centers with precise tuning of the secondary coordination sphere of active sites. One particularly elegant illustration of this principle is in the controlled delivery of proton and electron equivalents in order to activate abundant but kinetically inert oxidants such as O_2_ for oxidative chemistry. Chemists have drawn inspiration from biology in designing molecular systems where the secondary coordination sphere can shuttle protons or electrons to substrates. However, a biomimetic activation of O_2_ requires the transfer of both protons *and* electrons, and molecular systems where ancillary ligands are designed to provide both of these equivalents are comparatively rare. Here, we report the use of a dihydrazonopyrrole (DHP) ligand complexed to Fe to perform exactly such a biomimetic activation of O_2_. In the presence of O_2_, this complex directly generates a high spin Fe(III)-hydroperoxo intermediate which features a DHP^•^ ligand radical via ligand-based transfer of an H atom. This system displays oxidative reactivity and ultimately releases hydrogen peroxide, providing insight on how secondary coordination sphere interactions influence the evolution of oxidizing intermediates in Fe-mediated aerobic oxidations.

## Introduction

Enzymatic systems and, in particular, metalloenzymes mediate a fascinating array of reactions via a carefully evolved secondary coordination sphere. Enzyme active sites leverage effects such as hydrogen bonding, electron transfer pathways, and electrostatic effects to precisely tune the reactivity of metallocofactors.^1,2,11,3−10^ One example illustrating the importance of the secondary coordination sphere is in oxidase chemistry. For example, the terminal oxidant in cytochrome P450 enzymes, Compound I, consists of an Fe(IV)-oxo which is generated from O_2_ via the delivery of proton and electron equivalents from cofactors and the protein superstructure. Other enzymes, such as cytochrome C oxidase, selectively reduce molecular O_2_ to water with the controlled addition of reducing equivalents mediated by an elaborate secondary coordination sphere.^12−16^ While the reactivity and ultimate products of oxidases are varied, the initial steps in O_2_ activation can be quite general, proceeding through initial binding of O_2_ to generate an Fe superoxide intermediate before further activation to an Fe(III)-hydroperoxo intermediate by the addition of a formal H atom from the active site.^12−19^

Molecular chemists have drawn inspiration from these elegant biological examples, and the use of ancillary ligands with designed hydrogen bonding networks,^20−23^ hydrogen shuttling functionalities,^24−30^ or redox reservoirs have emerged as promising strategies in transition metal reactivity and catalysis.^31−40^ While these strategies are effective individually, natural systems are not limited to one interaction type in the secondary coordination sphere but instead leverage all of these effects. For instance, the generation of Fe(III)-hydroperoxo intermediates in the oxidase chemistry discussed above requires the controlled delivery of both protons *and* electrons, or equivalently H-atoms, to O_2_. In contrast, synthetic examples of Fe(III)-hydroperoxo intermediates are almost always generated with exogenous reducing or acid equivalents.^41,42,51,52,43−50^ There are effectively no examples of well-defined Fe(III)-hydroperoxo intermediates generated via biomimetic H-atom transfer from a designed secondary coordination sphere, with one lone example arising from adventitious activation of a supporting ligand.^53^

**Scheme 1:**
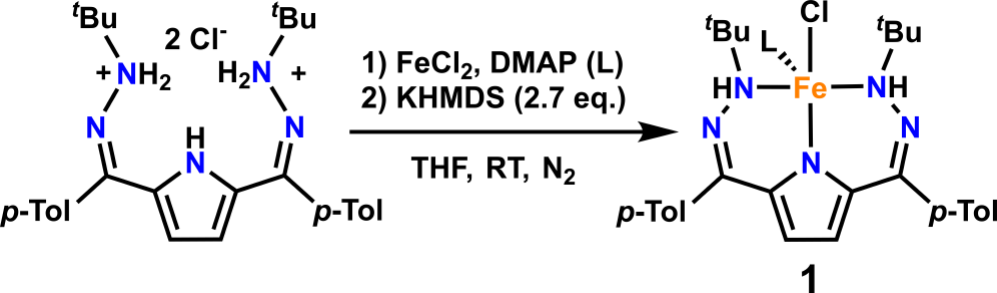
Metalation of ^tBu,Tol^DHP-H_2_·2HCl with FeCl_2_

Synthetic systems where designed ancillary ligands can donate both protons and electrons have been an area of increasing interest.^3,23,62−68,54−61^ However, leveraging this strategy for the biomimetic activation of O_2_ has not been explored. We reasoned that the previously reported pyrrole-based ligand scaffold ^*t*Bu,Tol^DHP (^*t*Bu,Tol^DHP = 2,5-bis((2-*t*-butylhydrazono)(*p*-tolyl)methyl)-pyrrole, Scheme 1), which can donate two electrons and two protons to a substrate, would be an ideal scaffold to demonstrate this principle.^69^ Here, we show that the preorganization of protons and electrons on this ligand scaffold allows for the direct formation of an Fe(III)-hydroperoxo intermediate from O_2_, mirroring the type of reactivity and preorganization found in the protein superstructure of biological systems. This Fe(III)-hydroperoxo intermediate has been characterized by a variety of spectroscopic techniques in addition to kinetic and computational analysis. Reactivity studies show that this system performs both O-atom transfer and H-atom abstraction. Finally, warming of this system results in the formation of a putative dehydrogenated Fe complex along with generation of some H_2_O_2_, providing insight on how the secondary coordination sphere can tune the evolution and terminal products in oxidase-like systems. This investigation demonstrates the utility of redox-active ligands that are also proton responsive for facilitating small molecule activation and aerobic oxidative reactivity.

## Results and Discussion

### Synthesis and Characterization of **1**

The dihydrazonopyrrole ligand, ^*t*Bu,Tol^DHP was isolated as a dication as described previously and metalated via addition to a solution of FeCl_2_ and dimethylaminopyridine (DMAP) in THF, followed by the addition of 2.7 equiv of potassium hexamethyldisilazide (KHMDS).^70^ After workup, an orange powder was isolated in 71% mass yield. Orange crystals suitable for single crystal X-ray diffraction (SXRD) reveal the structure of this orange product to be Fe(^*t*Bu,Tol^DHP-H_2_)(DMAP)Cl (**1**, Scheme 1; see Figure S29). The H’s on the β-N of the hydrazone moieties were found in the difference map and further confirmed by stretches at 3182 and 3170 cm^–1^ via infrared (IR) spectroscopy (see Figure S16). The geometry of **1** is best described as pseudosquare pyramidal with a τ_5_ value of 0.017.^71^ The protonation of the β-N’s results in a twisting of the *t*-butyl groups of the hydrazone arms out of the ligand plane and an overall asymmetric complex in the solid state. This asymmetry in the solid state structure results in one of the β-N H-atoms being positioned close to where a substrate is most likely bind to the Fe-center, thus creating a promising environment for substrate hydrogenation via H-atom abstraction from the ligand.

The Fe–N bond lengths were found to be 2.399(2) and 2.362(2) Å to the hydrazone arms and 2.035(2) Å to the pyrrole N, consistent with a high spin Fe(II) center.^72,73^ This spin and oxidation state for the Fe center was further supported by solution state magnetic measurements, with μ_eff_ = 5.0 μ_B_ by Evans method.^74,75^ Both experiments are consistent with the expected *S* = 2 spin for a high spin Fe(II) complex. EPR spectroscopy in parallel mode on a 15 mM solution in toluene at 15 K shows a broad feature at *g* = 8.4, which is also consistent with an *S* = 2 complex. The paramagnetic nature of **1** was further supported via ^1^H NMR in C_6_D_6_, which features 7 broad features ranging from 29.2 to 3.4 ppm. Nine features in the ^1^H NMR would be expected assuming a symmetric ^*t*Bu,Tol^DHP-H_2_ ligand in solution. This suggests that some features, likely those with higher proximity to the high spin Fe(II) center, have been broadened or shifted to such an extent that they are no longer visible. It further suggests that the hydrazone arms, which are inequivalent in the solid-state, equilibrate in solution at room temperature.

### Aerobic Reactivity

**Figure 1:**
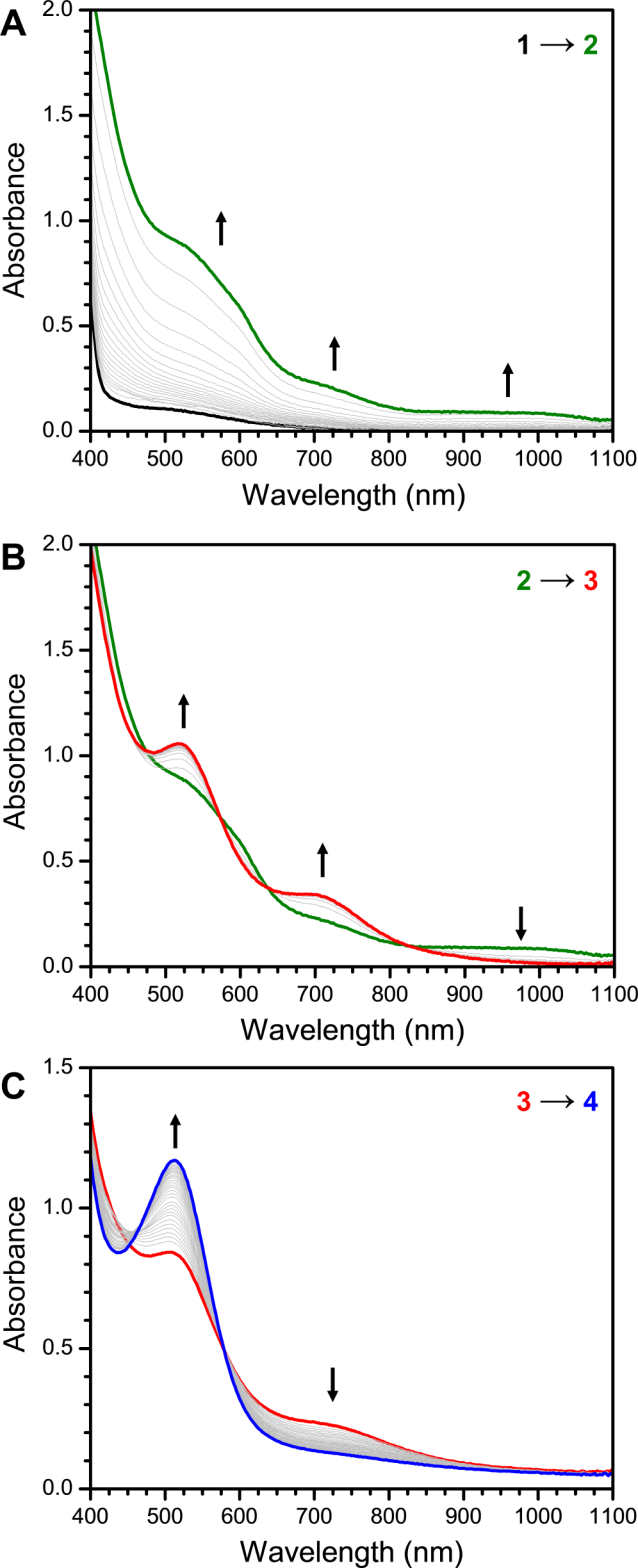
UV–visible spectroscopy of a 0.35 mM solution of **1** in toluene at −60 °C upon addition of 0.5 mL of O_2_ where **1** is black, **2** is green, **3** is red, and **4** is blue. (A) Formation of **2** with scans every 1 min starting 2 min after O_2_ addition. (B) Formation of **3** from **2** with scans every 5 min. (C) Growth of **4** from **3** at −10 °C from a 0.35 mM solution of **1** in toluene. Scans were collected every 10 min.

The reaction of molecular O_2_ with **1** in toluene was monitored at low temperature by UV–visible spectroscopy (Figure 1). The spectrum of complex **1** is largely featureless at wavelengths longer than 400 nm. Upon addition of excess O_2_ to a 0.35 mM solution in toluene at −60 °C, broad features grow in throughout the spectrum assigned as a new intermediate or mixture, **2** (Figure 1A). After 20 min, further evolution to a new species, **3**, is observed (Figure 1B). The conversion between **1** to **2** and eventually **3** is convoluted, potentially due to the short lifetime of **2** even at temperatures as low as −80 °C. As such, only the feature at 996 nm can be concretely assigned to **2**. Conversely, intermediate **3** is stable once formed under these conditions and persists without noticeable decomposition at temperatures up to −40 °C, with features at 528 and 716 nm. Further evolution of the spectrum is observed above these temperatures to form **4** (Figure 1). The relative stability of **3** led us to investigate its assignment more thoroughly.

### Characterization of Intermediate **3**

Kinetic studies were conducted to better understand the transformations observed by UV–visible spectroscopy. Kinetic analysis of the transformation from **1** to **2** was conducted under pseudo first-order conditions by monitoring the growth and disappearance of the peak in the UV–visible spectrum at 996 nm to avoid overlap with features from **3**. The transformation from **1** to **2** did not fit standard zero-, first-, or second-order kinetics well. This suggests a complicated pre-equilibrium or that **2** is a mixture of products. Consistent with this hypothesis, Mössbauer spectra of a frozen solution of **2** show a complicated mixture of species, likely due to the transient nature of **2**, its speciation, or other intermediates or byproducts present in the solution (see Figure S26). In contrast, the transformation from the major species in **2** to complex **3** was found to follow first-order kinetics in Fe using an exponential fit of the data (see Tables S2–S8). Eyring analysis of the transformation from **2** to **3** gives an Δ*H*^‡^ = 7.6(1.0) kcal/mol and Δ*S*^‡^ = −34(4.9) cal/(mol K)^−1^. We also monitored the reaction of O_2_ with N–D deuterated complex **1** to examine the KIE for the formation of **3** (see Figure S30, Tables S9–S11). While the error in the rates is sufficiently large to preclude any concrete conclusions, the data does reveal a small KIE of around 1. This suggests that the rate determining step for the formation of **3** does not involve H-atom or proton transfer, as might be expected for a rate determining ligand association or dissociation. The significant and negative entropy of activation for this conversion is also consistent with a rate determining ligand association, but this interpretation is tentative without more detailed kinetic analysis.

**Figure 2:**
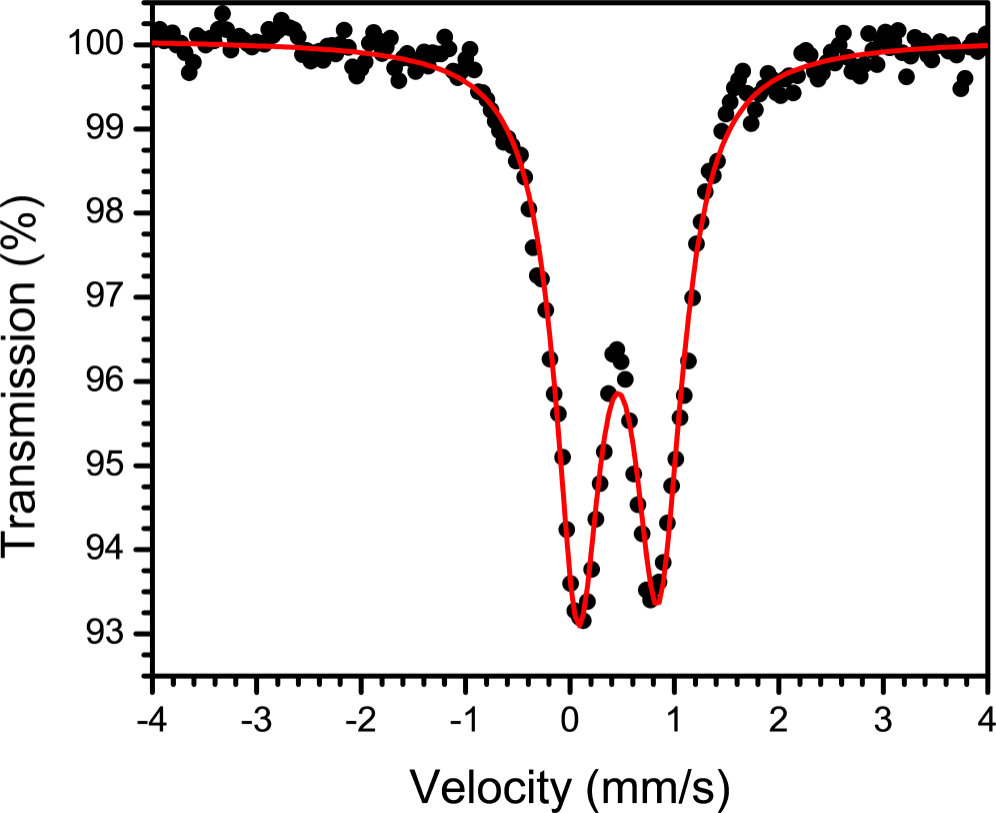
Mössbauer spectra of **3** at 80 K. Simulation in red with δ = 0.46(1) mm/s and Δ*E*q = 0.76(1) mm/s.

The first-order kinetics in the conversion of **2** to **3** in the UV–visible spectra suggest that the nuclearity of the Fe complexes is maintained in this transformation. This observation suggests that if **2** were mononuclear, bimolecular pathways could be ruled out. To test this hypothesis, reactions at −40 °C with 0.5 equiv and 1 equiv of O_2_ were monitored by UV–visible spectroscopy and then compared to the spectrum of **3** formed in the presence of excess O_2_ (see Figure S12). These data show that at least 1 equiv of O_2_ per Fe must be added to the reaction to generate the same intensity of signals as that observed with excess O_2_. When only 0.5 of an equivalent of O_2_ was used, the absorbances for **3** only reached ∼50% of the intensity seen in the presence of excess O_2_. These observations argue against the formation of dimeric complexes, such as bridging peroxo species, and furthermore suggest that **3** is a mononuclear Fe complex.

Given the stability of **3** below −40 °C, we were able to characterize this complex by a variety of spectroscopic techniques. The Mössbauer spectrum of **3** (Figure 2) shows that this complex is formed cleanly and has an isomer shift of 0.46(1) mm/s, consistent with a high-spin Fe(III) center. The relatively small quadrupole splitting of 0.76(1) mm/s is similarly consistent with a high-spin Fe(III) center. X-ray absorption spectroscopy collected on **1** and **3** shows an increase in the K-edge energy consistent with an increase in the oxidation state from an Fe(II) complex to an Fe(III) complex (see Figure S28). Numerous unsuccessful attempts were made to grow crystals suitable for X-ray diffraction at −78 °C, and the EXAFS region of **3** is unfortunately too noisy for reliable structural determination.

**Scheme 2:**
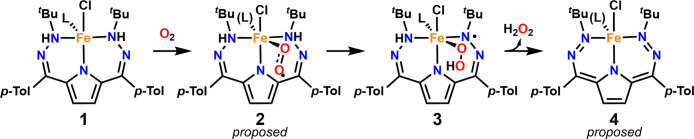
Reaction of **1** with O_2_ Note: **2** and **4** are only proposed intermediates and are only present as mixtures.

Two likely high-spin Fe(III) products of the reaction of **1** with O_2_ are an Fe(III)-superoxo complex featuring a ^*t*Bu,Tol^DHP-H_2_ ligand or an Fe(III)-hydroperoxo complex, where an H-atom has been abstracted by a putative superoxo precursor to form the ^*t*Bu,Tol^DHP-H^•^ ligand radical and a hydroperoxo ligand; these structures are shown in Scheme 2. Both complexes would be expected to have an overall spin of *S* = 2 assuming an *S* = 5/2 Fe(III) center coupled antiferromagnetically to a ligand based radical of the superoxo ligand or the ^*t*Bu,Tol^DHP-H^•^ ligand, respectively. EPR spectroscopy in parallel mode of **3** as a 15 mM frozen solution at 15 K in toluene was collected to test for this hypothesized spin-state. The parallel mode X-band EPR spectrum of **3** shows a feature at *g* = 10.6 consistent with an *S* = 2 species, as would be expected for either of these assignments (see Figure S23).

**Figure 3:**
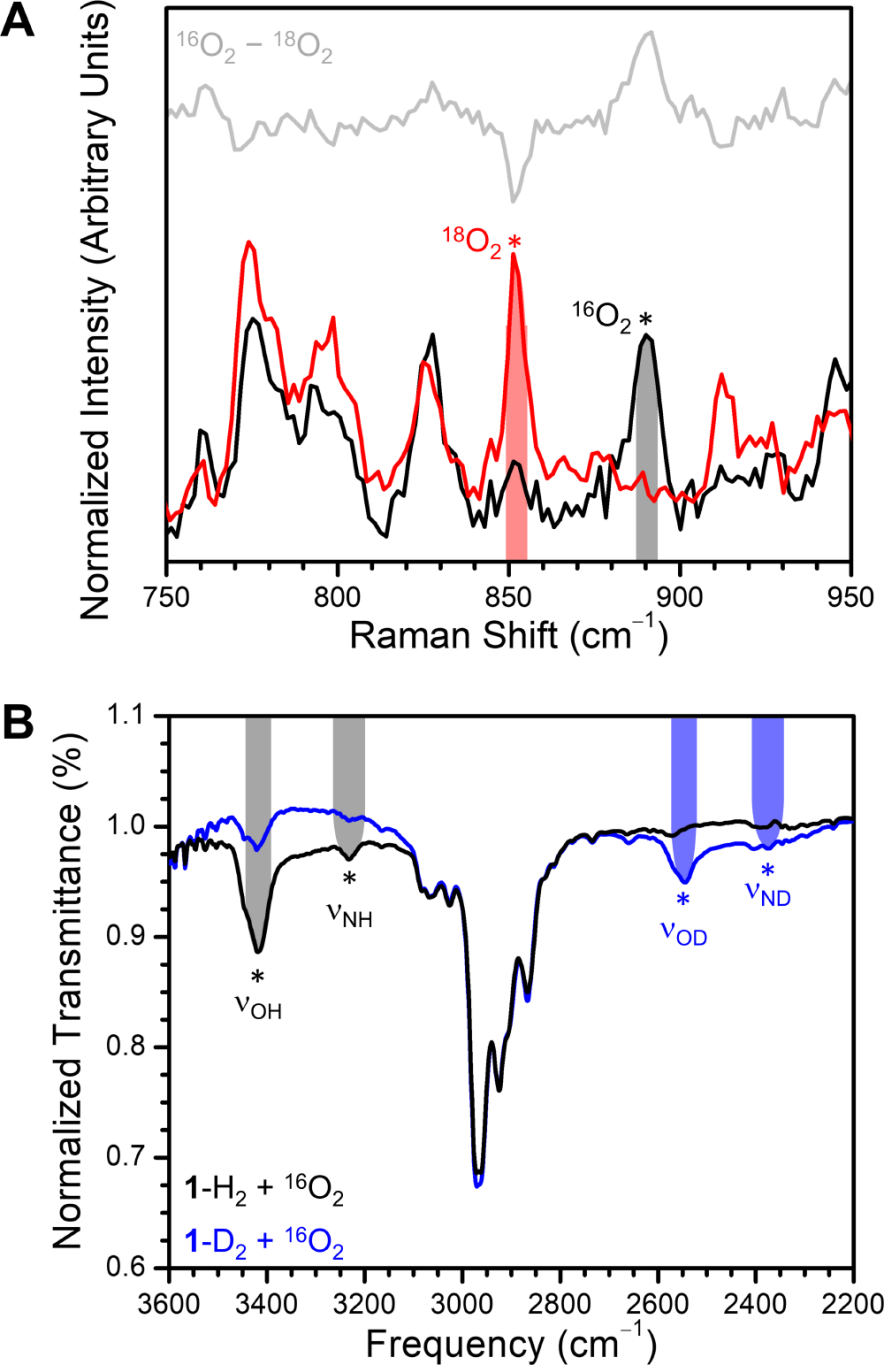
(A) Variable isotope Raman spectra of the reaction of **1** in the solid state with O_2_ at −40 °C to form **3**. The reaction with ^16^O_2_ vs ^18^O_2_ are shown in the peroxo stretching region.The difference spectrum is shown above the normalized data for ^16^O_2_ and ^18^O_2_. (B) Variable isotope IR spectra of the reaction of **1** with O_2_ at RT to form **3**. Proteo- vs deutero-**1** (83% enriched) reacted with ^16^O_2_ collected as concentrated solutions in chlorobenzene.

Vibrational spectroscopy was then used to look for the putative O–O, O–H, and N–H stretches for an Fe(III)-superoxo complex or an Fe(III)-hydroperoxo complex using isotopic labeling studies. Raman spectroscopy was performed on **3** generated from **1** in the solid state at −44 °C and collected at 77 K. Data were collected with a 532 nm laser on 10% power (Figure 3A; see SI experimental). These data show a peak at 890 cm^–1^ in the spectrum collected with ^16^O_2_ that shifts to 850 cm^–1^ in the spectrum collected with ^18^O_2_. This shift is similar to the 50 cm^–1^ shift that would be expected for an O–O stretching frequency assuming a simple harmonic oscillator. The slight deviation from theory may arise from overlapping vibrations as well as coupling with other vibrational modes.

In addition to Raman data, similar features are seen by IR spectroscopy. Intermediate **3** was generated as a solution in chlorobenzene, as a thin film on KBr plates, and as a solid in a KBr matrix. Although these IR measurements were conducted at RT rather than below −40 °C, a comparison of the ^16^O_2_ and ^18^O_2_ spectra across all these techniques reveals weak and broad signals which are consistent with those seen by Raman spectroscopy. There is a disappearance of a feature at ∼885 cm^–1^ and the growth of a new feature at ∼845 cm^–1^ in the ^16^O versus the ^18^O data (see Figures S17–S22).

These vibrations in both the Raman and IR data more closely align with an O–O stretch for an Fe(III)-hydroperoxo assignment rather than with an Fe(III)-superoxo complex. A superoxo complex would be expected to have an O–O stretch between 1000 and 1300 cm^–1^.^76^ The observed O–O stretching frequency compares favorably with related Fe-hydroperoxo complexes.^41,42,77,43−50^

Additionally, two features are seen by IR spectroscopy at 3420 and 3230 cm^–1^, which could be assigned either as two N–H stretches or as an N–H and O–H stretch for an Fe(III)-superoxo and Fe(III)-hydroperoxo, respectively (Figure 3B). Given the much higher intensity of the feature at 3420 cm^–1^ relative to the feature at 3230 cm^–1^, and the dramatically higher stretching frequency for one of these features versus ν_NH_ in **1**, the most reasonable conclusion is that these stretches do not both arise from N–H moieties but rather are an N–H and an O–H stretch, consistent with an Fe(III)-hydroperoxo complex. IR spectra of this mixture were collected using a deuterated version of **1**, where the metalation was completed using 81% enriched ^*t*Bu,Tol^DHP-D_2_·2DCl ligand salt. When reacted with O_2_, IR spectroscopy of this reaction using the deuterated version of **1** shows growth of a feature at 2546 cm^–1^, which is consistent with an O–H to O–D shift. Similarly, the feature at 3230 cm^–1^ largely disappears when **1** is enriched with deuterium and a broad feature grows in at 2375–2404 cm^–1^. Both shifts are consistent with the expected shift assuming a perfect harmonic oscillator model. Together, these vibrational data are most consistent with an Fe(III)-hydroperoxo complex rather than an Fe(III)-superoxo complex.

### Computational Analysis of **3**

**Figure 4:**
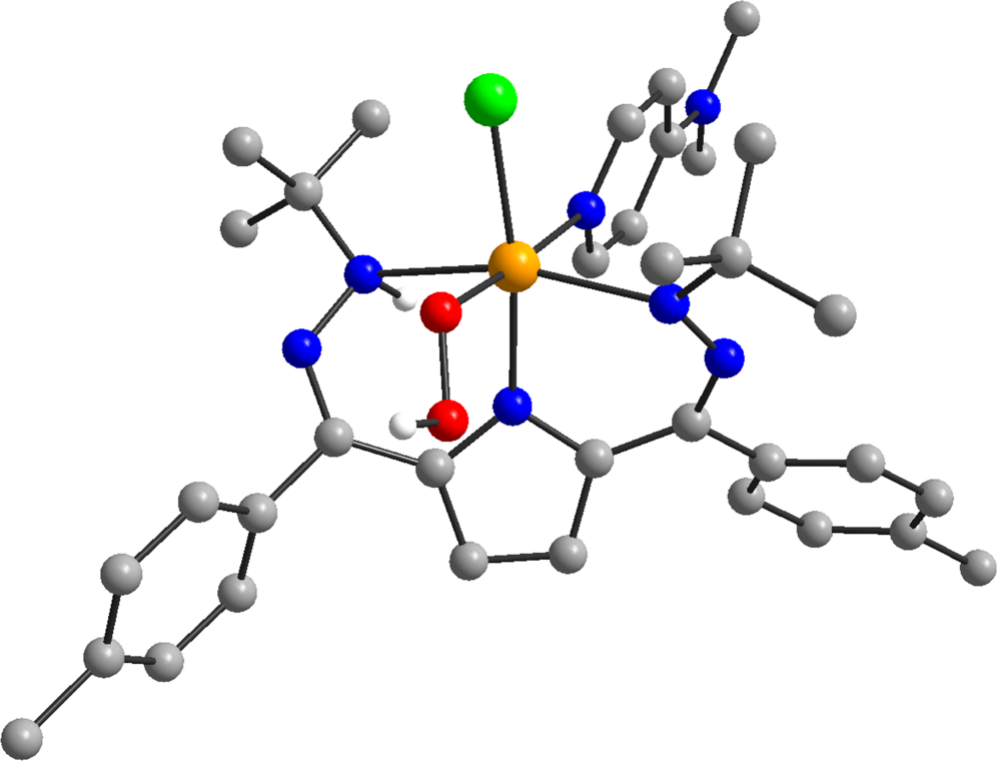
DFT computed structure of **3** with Fe in orange, C in gray, N in blue, O in red, Cl in green, and selected H’s in white.

All of the experimental data on **3** are consistent with the assignment of an Fe(III)-hydroperoxo complex generated from intramolecular activation of O_2_. However, in the absence of direct structural data, we wanted to additionally support this assignment with a computational treatment. The proposed assignment of **3** was therefore investigated using density functional theory (DFT) calculations. Geometry optimizations and frequency calculations for both the putative Fe(III)-superoxo and Fe(III)-hydroperoxo complexes were done using the B3P hybrid functional.^78−80^ The predicted structure of **3** is shown in Figure 4 and features bond lengths of 1.89 and 1.41 Å for the Fe–O and O–O bonds, respectively, as expected for an Fe(III)-hydroperoxo complex. The computed spin density is also consistent with a high-spin Fe(III) center antiferromagnetically coupled to a ligand radical (see Figure S34). Finally, the DFT-predicted vibrational frequencies for **3** also agree well with those observed experimentally (see Table S15).

A single point calculation was then run using the optimized geometries to predict the Mössbauer isomer shift and quadrupole splitting parameters using the TPSSh functional, with a basis set of CP(PPP) on Fe, and an increased polarization on all other atoms except H.^78−81^ These calculations suggest that an Fe(III)-hydroperoxo is the best assignment for the Mössbauer data. The theoretical isomer shift of 0.47 mm/s and quadrupole splitting of −0.85 mm/s are in good agreement with the experimentally determined values of 0.46(1) mm/s and 0.76(1) mm/s. Conversely, the values predicted for an Fe(III)-superoxo assignment match much more poorly, with a preicted isomer shift of 0.61 mm/s and quadrupole splitting of 1.07 mm/s.

Time dependent DFT (TD-DFT) was also run to calculate the theoretical UV–visible spectrum of the proposed Fe(III)-superoxo and Fe(III)-hydroperoxo complexes using the PBE0 functional on the previously optimized geometries.^78−80,82^ The theoretical spectrum for a 6-coordinate Fe(III)-hydroperoxo complex with a ligand based radical was found to be a good fit for the experimental spectrum while the Fe(III)-superoxo complex was not (see Figure S35), further suggesting that the correct assignment for **3** is that of an Fe(III)-hydroperoxo with a ligand based radical on ^*t*Bu,Tol^DHP-H^•^. In addition to this computational match, we note that the experimental UV–visible data for **3** is consistent with literature UV–visible data where a strong feature grows in at ∼500 nm upon the formation of an Fe(III)-hydroperoxo, which arises from ligand-to-metal charge transfer (LMCT) from the hydroperoxo ligand to the Fe-center.^41−45,50,53,83,84^ TD-DFT of the Fe(III)-hydroperoxo with a ligand based radical is also consistent with LMCT transitions contributing to the peak at 518 nm (see Figure S36). Furthermore, TD-DFT suggests that the second feature in these data at 714 nm is likely due to a MLCT between the Fe-center and the ligand based radical of the ^*t*Bu,Tol^DHP-H^•^ ligand (see Figure S36). This presence of a low energy absorbance was previously seen with isolated ^*t*Bu/Ph,Tol^DHP ligand radicals on Ni and Fe centers, further supporting the assignment of **3** with a ligand based radical on ^*t*Bu,Tol^DHP-H^•^ (Scheme 2).^53,69,85^

### Evolution of **3** Upon Warming

As mentioned above, upon being warmed, **3** reacts further to form a new complex, **4**, by UV–visible spectroscopy (Figure 1). Unfortunately, **4** is not stable at any temperature that it can be produced at and will slowly bleach over time, making characterization challenging (see Figure S7). One possible assignment for **4** is that of an Fe(IV)-oxo which forms by abstracting a second H-atom from the ^*t*Bu,Tol^DHP-H^•^ ligand and releasing water. This would result in an oxidized ^*t*Bu,Tol^DHP ligand in addition to a highly oxidized Fe(IV) center, mimicking the reactivity of cytochrome P450. Another possibility is that when the second H-atom is abstracted from the ligand, H_2_O_2_ is released and an Fe(II) complex featuring an oxidized ^*t*Bu,Tol^DHP ligand is formed. Allowing **3** to warm such that **4** could evolve allowed us to collect a Mössbauer spectra of a mixture of **3** and **4**. This shows an isomer shift of 0.38(1) mm/s and a quadrupole splitting of 1.32(2) mm/s for **4**, which best aligns with a low spin Fe(II) center or an Fe(III) center (see Figure S27).^86^ This observation argues against an Fe(IV) assignment and suggests that loss of H_2_O_2_ to generate a Fe(II) center with an oxidized ligand is a more reasonable pathway. To test the feasibility of an Fe(II) product with an oxidized DHP ligand, TEMPO^•^ was added to solution of **1** to putatively remove two H-atoms. When this experiment is carried out, UV–visible absorbances matching those seen for **4** grow in, suggesting that the tentative assignment of **4** as Fe(^*t*Bu,Tol^DHP)(DMAP)Cl is reasonable (see Figure S13). While circumstantial, these data suggest that the net reactivity of this system may be to release H_2_O_2_ when reacted with molecular oxygen (Scheme 2).

To determine if H_2_O_2_ was formed from the reaction of **1** with O_2_, a chemical test was conducted. It has been reported that H_2_O_2_ reacts selectively and stoichiometrically with 1,3-diphenylisobenzofuran (DPBF) to form 9-hydroxyanthracen-10(9H)-one. When DPBF is reacted with other peroxides, 1,2-dibenzoylbenzene will form instead.^87^ As such, this is an excellent method for probing if H_2_O_2_ specifically is produced as a byproduct. The reaction of **1** with O_2_ was conducted, the solution was sparged with N_2_, then 10.2 equiv of DPBF were added. A small amount of 9-hydroxyanthracen-10(9H)-one was reproducibly observed by gas chromatography–mass spectrometry (GC-MS, see Figure S42), as would be expected if H_2_O_2_ had been produced. While this test is indirect, the selective formation of 9-hydroxyanthracen-10(9H)-one is strong evidence for the presence of H_2_O_2_. This result is also consistent with decomposition of **3** by loss of H_2_O_2_ to generate **4**, as depicted in Scheme 2. It is noteworthy that this system appears to generate H_2_O_2_ instead of an Fe(IV) oxo intermediate and water. This mechanistic bifurcation is tightly regulated in biological systems, and we suspect that the selectivity for H_2_O_2_ formation in this synthetic system is the result of the orientation of the hydrogens in the secondary coordination sphere. Related H-bonding effects have been noted recently in Cu systems.^18,19,88^

### Oxidative Reactivity

Encouraged by the biomimetic nature of this system, oxidative reactivity in the presence of molecular oxygen was investigated. By low temperature UV–visible spectroscopy, **3** was found to react with 10 equiv of PPh_3_ slowly at −40 °C. We speculate that **3** may perform O-atom transfer to PPh_3_ and form OPPh_3_, presumably via nucleophilic attack by the phosphine onto the electrophilic hydroperoxocomplex. When this reaction was conducted in a J-Young tube and monitored by ^31^P NMR at room temperature, 1 equiv of OPPh_3_ was observed (see Figure S5). When the reaction was done in the presence of ^18^O_2_, enrichment of the isotopically labeled OPPh_3_ product was observed by GC-MS analysis demonstrating that the source of the oxidizing equivalent is the added O_2_ (see Figure S37). The formation of only 1 equiv of OPPh_3_ per equivalent of **1** is significant. This means that only one of the O-equivalents from O_2_ is ultimately being accessed for oxidative reactivity, consistent with the proposed steps shown in Scheme 2.

The reactivity of **3** and **4** toward H-atom abstraction was also investigated with diphenylhydrazine (DPH), dihydroanthracene (DHA), and 1,4-cyclohexadiene (CHD). Complex **3** was found to react with DPH at −40 °C by UV–visible spectroscopy, but not DHA or CHD, suggesting that **3** can abstract H-atoms from a relatively weak N–H bond but not stronger C–H bonds at low temperature. Conversely, experiments conducted at room temperature show both C–H activation and oxygenation products for DHA, toluene, and PPh_3_ when analyzed by GC-MS (see Figures S39–41). Overall, the observed reactivity is consistent with oxidation occurring from either **3**, from an oxidizing byproduct such as hydroxyl radical or H_2_O_2_ as shown in Scheme 2, or as some Fe-activated H_2_O_2_ complex (i.e., an Fe-oxo complex) at temperatures above −40 °C. The convoluted nature of this reaction mixture makes it difficult to discern the exact nature of the active oxidant at these higher temperatures where oxygenated products are observed.

## Conclusions

In this study, we have directly synthesized an Fe(III)-hydroperoxo intermediate from an Fe(II) complex featuring pendant H-atom equivalents and O_2_. This unusual Fe(III)-hydroperoxo complex with a ^*t*Bu,Tol^DHP-H^•^ ligand based radical was characterized by a variety of spectroscopic and computational techniques in addition to kinetic studies. This intermediate is thermally unstable, and we propose decomposition to a terminal Fe(II) product with release of H_2_O_2_. This system also displays oxidative reactivity toward a variety of substrates. This reactivity can stem either directly from the hydroperoxo intermediate or potentially from the H_2_O_2_ byproduct. This study demonstrates that the combination of redox-active ligands and pendant H donors allows for the unimolecular activation of O_2_ via controlled movement of protons and electrons from the secondary coordination sphere. Furthermore, this reactivity is reminiscent of biology’s strategy of using redox-active cofactors, Fe centers, and pendant protons shuttled from nearby amino acids or active site water molecules in enzymes to mediate challenging reactivity with O_2_.

## Experimental Section

### General Methods

All chemicals were purchased from commercial suppliers and used without further purification. All manipulations were carried out under an atmosphere of N_2_ using standard Schlenk and glovebox techniques. Glassware was dried at 180 °C for a minimum of 2 h and cooled under a vacuum prior to use. Solvents were dried on a solvent purification system from Pure Process Technologies and stored over 4 Å molecular sieves under N_2_. Tetrahydrofuran (THF) was stirred over NaK alloy and run through an additional alumina column prior to use to ensure dryness. Solvents were tested for H_2_O and O_2_ using a standard solution of sodium-benzophenone ketyl radical anion. CD_3_CN, C_6_D_6_, and *d*_8_-toluene were dried over 4 Å molecular sieves under N_2_.

^1^H and ^31^P{^1^H} NMR spectra were recorded on Bruker DRX 400 or 500 spectrometers. Chemical shifts are reported in ppm units referenced to residual solvent resonances for ^1^H and ^31^H{^1^H} spectra. UV–vis spectra were recorded on a Bruker Evolution 300 spectrometer and analyzed using VisionPro software. IR spectra were obtained on a Bruker Tensor II spectrometer with the OPUS software suite. All IR samples were prepared nujol mulls or collected between KBr plates. EPR spectra were recorded on an Elexsys E500 Spectrometer with an Oxford ESR 900 X-band cryostat and a Bruker Cold-Edge Stinger. EPR data was analyzed using SpinCount. Single crystal X-ray diffraction data were collected in-house using Bruker D8 Venture diffractometer equipped with Mo microfocus X-ray tube (λ = 0.71073 Å).

X-ray near-edge absorption spectra (XANES) were employed to probe the local environment of Fe. Powder samples were prepared by material grinding finely. A Teflon window was sealed on one side with Kapton tape, and powder was then transferred to the inside of this ring before it was compacted with a Teflon rod and the remaining face sealed with Kapton tape. After transfer of the material, the window was sealed with Kapton tape. All sample preparation was performed under an inert atmosphere. Frozen solution samples were prepared by making a concentrated solution in THF of the starting material, removing the sample from the glovebox, cooling the sample in a bath, then reacting the sample with O_2_ by syringing the gas into the sample and bubbling it through. After being allowed to react, the sample was exposed to air and a precooled pipet was used to transfer the solution to a Teflon window lined on one side with Kapton tape. The solution was frozen using liquid nitrogen and then stored in liquid nitrogen until collection. Data were acquired at the Advanced Photon Source at Argonne National Laboratories with a bending magnet source with ring energy at 7.00 GeV. Fe K-edge data were acquired at the MRCAT 10-BM beamline. The incident, transmitted and reference X-ray intensities were monitored using gas ionization chambers. A metallic iron foil standard was used as a reference for energy calibration and was measured simultaneously with experimental samples. X-ray absorption spectra were collected at room temperature. Data collected was processed using the Demeter software suite.

Zero-field ^57^Fe Mössbauer spectra were obtained at 80 K using a ^57^Co/rhodium source. Samples were prepared in an MBraun nitrogen glovebox. A typical powder sample containing approximately 60 mg of compounds was suspended in a plastic cap. Another cap with a slightly smaller diameter was squeezed into the previous sample cap to completely encapsulate the solid sample mixture. Frozen solution samples were prepared as concentrated solutions of ^57^Fe enriched **1** in toluene in the glovebox, removed from the glovebox under nitrogen, placed in a cold bath of −78 °C or −40 °C, and reacted with an excess of O_2_ which was bubbled through the solution. After reacting for the desired amount of time, the solution was exposed to air and pipetted with a precooled pipet into a plastic cap and frozen in liquid nitrogen. Another cap with a slightly smaller diameter was squeezed into the previous sample cap to completely encapsulate the frozen sample mixture. All spectra were analyzed using the WMOSS Mössbauer Spectral Analysis Software. Note that the accuracy of the fit parameters may be overestimated as the error in the Fe foil calibration is 0.01 mm/s.

### Fe(^*t*Bu,Tol^DHP-H_2_)(DMAP)Cl (**1**)

In a 20 mL vial in the glovebox, 3 mL of THF was added to FeCl_2_ (24 mg, 1 eq, 0.19 mmol). A solution of dimethylaminopyrrole (24 mg, 1 eq., 0.19 mmol) in 2 mL of THF was added to the FeCl_2_ suspension and stirred until a white suspension formed. The [^*t*Bu,Tol^DHP-H_4_][Cl]_2_ ligand salt^70^ (100 mg, 1 eq., 0.2 mmol) was dissolved in 5 mL of THF and added to the Fe solution to form a bright yellow suspension in a yellow solution. After being stirred for 10 min, KHMDS (104 mg, 2.7 eq., 0.521 mmol) dissolved in 1 mL THF was added dropwise with stirring. The solution turned from orange with a yellow precipitate, to colorless with a white precipitate, to colorless with no precipitate, to a deep orange-brown. Immediately after the addition of KHMDS and this sequence, the reaction mixture was condensed under a vacuum. The resulting brown solid was taken up in toluene, filtered, and condensed under a vacuum, then washed with petroleum ether (10 mL). After being dried, the pure bulk product was obtained as a pale orange solid. Yield: 90 mg, 71%. Single crystals suitable for XRD were grown via vapor diffusion of petroleum ether into a concentrated solution of product in toluene overnight at room temperature. ^1^H NMR (400 MHz, CD_3_CN, RT): δ = 29.2 (bs), 10.5 (bs), 8.6 (bs), 6.0 (bs), 5.7 (bs), – 3.4 (bs). Magnetic Susceptibility: Evans’ Method (C_6_D_6_, RT, μ_B_): μ_eff_ = 5.0; IR (Nujol mull between KBr plates, cm^–1^): 3180 (N–H, w), 3170 (N–H, w), 1641 (s). Mössbauer (80 K, mm/s) δ = 1.090(6); Δ*E*_Q_ = 2.367(9). UV–vis, nm in toluene, (ε, M^–1^cm^–1^): 516 (286). Anal. Calc. C, 64.07; H, 7.07; N, 14.94; Found: C, 64.65; H, 7.40; N, 14.03.

### Reactivity with PPh_3_, DHA, and Diphenylhydrazene (DPH)

A 0.35 mM solution of **1** in toluene was prepared in the glovebox in an airtight cuvette with a septa. After the solution was cooled to −40 °C, 0.5 mL of O_2_ was added via syringe, and the mixture was allowed to react until the absorbances for **3** had fully grown in. Then, 10 equiv of PPh_3_ were added as a solution in toluene via syringe and monitored over time with UV–visible spectroscopy. This same procedure was followed for DHA (10 equiv to a 0.35 mM solution of **1**) and DPH (20 equiv to a 0.42 mM solution of **1**) and neat 1,4-cyclohexadiene (CHD) (20 equiv to a 0.42 mM solution of **1**). This procedure was repeated with room temperature solutions of **1** with 20 equiv of PPh_3_ and DHA, and 10 equiv of DPH, respectively. The substrate was added 10 min after reacting with 6 mL of O_2_ to ensure that 3 had fully formed. When the reaction had finished bleaching, these reactions were analyzed by GC-MS. For CHD, **3** was generated cold, CHD was added, then the reaction was warmed and allowed to stir overnight before being analyzed by GC-MS.

#### Reactivity with PPh_3_ by NMR

An NMR solution was prepared with 5 mg of **1** in toluene (C_7_H_8_) with a septa NMR cap. This was then reacted with 10 equiv of PPh_3_ (added via syringe). Then, 6 mL of O_2_ was bubbled through the solution using a syringe at room temperature. This was allowed to react overnight at room temperature and then analyzed by ^31^P{^1^H} NMR.

### Reactivity with Diphenylisobenzofuran (DPBF)

In a 20 mL glass vial, **1** (14 mg, 1 equiv) was dissolved in toluene (2 mL) and sealed with a septa in the nitrogen glovebox. This was removed from the glovebox; 11 mL of O_2_ was added to the solution and the mixture was allowed to react for 15 min at room temperature. This was purged with 11 mL N_2_; DPBF (63 mg, 10.2 equiv) was added in the glovebox, and the reaction mixture was allowed to stir overnight. The solution was then filtered and analyzed by GC-MS.

### Deuteration of [^*t*Bu,Tol^DHP-H_4_][Cl]_2_ Ligand Salt

In a 20 mL vial, [^*t*Bu,Tol^DHP-H_4_][Cl]_2_ ligand salt (100 mg, 1 eq., 0.19 mmol) was dissolved in THF (10 mL). This mixture was cooled in a −35 °C freezer for 20 min. The solution was removed from the freezer and nBuLi (0.39 mL of a 2.5 M solution in diethyl ether, 5 eq., 0.96 mmol) was added dropwise with stirring at room temperature, causing the reaction to turn a deep red. This solution was allowed to stir for 5 min after which it was slowly warmed to room temperature, then DCl or *d*_4_-acetic acid (5 eq., 0.96 mmol) was added with stirring, causing the reaction to lighten to a golden yellow-orange. The reaction was condensed under a vacuum, taken up in toluene and filtered to remove LiCl or LiOAc, then recondensed. The resulting oil was taken up in THF (1 mL) and recrystallized via layer recrystallization with petroleum ether in the glovebox overnight. Yield: 50%. Percent enrichment by ^1^H NMR: 81 (DCl) or 93 (*d*_4_-acetic acid).

### Preparation of IR Samples of **3**

#### Concentrated solution in chlorobenzene

Complex **1** (10 mg) was placed in a 20 mL vial with a stir bar and 0.2 mL of chlorobenzene. A septa was used to seal the vial. This was removed from the glovebox, and O_2_ (0.39 mL, 1 equiv) was added via syringe, with the gas bubbled through the reaction mixture. This was immediately syringed into a solution cell IR, and a spectrum was collected.

#### Thin film on a KBr plate

Complex **1** (10 mg) was placed in a 20 mL vial with a stir bar and 0.2 mL of DCM. A septa was used to seal the vial. This was removed from the glovebox, and O_2_ (0.39 mL, 1 equiv) was added via syringe, with the gas bubbled through the reaction mixture. Using a syringe, the reaction mixture was removed from the vial, then one drop was placed on a KBr plate. Once DCM had evaporated, a second KBr plate was placed on top and a spectrum was collected.

#### Reaction in the solid state

Complex **1** (5 mg) was placed in a 20 mL vial with a stir bar and dry KBr powder (400 mg), mixed, and ground into a fine powder. A septa was used to seal the vial. This was removed from the glovebox, and an excess of O_2_ (3 mL) was added to the vial headspace. This was allowed to stir at room temperature for 1 h. The septa was removed; the mixture in KBr was used to form a KBr pellet, and a spectrum was collected.

### Preparation of Raman Samples of **3**

Complex **1** (7.5–14.5 mg) was placed in a 1 dram shell vial with 0.2 mL of DCM. This solution was divided drop by drop onto two separate glass slides and allowed to dry. The slides were placed carefully in a glass vessel, which was sealed with septa, and cooled externally with a dry ice/acetonitrile bath (−44 °C). This vessel was put under a vacuum, and 1.5 mL of O_2_ (excess) was syringed in under static vacuum. This was allowed to react in the solid state for an hour. A distinct color change was observed and corroborated by solid-state UV–vis spectroscopy (see below). It was then removed from the sealed vessel and immediately placed on a copper plate submerged in liquid nitrogen and a Raman spectrum collected with 532 nm laser on 10% power, 180 s acquisition times, 8–12 acquisitions, and 4X LWD objective.

To validate this method of preparing **3**, solid state UV–visible spectroscopy at −40 °C was undertaken. Compound **1** (0.002 g) was dissolved in DCM and allowed to evaporate on a cuvette placed on its side. This was sealed under nitrogen, transferred to the cooled UV–visible spectrometer, then reacted with excess O_2_. The reaction was monitored for 1 h until **3** had fully formed, as determined by the presence of features at 528 and 716 nm (Figure S15).

### Preparation of LC-MS Samples of **3**

Complex **1** (2.8 mg) was dissolved in 3 mL of toluene to form a 1.4 mM solution. This was monitored by UV–vis, and 1 mL was removed via cold syringe and placed into a chilled mass spectroscopy vial. This was then taken over cold and checked by LC-MS immediately.

## Supporting Information Available

Experimental procedures, NMR, IR, GC-MS UV–vis, EPR, XAS, SXRD data, and DFT (PDF)

## Accession Codes

CCDC 2094085 contains the supplementary crystallographic data for this paper. These data can be obtained free of charge via www.ccdc.cam.ac.uk/data_request/cif, or by emailing data_request@ccdc.cam.ac.uk, or by contacting The Cambridge Crystallographic Data Centre, 12 Union Road, Cambridge CB2 1EZ, UK; fax: +44 1223 336033.
