# Supplementary material for: Direct Aerobic Generation of a Ferric Hydroperoxo Intermediate Via a Preorganized Secondary Coordination Sphere
Source: J Am Chem Soc. 2021 Oct 26;143(43):18121–30. doi: 10.1021/jacs.1c06911 (PMC8569801; doi:10.1021/jacs.1c06911)
Supplement: Supplementary file 1 — ja1c06911_si_001.pdf [file ja1c06911_si_001.pdf]

Supplementary Information for:

Direct Aerobic Generation of a Ferric Hydroperoxo Intermediate via a Preorganized Secondary Coordination Sphere

Kate A. Jesse, Sophie W. Anferov, Kelsey A. Collins, Juan A. Valdez-Moreira, Maia E. Czaikowski, Alexander S. Filatov, John S. Anderson*

*corresponding author: jsanderson@uchicago.edu

Table of Contents

NMR spectroscopy 10

Figure S1. ^{1}H NMR of **1** in C_6D_6. 10

Figure S2. ^{1}H NMR of **1** in C_6D_6 over 8 days when stored as a solid at room temperature. 10

Figure S3. ^{1}H NMR of [^{tBu,Tol}DHP-H_4][Cl]$_2$ (bottom) stacked with [^{tBu,Tol}DHP-D_4][Cl]$_2$ (top, 93% enriched in deuterium) in C_6D_6. 11

Figure S4. ^{1}H NMR of [^{tBu,Tol}DHP-D_4][Cl]$_2$ in C_6D_6. The compound is 93% enriched in deuterium based on integrations of the beta N peak relative to its theoretical integration of 5 in [^{tBu,Tol}DHP-H_4][Cl]$_2$. 11

Figure S5. ^{31}P NMR of the reaction of **1** with 10 equivalents of PPh_3 at room temperature in toluene. Total integrated area is set to 10. 12

UV-vis spectroscopy 12

Figure S7. UV-vis of **4** bleaching at room temperature from a 0.35 mM solution of **1** in toluene. 13

Figure S8. UV-vis of **3** from a 0.35 mM solution of **1** in toluene with PPh_3 (10 eq.) at −40 °C. Scans are shown every hour over 3 hours. 13

Figure S9. UV-vis of **3** from a 0.42 mM solution of **1** in toluene with diphenylhydrazine (20 eq.) at -40 °C. Scans are shown every 10 minutes over 4 hours. 14

Figure S10. UV-vis of **3** from a 0.35 mM solution of **1** in toluene with DHA (10 eq. in 27 μL of toluene) at -40 °C. Gray traces move initially due to dilution, then are shown every 10 minutes for 30 minutes. 14

Figure S11. UV-vis of **3** from a 0.42 mM solution of **1** in toluene with cyclohexadiene (20 eq.) at -40 °C. Red trace: prior to substrate addition. Gray trace: 20 minutes after substrate addition. 15

Figure S12. UV-vis of **3** from a 0.35 mM solution of **1** in toluene at−40 °C after generation with 0.5 or 1 equivalent of O_2 as compared to when excess O_2 is used. 15

Figure S13. UV-vis of the reaction of 0.35 mM **1** in toluene with 2 equivalent of TEMPO$^{\bullet}$ after reacting overnight and **4**. 16

Figure S14. UV-vis of the reaction of 0.35 mM **1** in toluene with 1 equivalent of TEMPO$^{\bullet}$ with scans every 2 minutes stopping with the first species formed and **3**. 16

Figure S15. Solid state UV-vis of the reaction of 0.0022 g **1** reacted with excess O_2 as a thin film on the side of a cuvette at −40 °C. Black: Prior to the addition of O_2. Gray: Immediately following the addition of O_2. Red: 1 hour after the addition of O_2. 17

Vibrational Spectroscopy 17

Figure S16. IR spectrum of **1** in nujol. Inset: N–H stretches. 17

Figure S17. IR spectrum of **3** in a mixture of products as a thin film on KBr when formed using $^{16}O_2$ or $^{18}O_2$ at room temperature. 18

Figure S18. IR spectrum of **3** in a mixture of product as a thin film on KBr when formed using $^{16}O_2$ or $^{18}O_2$ at room temperature to look for an O–O stretch 19

Figure S19. IR spectrum of **3** in a mixture of products in a concentrated solution of chlorobenzene when formed using $^{16}O_2$ or $^{18}O_2$ at room temperature. 20

Figure S20. IR spectrum of **3** in a mixture of products in a concentrated solution of chlorobenzene when formed using $^{16}O_2$ or $^{18}O_2$ at room temperature to look for an O–O stretch. 21

Figure S21. IR spectrum of **3** in a KBr matrix as a mixture of products reacted from **1** in the solid state using $^{16}O_2$ or $^{18}O_2$ at room temperature... 22

Figure S22. IR spectrum of $^{16}O_2$ vs. $^{18}O_2$ reacted with **1** in the solid state to form **3** collected as a KBr pellet in the peroxo stretching region. 23

EPR Spectroscopy 24

Figure S23. EPR spectroscopy of a 15 mM solution of **3** in toluene at 15 K. Conditions: MW frequency, 9.381 GHz; MW power, 2.0 mW. 24

Figure S24. EPR spectroscopy of a 15 mM solution of **1** in toluene at 15 K. Conditions: MW frequency, 9.392 GHz; MW power, 2.0 mW. 24

Mössbauer Spectroscopy 25

Figure S25. Mössbauer spectrum of **1** with fits. (A) Prepared as a powder. (B) Prepared as a frozen solution in toluene using ^{57}Fe enriched complex **1**. (Bottom, left) Isomer shift and quadrapole splitting parameters and (bottom, right) legend. 25

Figure S26. Mössbauer spectrum of **2** with fits. Parameters for all fits used in overall data fitting. Samples were prepared as a frozen solution in toluene using ^{57}Fe enriched complex **1** that were reacted with O_2 for 6 minutes at −60 °C. 25

Figure S27. Mössbauer spectrum of **4** as a mixture with **3** with fits. Parameters for all fits used in overall data fitting. Samples were prepared as a frozen solution in toluene using ^{57}Fe enriched complex **1** and allowed to evolve from **3** via warming. 26

X-ray Absorption Spectroscopy 27

Figure S28. X-ray absorption spectra of **1** and **3** with K-edge inflection points of 7120 and 7124 eV respectively and pre-edge features at 7112 and 7114 eV respectively. **1** was collected as a solid powder at room temperature and **3** was collected a frozen solution in THF. Inset: Pre-edge features. 27

Single Crystal X-ray Diffraction 27

Figure S29. SXRD of **1** looking down the Cl-Fe-N3 bond (left) and looking down the Fe-N6 bond (right). Fe (orange), N (blue), C (gray), Cl (lime green), H (white). N-H protons were found in the difference map and refined. Selected bond lengths (Å). Fe1-Cl1: 2.2651(7), Fe1-N1: 2.399(2), Fe1-N3: 2.035(3), Fe1-N5: 2.362(2), Fe1-N6: 2.098(2), N1-N2: 1.416(3), N2-C8: 1.293(3), N4-N5: 1.410(3), N4-C13: 1.288(3), C10-C11: 1.390(4). Selected bond angles (°). N1-Fe1-N5: 152.93(8), N3-Fe1-Cl1: 153.00(1)... 28

Table S1. SXRD of **1** ... 28

Kinetic Measurements ... 29

Table S2. Calculated data for Eyring analysis of **2** to **3** at 996 nm. ... 29

Table S3. Rates of the reaction of **2** to **3** at 996 nm. ... 30

Table S4. Raw data for kinetic studies of **2** to **3** at −50 °C at 996 nm with a 0.7 mM solution in toluene. ... 30

Table S5. Raw data for kinetic studies of **2** to **3** at −55 °C at 996 nm with a 0.7 mM solution in toluene. ... 31

Table S6. Raw data for kinetic studies of **2** to **3** at −60 °C at 996 nm with a 0.7 mM solution in toluene. ... 32

Table S7. Raw data for kinetic studies of **2** to **3** at −65 °C at 996 nm with a 0.7 mM solution in toluene. ... 32

Table S8. Raw data for kinetic studies of **2** to **3** at −70 °C at 996 nm with a 0.7 mM solution in toluene. ... 33

Table S9. Raw data for kinetic studies of **2** to **3** at −65 °C at 996 nm with a 0.7 mM solution in toluene using deuterated ligand. ... 35

Table S10. Raw data for kinetic studies of **2** to **3** at −50 °C at 996 nm with a 0.7 mM solution in toluene using deuterated ligand. ... 35

Table S11. Rates of reaction for kinetic isotope effect calculation. ... 36

Figure S30. Rates of reaction for kinetic isotope effect experiment at various temperatures... 37

Density Functional Theory (DFT) ... 37

Figure S31. Calculated structure of **1**. All C–H hydrogen atoms have been removed for clarity. ... 38

Table S12. Calculated coordinates of **1**. ... 38

Figure S32. Calculated structure of a high spin Fe(III)(^{tBu,Tol}DHP-H$_2$)(DMAP)(Cl)(OO$^•$). All C–H hydrogen atoms have been removed for clarity. ... 40

Table S13. Calculated coordinates of high spin Fe(III)(^{tBu,Tol}DHP-H$_2$)(DMAP)(Cl)(OO$^•$)...... 40

Figure S33. Calculated structure of a high spin Fe(III)(^{tBu,Tol}DHP-H$^•$)(DMAP)(Cl)(OOH), **3**. All C–H hydrogen atoms have been removed for clarity. ... 43

Table S14. Calculated coordinates of high spin Fe(III)(^{tBu,Tol}DHP-H$^•$)(DMAP)(Cl)(OOH), **3**. 43

Figure S34. Spin density plot of **3** at an iso value of 0.003. ... 45

Figure S35. TDDFT of Fe(III)(^{tBu,Tol}DHP-H$_2$)(DMAP)(Cl)(OO$^•$) (left) and Fe(III)(^{tBu,Tol}DHP-H$^•$)(DMAP)(Cl)(OOH) (right), as compared to **3**. Note that TD-DFT typically underestimates the energies of transitions and a blue-shift to match experimental data of 50-75 nm is common in the related Ni-DHP complexes.[2] ... 46

Figure S36. Molecular orbitals involved in transitions contributing to states involved in the major features by UV-vis spectroscopy in 3 as calculated by TDDFT. Percentages to the right of the transition show the contribution of that transition to each calculated state. Only contributions above 10% are listed. 48

Table S15. Calculated vs. experimental values. 49

Gas Chromatography-Mass Spectrometry (GC-MS) 49

Figure S37. Mass spectrometry of the reaction of **1** with $^{16}O_2$ or $^{18}O_2$ and PPh_3 to form $OPPh_3$. 49

Figure S38. Conditions and resulting products observed by GC-MS. Yields are relative to 1 equivalent of **1.** 50

Figure S39. GC-MS the resulting product mixture of the reaction of **1** with excess O_2 and 10 equivalents of diphenylhydrazine (DPH) at 0 °C in toluene. 51

Figure S40. GC-MS the resulting product mixture of the reaction of **1** with excess O_2 and 20 equivalents of dihydroanthacene (DHA) at room temperature in toluene. 52

Figure S41. GC-MS the resulting product mixture of the reaction of **1** with excess O_2 in toluene at room temperature. 1-undecene (10.4 equivalents) was also included as an internal standard for GC-MS 53

Figure S42. GC-MS of the reaction of **1** with $^{16}O_2$ or $^{18}O_2$ and DPBF to form 9-hydroxyanthracen-10(9H)-one. 54

Electrospray Ionization-Mass Spectrometry (ESI-MS 55

Figure S43. ESI-MS of the reaction of **1** with O_2 at −40 °C to form **3**. The peak at 282 m/z is consistent with **3** where the DMAP auxiliary ligand has dissociated and flies as a dication. Additional relevant peaks and their assignment are listed. Blue: $^{16}O_2$. Brown: $^{18}O_2$. 55

Figure S44. ESI-MS of the reaction of **1** with O_2 at −40 °C to form **4** (top). The peak at 534 m/z is consistent with **4** where the DMAP auxiliary ligand has dissociated. Additionally, the features associated with **3** have decreased in intensity when comparing the ESI-MS of **3** (bottom) to **4** (top). 55

References 56

General Methods

All chemicals were purchased from commercial suppliers and used without further purification. All manipulations were carried out under an atmosphere of N_2 using standard Schlenk and glovebox techniques. Glassware was dried at 180 °C for a minimum of two hours and cooled under vacuum prior to use. Solvents were dried on a solvent purification system from Pure Process Technologies and stored over 4 Å molecular sieves under N_2. Tetrahydrofuran (THF) was stirred over NaK alloy and run through an additional alumina column prior to use to ensure dryness. Solvents were tested for H_2O and O_2 using a standard solution of sodium-benzophenone ketyl radical anion. CD_3CN, C_6D_6, and d_8-toluene were dried over 4 Å molecular sieves under N_2.

^1H and $^{31}P\{^1H\}$ NMR spectra were recorded on Bruker DRX 400 or 500 spectrometers. Chemical shifts are reported in ppm units referenced to residual solvent resonances for ^1H and $^{31}H\{^1H\}$ spectra. UV-Visible Spectra were recorded on a Bruker Evolution 300 spectrometer and analyzed using VisionPro software. IR spectra were obtained on a Bruker Tensor II spectrometer with the OPUS software suite. All IR samples were prepared nujol mulls or collected between KBr plates. EPR spectra were recorded on an Elexsys E500 Spectrometer with an Oxford ESR 900 X-band cryostat and a Bruker Cold-Edge Stinger. EPR data was analyzed using SpinCount. Single crystal X-ray diffraction data were collected in-house using Bruker D8 Venture diffractometer equipped with Mo microfocus X-ray tube (λ = 0.71073 Å).

X-ray near-edge absorption spectra (XANES) were employed to probe the local environment of Fe. Powder samples were prepared by material grinding finely. A Teflon window was sealed on one side with Kapton tape and powder was then transfer transferred to the inside of this ring before compacting with a Teflon rod and sealing the remaining face with Kapton tape. After transfer of the material, the window was sealed with Kapton tape. All sample preparation was performed under an inert atmosphere. Frozen solution samples were prepared by making a concentrated solution in THF of the starting material, removing the sample from the glovebox, cooling the sample in a bath, then reacting the sample with O_2 by syringing the gas into the sample and bubbling through. After allowing to react, the sample was exposed to air and precooled pipette was used to transfer the solution to a Teflon window lined on one side with Kapton tape. The solution was frozen using liquid nitrogen, then stored in liquid nitrogen until collection. Data were acquired at the Advanced Photon Source at Argonne National Labs with a bending magnet source with ring energy at 7.00 GeV. Fe K-edge data were acquired at the MRCAT 10-BM beam line. The incident, transmitted and reference X-ray intensities were monitored using gas ionization chambers. A metallic iron foil standard was used as a reference for energy calibration and was measured simultaneously with experimental samples. X-ray absorption spectra were collected at room temperature. Data collected was processed using the Demeter software suite.

Zero-field ^{57}Fe Mössbauer spectra were obtained at 80 K using a ^{57}Co/rhodium source. Samples were prepared in an MBraun nitrogen glove box. A typical powder sample contained approximately 60 mg of compounds suspended in a plastic cap. Another cap with a slightly smaller diameter was squeezed into the previous sample cap to completely encapsulate the solid sample mixture. Frozen solution samples were prepared as concentrated solutions of ^{57}Fe enriched **1** in toluene in the glovebox, removed from the glovebox under nitrogen, placed in a cold bath of −78

°C or −40 °C, and reacted with an excess of O_2 which was bubbled through the solution. After reacting for the desired amount of time, the solution was exposed to air and pipetted with a precooled pipette into a plastic cap and frozen in liquid nitrogen. Another cap with a slightly smaller diameter was squeezed into the previous sample cap to completely encapsulate the frozen sample mixture. All spectra were analyzed using the WMOSS Mössbauer Spectral Analysis Software. Note that the accuracy of the fit parameters may be overestimated as the error in the Fe foil calibration is 0.01 mm/s.

Fe($^{\mathrm{Tol,tBu}}$DHP-H_2)(DMAP)Cl (1)

In a 20 mL vial in the glovebox, 3 mL of THF was added to $FeCl_2$ (24 mg, 1 eq, 0.19 mmol). A solution of dimethylaminopyrrole (24 mg, 1 eq., 0.19 mmol) in 2 mL of THF was added to the $FeCl_2$ suspension and stirred until a white suspension formed. The [$^{\mathrm{Tol,tBu}}$DHP-H_4][Cl]$_2$ ligand salt,[71] (100 mg, 1 eq., 0.2 mmol) was dissolved in 5 mL THF and added to the Fe solution to form a bright yellow suspension in a yellow solution. After stirring for 10 minutes, KHMDS (104 mg, 2.7 eq., 0.521 mmol) dissolved in 1 mL THF was added dropwise with stirring. The solution turned from orange with a yellow precipitate, to colorless with a white precipitate, to colorless with no precipitate, to a deep orange-brown. Immediately after the addition of KHMDS and this sequence, the reaction mixture was condensed under vacuum. The resulting brown solid was taken up in toluene, filtered, and condensed under vacuum, then washed with petroleum ether (10 mL). After drying, the pure bulk product was obtained as a pale orange solid. Yield: 90 mg, 71%. Single crystals suitable for XRD were grown via vapor diffusion of petroleum ether into a concentrated solution of product in toluene overnight at room temperature. ^{1}H NMR (400 MHz, CD_3CN, RT): δ = 29.2 (bs), 10.5 (bs), 8.6 (bs), 6.0 (bs), 5.7 (bs), −3.4 (bs). Magnetic Susceptibility: Evans' Method (C_6D_6, RT, μ_B): μ_{eff} = 5.0; IR (Nujol mull between KBr plates, cm^{-1}): 3180 (N–H, w), 3170 (N–H, w), 1641 (s). Mössbauer (80 K, mm/s) δ = 1.090(6); ΔE_Q = 2.367(9). UV-vis, nm in toluene, (ε, $M^{-1}cm^{-1}$): 516 (286). Anal. Calc. C, 64.07; H, 7.07; N, 14.94; Found: C, 64.65; H, 7.40; N, 14.03.

Reactivity with PPh_3, DHA, and diphenylhydrazene (DPH)

A 0.35 mM solution of **1** in toluene was prepared in the glovebox in an air-tight cuvette with a septa. After cooling to −40 °C, 0.5 mL of O_2 was added via syringe and allowed to react until the absorbances for **3** had fully grown in. Then, 10 equivalents of PPh_3 were added as a solution in toluene via syringe and monitored over time with UV-visible spectroscopy. This same procedure was followed for DHA (10 equivalents to a 0.35 mM solution of **1**) and DPH (20 equivalents to a 0.42 mM solution of **1**) and neat 1,4-cyclohexadiene (CHD) (20 equivalents to a 0.42 mM solution of **1**). This procedure was repeated with room temperature solutions of **1** with 20 equivalents of PPh_3 and DHA, and 10 equivalents of DPH respectively. The substrate was added 10 minutes after reacting with 6 mL of O_2 to ensure that 3 had fully formed. When the reaction had finished bleaching, these reactions were analyzed by GC-MS. For CHD, **3** was generated cold, CHD was added, then the reaction was warmed and allowed to stir overnight before being analyzed by GC-MS.

Reactivity with PPh_3 by NMR. An NMR solution was prepared with 5 mg of **1** in toluene (C_7H_8) with a septa NMR cap. This was then reacted with 10 equivalents of PPh_3 (added via syringe). Then, 6 mL of O_2 was bubbled through the solution using a syringe at room temperature. This was allowed to react overnight at room temperature, then analyzed by $^{31}P\{^1H\}$ NMR.

Reactivity with diphenylisobenzofuran (DPBF)

In a 20 mL glass vial, **1** (14 mg, 1 eq.) was dissolved in toluene (2 mL) and sealed with a septa in the nitrogen glovebox. This was removed from the glovebox and 11 mL of O_2 was added and allowed to react for 15 minutes at room temperature. This was purged with 11 mL N_2, then DPBF (63 mg, 10.2 eq.) was added in the glovebox and the reaction mixture was allowed to stir overnight. The solution was then filtered and analyzed by GC-MS.

Deuteration of the [^{Tol,tBu}DHP-H$_4$][Cl]$_2$ ligand salt

In a 20 mL vial, [^{Tol,tBu}DHP-H$_4$][Cl]$_2$ ligand salt (100 mg, 1 eq., 0.19 mmol) was dissolved in THF (10 mL). This was cooled in a −35 °C freezer for 20 minutes. The solution was removed from the freezer and nBuLi (0.39 mL of a 2.5 M solution in diethylether, 5 eq., 0.96 mmol) was added dropwise with stirring at room temperature, causing the reaction to turn a deep red. This solution was allowed to stir for 5 minutes after which it was slowly warmed to room temperature, then DCl or *d_4*-acetic acid (5 eq., 0.96 mmol) was added with stirring, causing the reaction to lighten to a golden yellow-orange. The reaction was condensed under vacuum, taken up in toluene and filtered to remove LiCl or LiOAc, then recondensed. The resulting oil was taken up in THF (1 mL) and recrystallized via layer recrystallization with petroleum ether in the glovebox overnight. Yield: 50%. Percent enrichment by ^{1}H NMR: 81 (DCl) or 93 (*d_4*-acetic acid).

Preparation of IR samples of 3

Concentrated solution in chlorobenzene: Complex **1** (10 mg) was placed in a 20 mL vial with a stir bar and 0.2 mL of chlorobenzene. A septa was used to seal the vial. This was removed from the glovebox and O_2 (0.39 mL, 1 eq) was added via syringe, with the gas bubbled through the reaction mixture. This was immediately syringed into a solution cell IR and a spectrum was collected.

Thin film on a KBr plate: Complex **1** (10 mg) was placed in a 20 mL vial with a stir bar and 0.2 mL of DCM. A septa was used to seal the vial. This was removed from the glovebox and O_2 (0.39 mL, 1 eq) was added via syringe, with the gas bubbled through the reaction mixture. Using a syringe, the reaction mixture was removed from the vial, then one drop was placed on a KBr plate. Once DCM had evaporated, a second KBr plate was placed on top and a spectrum was collected.

Reaction in the solid state: Complex **1** (5 mg) was placed in a 20 mL vial with a stir bar and dry KBr powder (400 mg), mixed, and ground into a fine powder. A septa was used to seal the vial. This was removed from the glovebox and an excess of O_2 (3 mL) was added to the vial headspace. This was allowed to stir at room temperature for one hour. The septa was removed and the mixture in KBr was used to form a KBr pellet and a spectrum was collected.

Preparation of Raman samples of 3

Complex **1** (7.5-14.5 mg) was placed in a 1 dram shell vial with 0.2 mL of DCM. This solution was divided drop by drop onto two separate glass slides and allowed to dry. The slides were placed carefully in a glass vessel, which was sealed with septa, and cooled externally with a dry ice/acetonitrile bath (−44ºC). This vessel was put under vacuum, and 1.5 mL of O_2 (excess) was syringed in under static vacuum. This was allowed to react in the solid state for an hour. A distinct color change was observed and corroborated by a solid-state UV-Vis. It was then removed from the sealed vessel and immediately placed on the copper plate cooled by liquid nitrogen and a Raman spectrum collected with 532 nm laser on 10% power, 180 s acquisition times, 8-12 acquisitions, and 4X LWD objective.

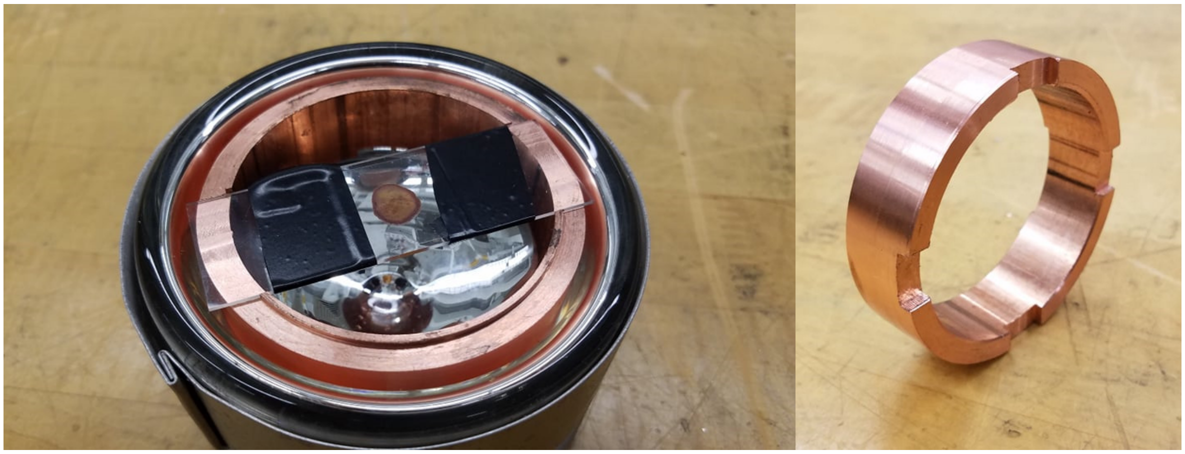

To validate this method of preparing **3**, solid state UV-visible spectroscopy at −40 °C. 0.0022 g of **1** was dissolved in DCM and allowed to evaporate on a cuvette in the lateral decubitus position. This was sealed under nitrogen, transferred to the cooled UV-visible spectrometer, then reacted with excess O_2. This was monitored for 1 hour until **3** had fully formed, as determined by the presence of features at 528 nm and 716 nm.

Preparation of LC-MS samples of 3

Complex **1** (2.8 mg) was dissolved in 3 mL of toluene to form a 1.4 mM solution. This was monitored by UV-Vis and 1 mL removed via cold syringe and placed into a chilled mass spectroscopy vial. This was then taken over cold and checked by LC-MS immediately.

NMR spectroscopy

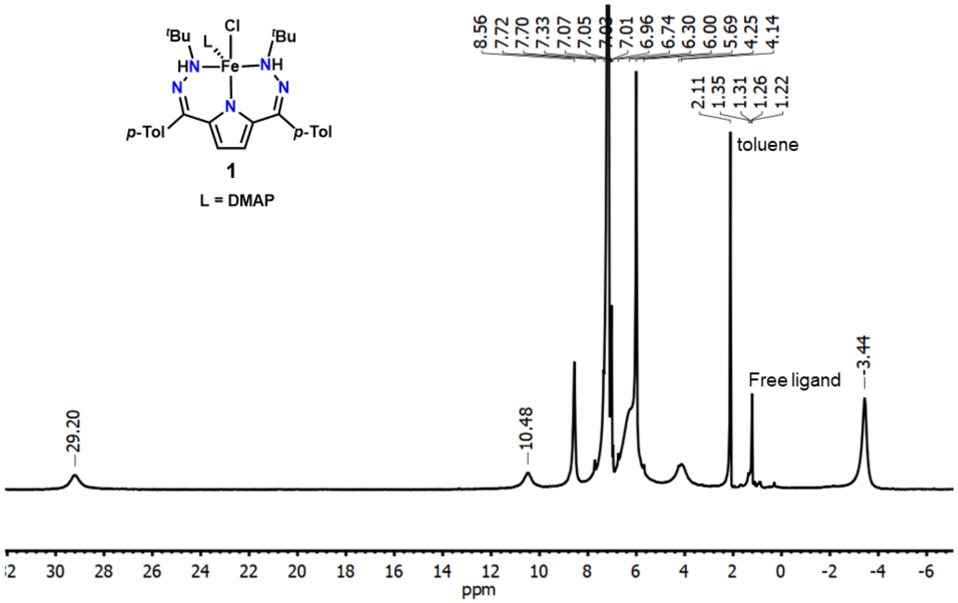

Figure S1. ^{1}H NMR of **1** in C_6D_6.

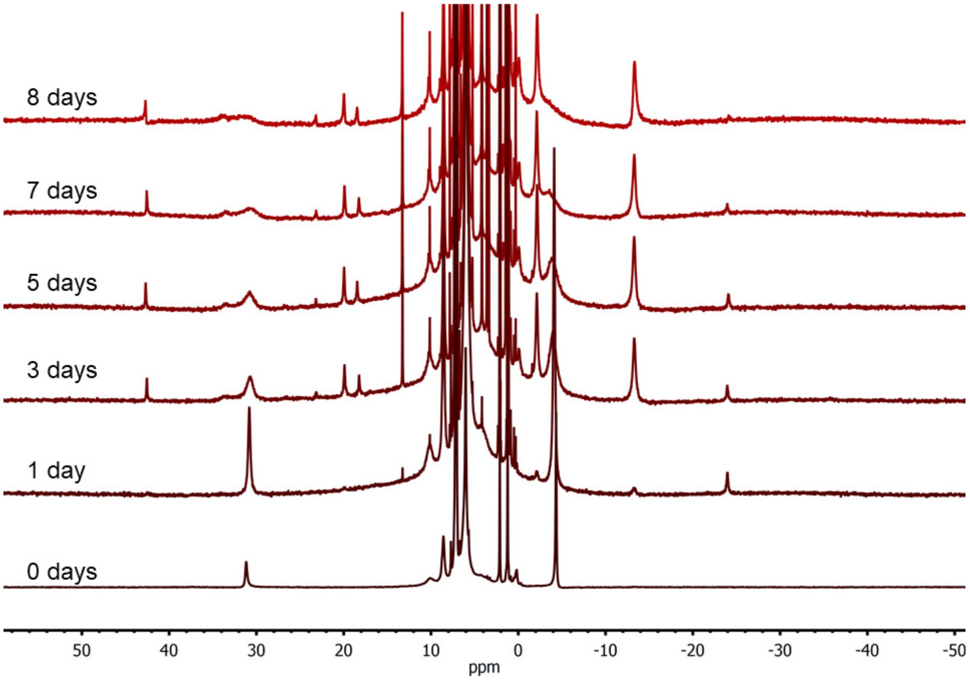

Figure S2. ^{1}H NMR of **1** in C_6D_6 over 8 days when stored as a solid at room temperature.

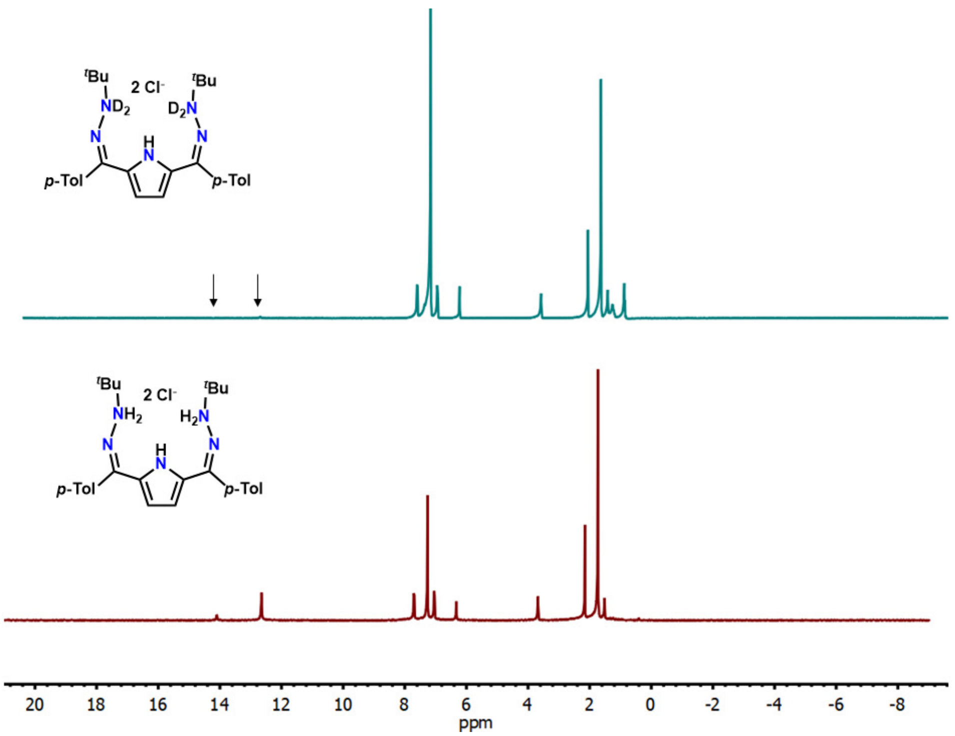

Figure S3. ^{1}H NMR of [^{tBu,Tol}DHP-H$_4$][Cl]$_2$ (bottom) stacked with [^{tBu,Tol}DHP-D$_4$][Cl]$_2$ (top, 93% enriched in deuterium) in C_6D_6.

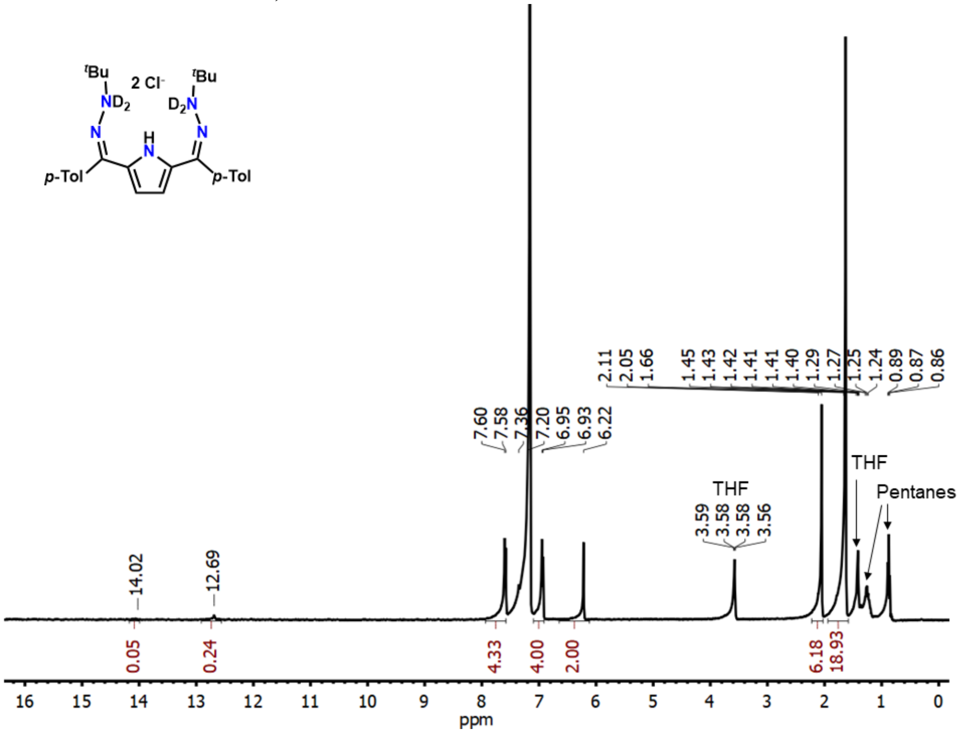

Figure S4. ^{1}H NMR of [^{tBu,Tol}DHP-D$_4$][Cl]$_2$ in C_6D_6. The compound is 93% enriched in deuterium based on integrations of the beta N peak relative to its theoretical integration of 5 in [^{tBu,Tol}DHP-H$_4$][Cl]$_2$.

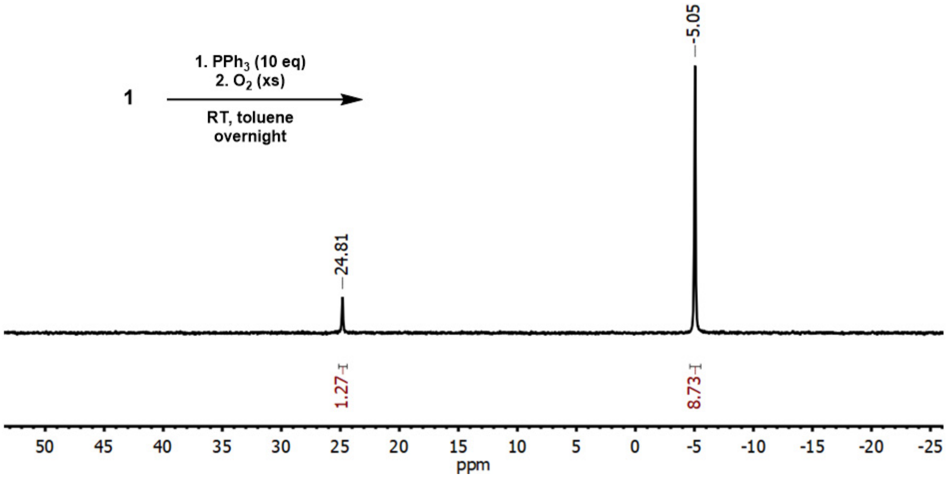

Figure S5. ${}^{31}P$ NMR of the reaction of **1** with 10 equivalents of PPh_3 at room temperature in toluene. Total integrated area is set to 10.

UV-vis spectroscopy

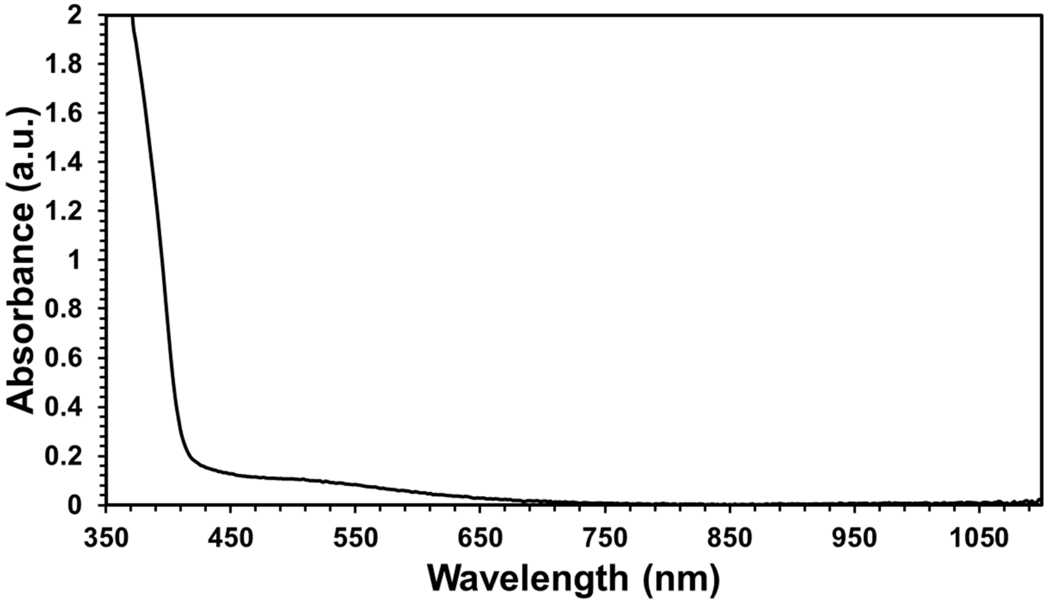

Figure S6. UV-vis of **1** as a 0.35 mM solution in toluene.

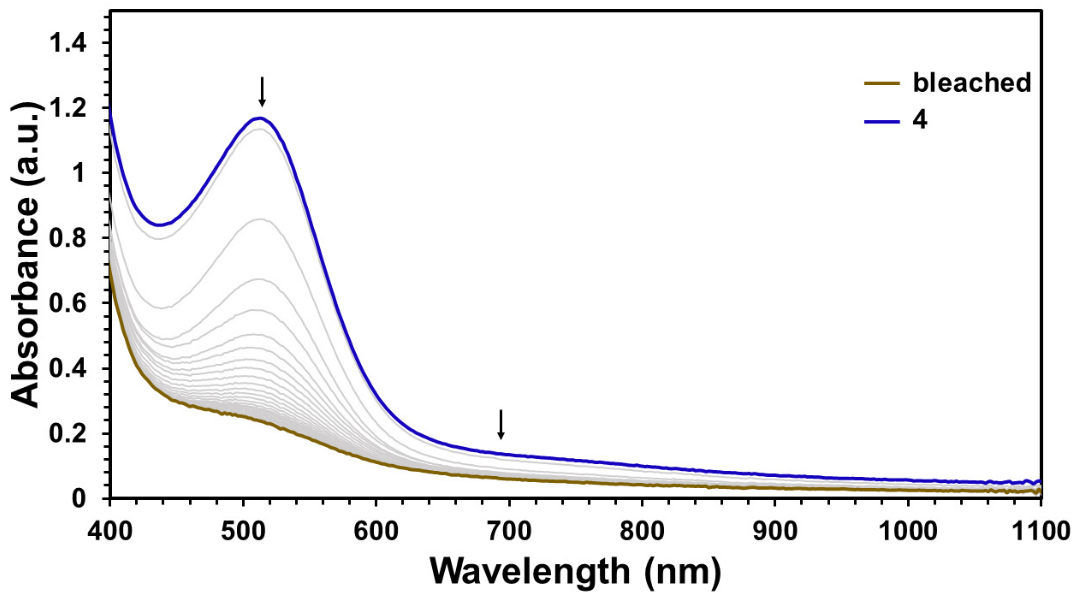

Figure S7. UV-vis of **4** bleaching at room temperature from a 0.35 mM solution of **1** in toluene.

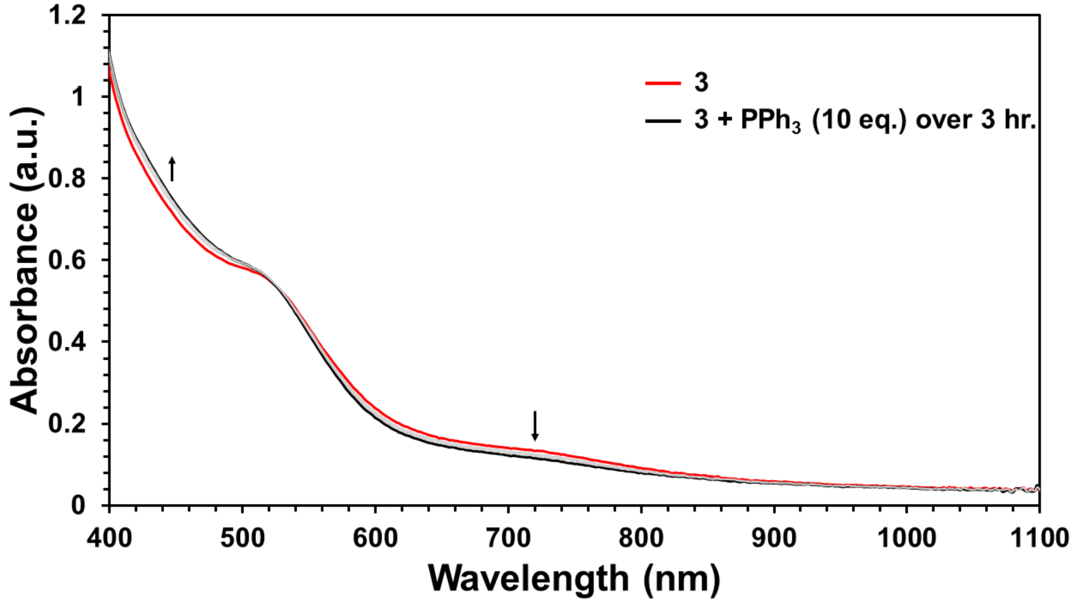

Figure S8. UV-vis of **3** from a 0.35 mM solution of **1** in toluene with PPh_3 (10 eq.) at −40 °C. Scans are shown every hour over 3 hours.

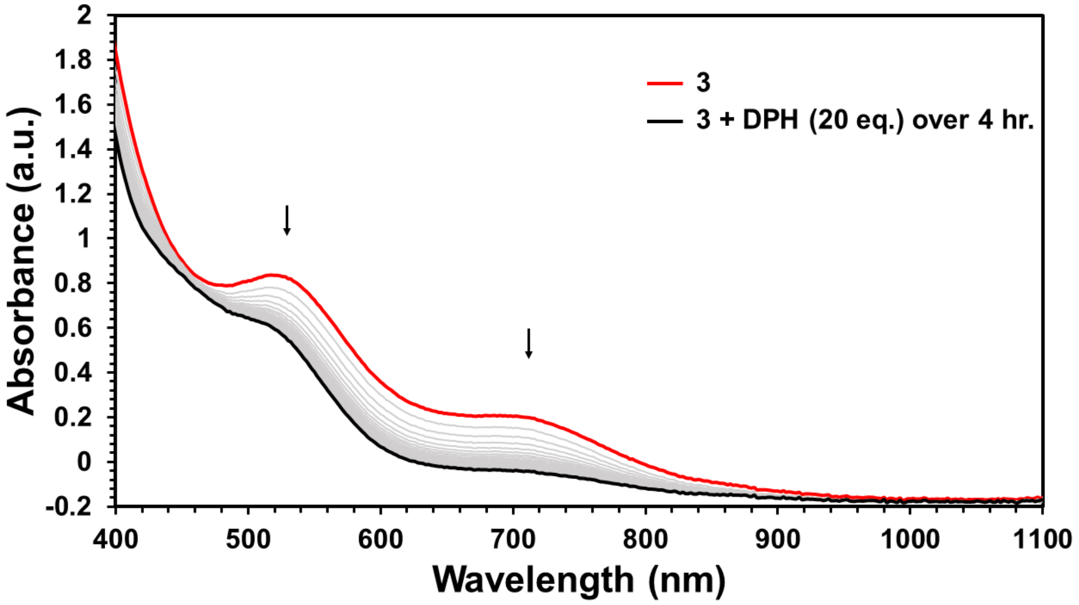

Figure S9. UV-vis of **3** from a 0.42 mM solution of **1** in toluene with diphenylhydrazine (20 eq.) at -40 °C. Scans are shown every 10 minutes over 4 hours.

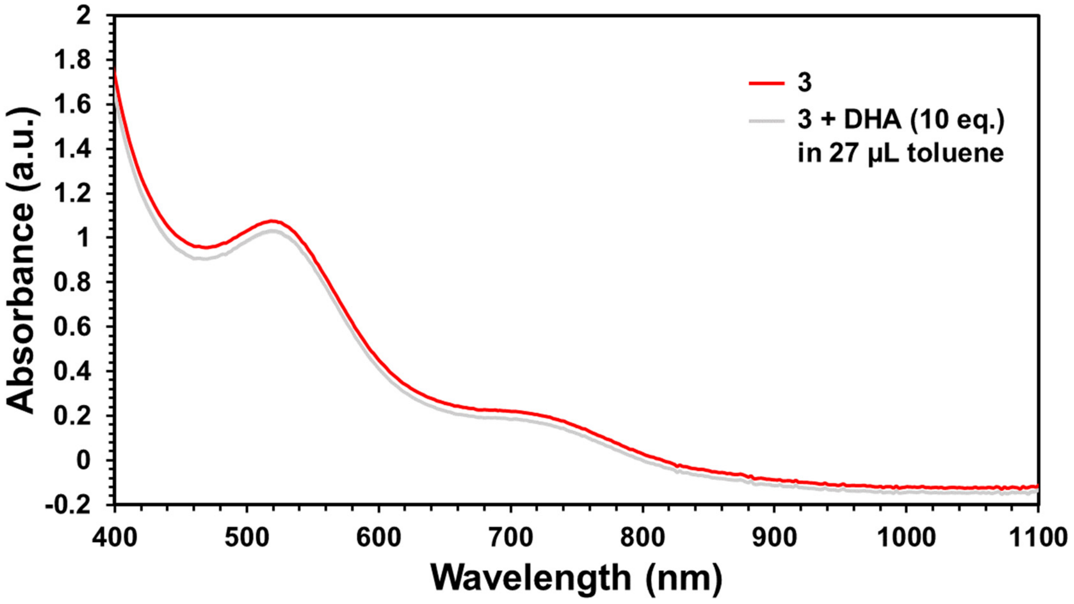

Figure S10. UV-vis of **3** from a 0.35 mM solution of **1** in toluene with DHA (10 eq. in 27 µL of toluene) at -40 °C. Gray traces move initially due to dilution, then are shown every 10 minutes for 30 minutes.

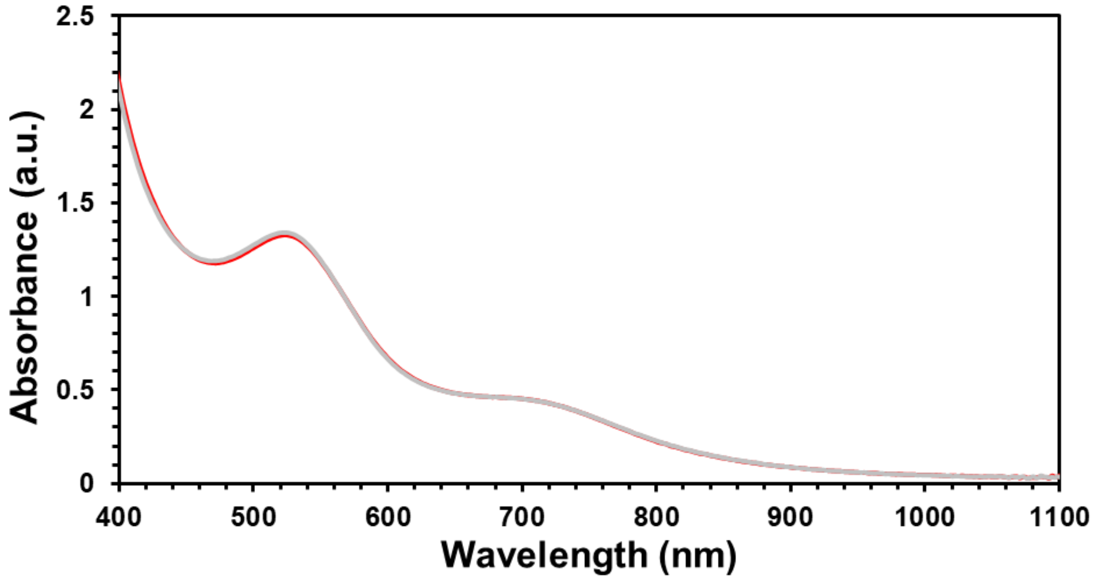

Figure S11. UV-vis of **3** from a 0.42 mM solution of **1** in toluene with cyclohexadiene (20 eq.) at -40 °C. Red trace: prior to substrate addition. Gray trace: 20 minutes after substrate addition.

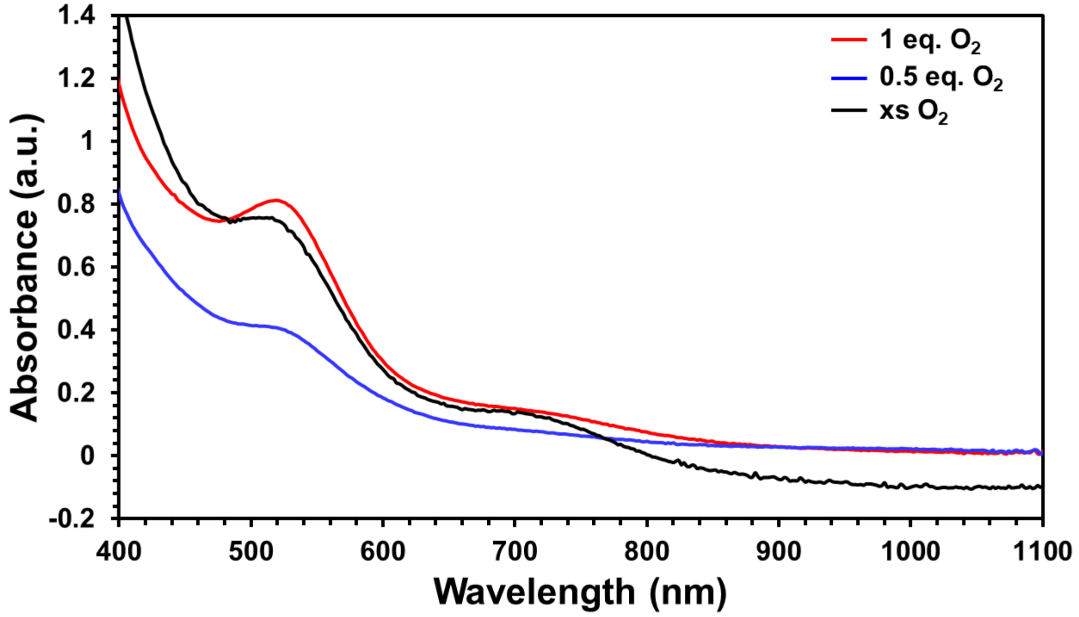

Figure S12. UV-vis of **3** from a 0.35 mM solution of **1** in toluene at−40 °C after generation with 0.5 or 1 equivalent of O_2 as compared to when excess O_2 is used.

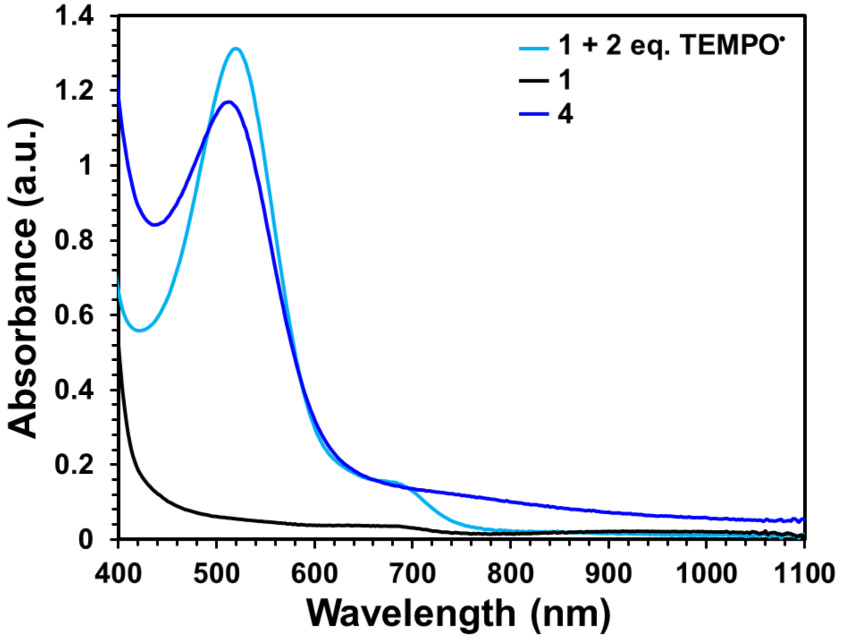

Figure S13. UV-vis of the reaction of 0.35 mM **1** in toluene with 2 equivalent of TEMPO• after reacting overnight and **4**.

This shows that the second species which forms has features in the same locations as **4**, suggesting that they may be the same species. Eventually, bleaching was observed, which is again consistent with the second species being the same as **4**, which bleaches when allowed to sit at room temperature.

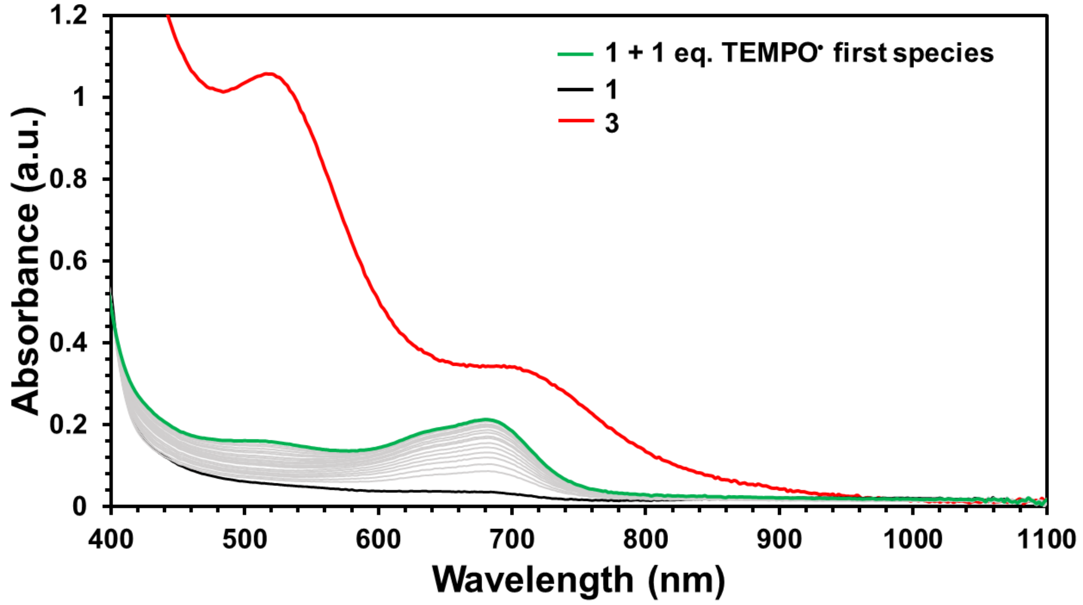

Figure S14. UV-vis of the reaction of 0.35 mM **1** in toluene with 1 equivalent of TEMPO• with scans every 2 minutes stopping with the first species formed and **3**.

This shows that the first species which forms has features that do not align with **3**, suggesting that they are not the same species.

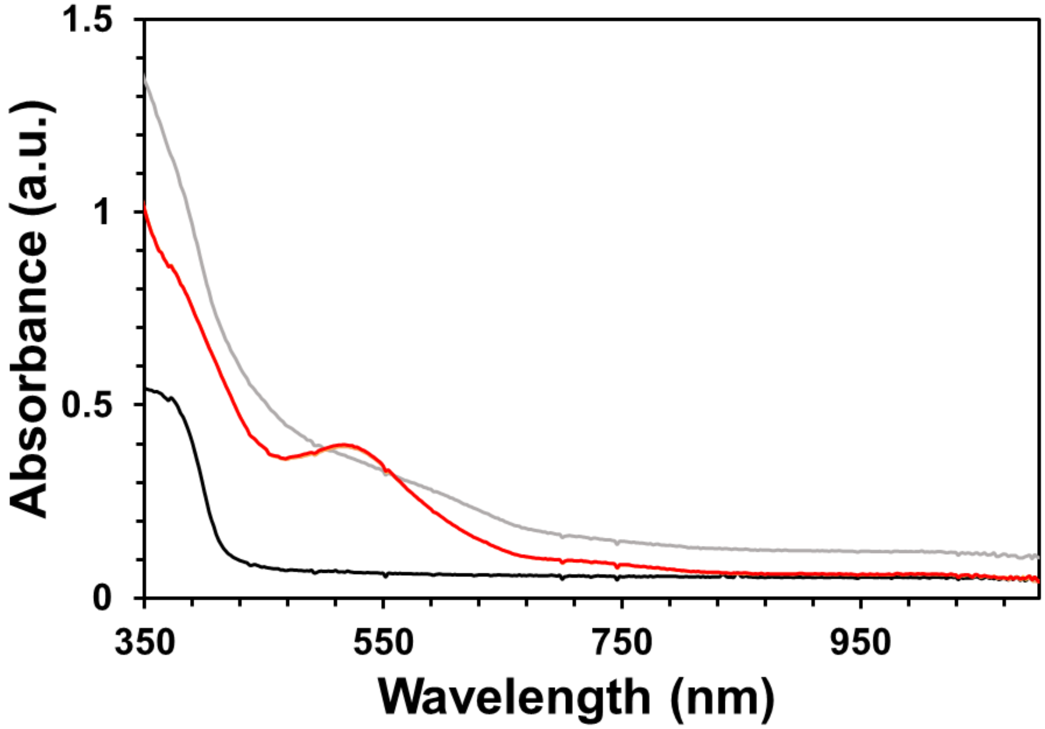

Figure S15. Solid state UV-vis of the reaction of 0.0022 g **1** reacted with excess O_2 as a thin film on the side of a cuvette at −40 °C. Black: Prior to the addition of O_2. Gray: Immediately following the addition of O_2. Red: 1 hour after the addition of O_2.

Vibrational Spectroscopy

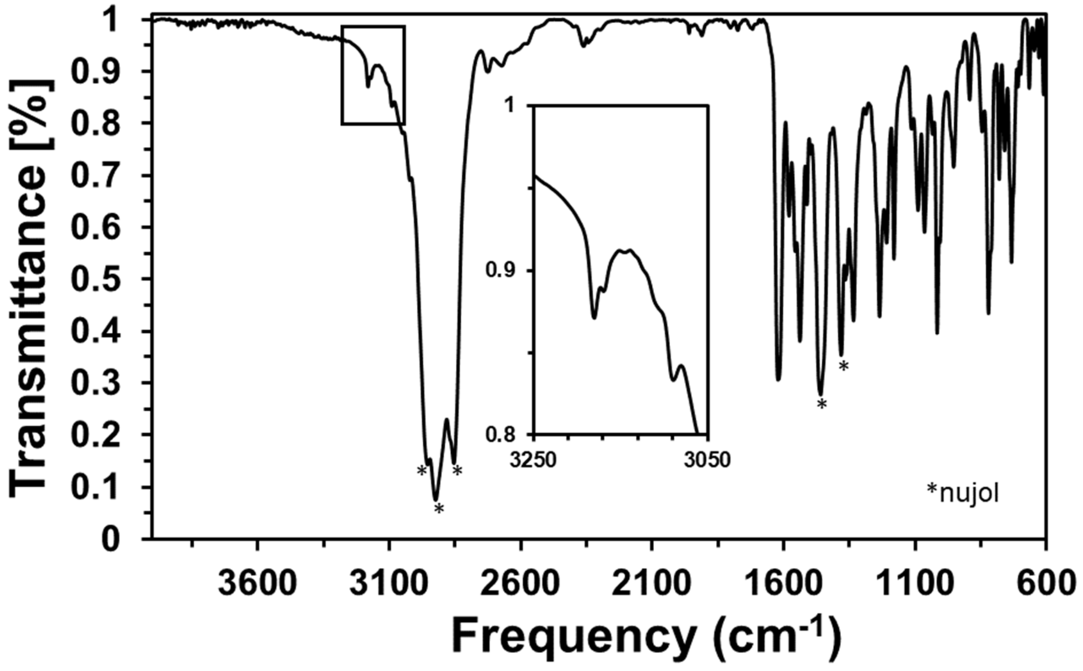

Figure S16. IR spectrum of **1** in nujol. Inset: N–H stretches.

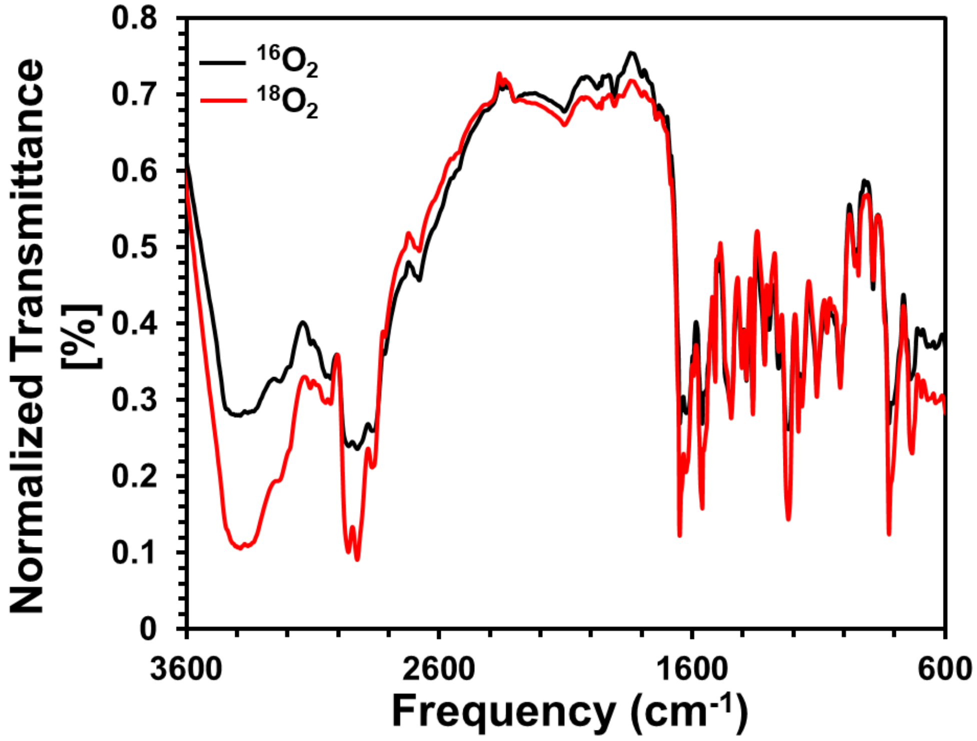

Figure S17. IR spectrum of **3** in a mixture of products as a thin film on KBr when formed using $^{16}O_2$ or $^{18}O_2$ at room temperature.

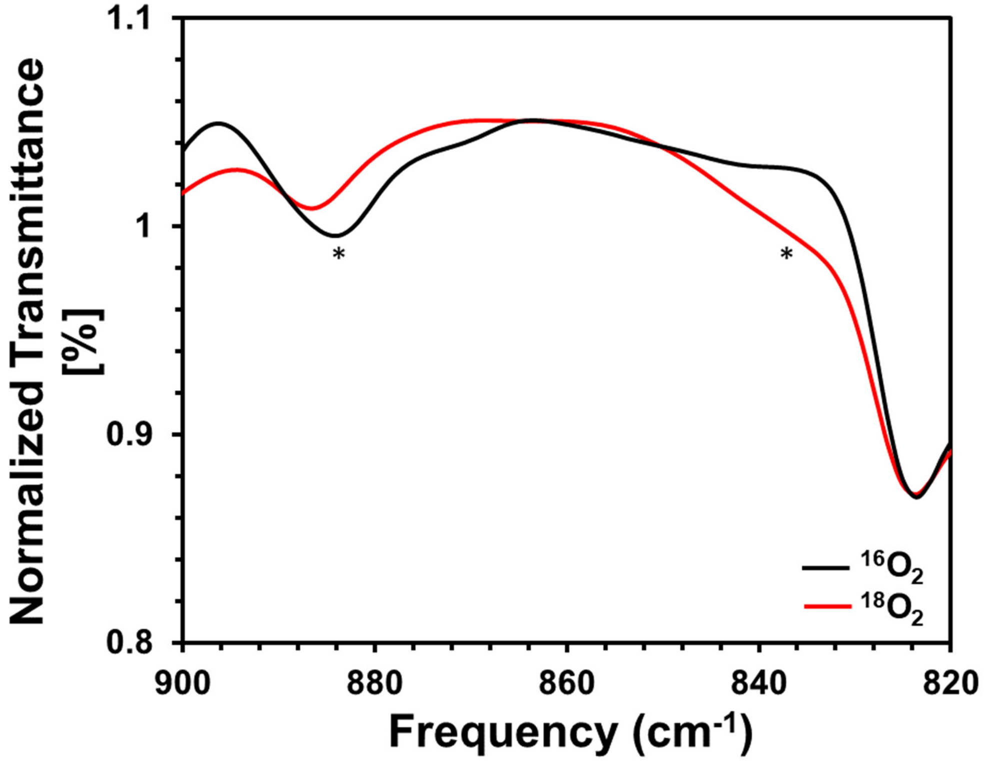

Figure S18. IR spectrum of **3** in a mixture of product as a thin film on KBr when formed using $^{16}O_2$ or $^{18}O_2$ at room temperature to look for an O–O stretch

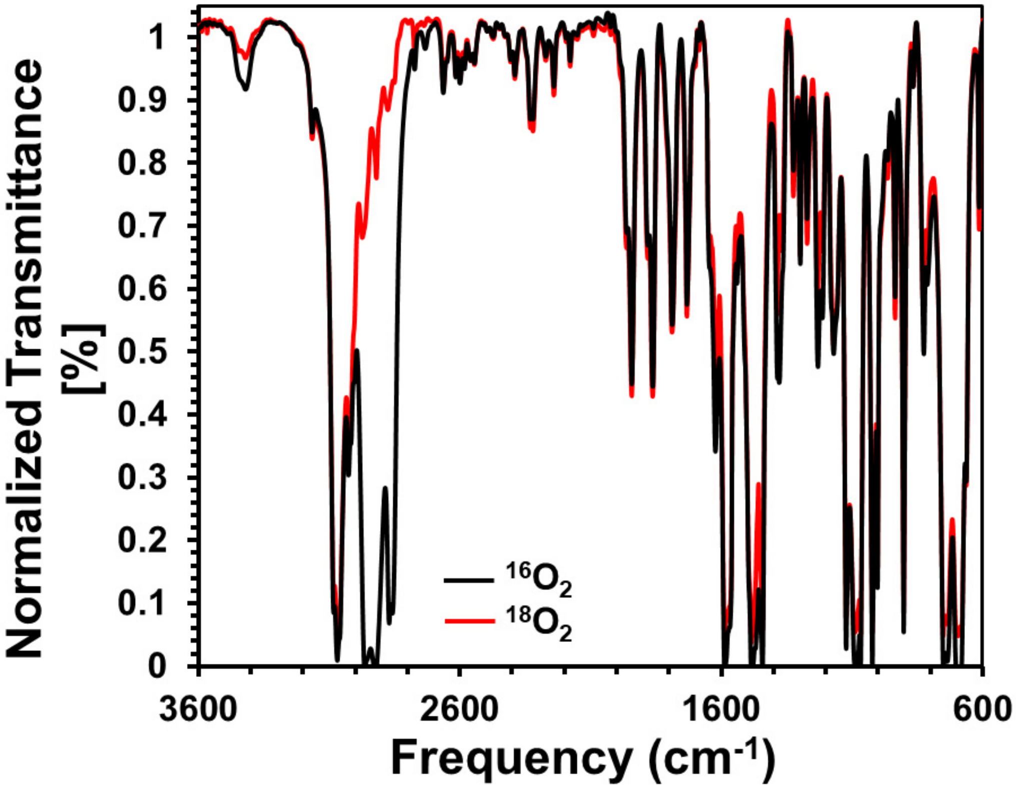

Figure S19. IR spectrum of **3** in a mixture of products in a concentrated solution of chlorobenzene when formed using $^{16}O_2$ or $^{18}O_2$ at room temperature.

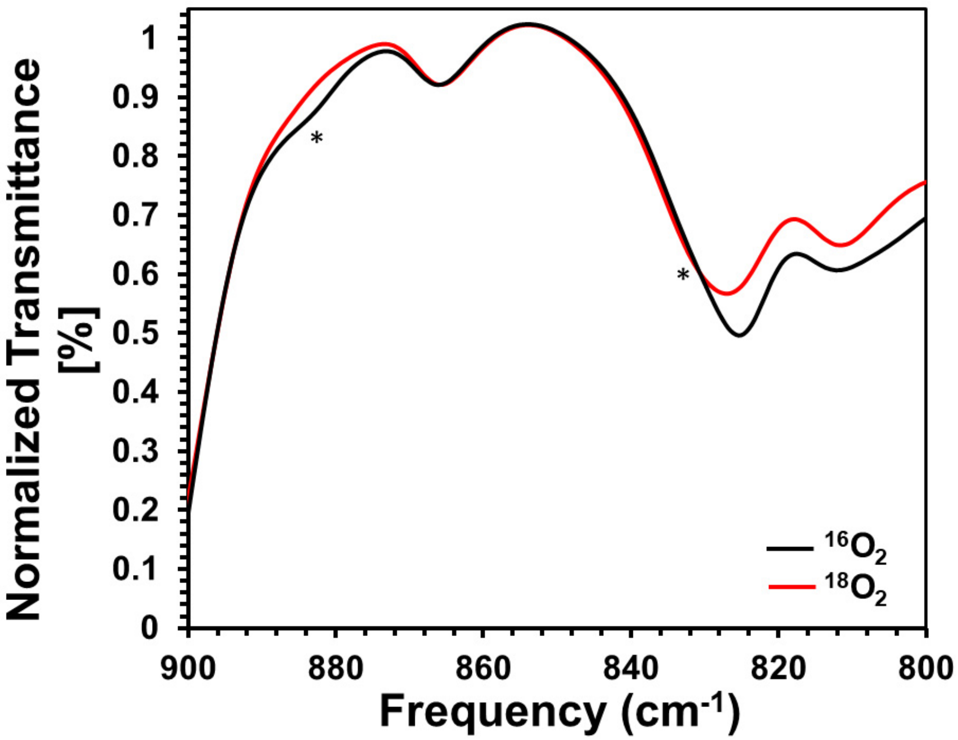

Figure S20. IR spectrum of **3** in a mixture of products in a concentrated solution of chlorobenzene when formed using $^{16}O_2$ or $^{18}O_2$ at room temperature to look for an O–O stretch.

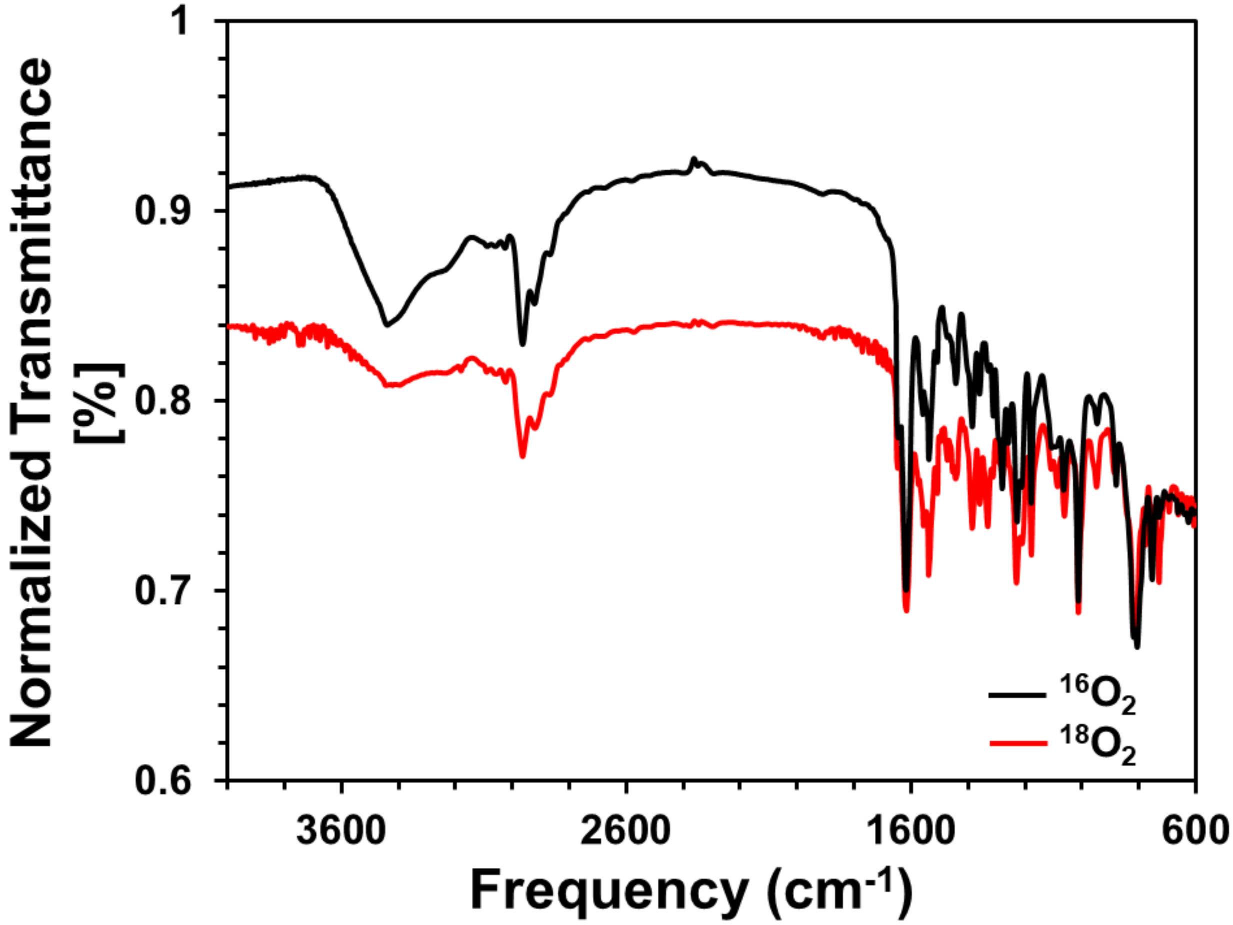

Figure S21. IR spectrum of **3** in a KBr matrix as a mixture of products reacted from **1** in the solid state using $^{16}O_2$ or $^{18}O_2$ at room temperature.

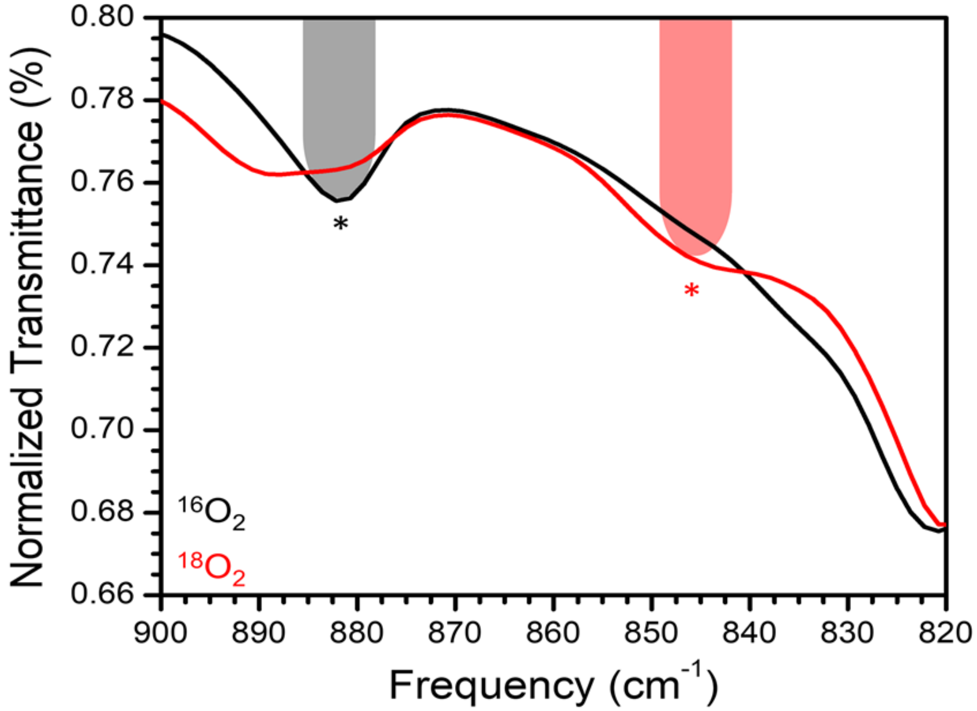

Figure S22. IR spectrum of $^{16}O_2$ vs. $^{18}O_2$ reacted with **1** in the solid state to form **3** collected as a KBr pellet in the peroxo stretching region.

EPR Spectroscopy

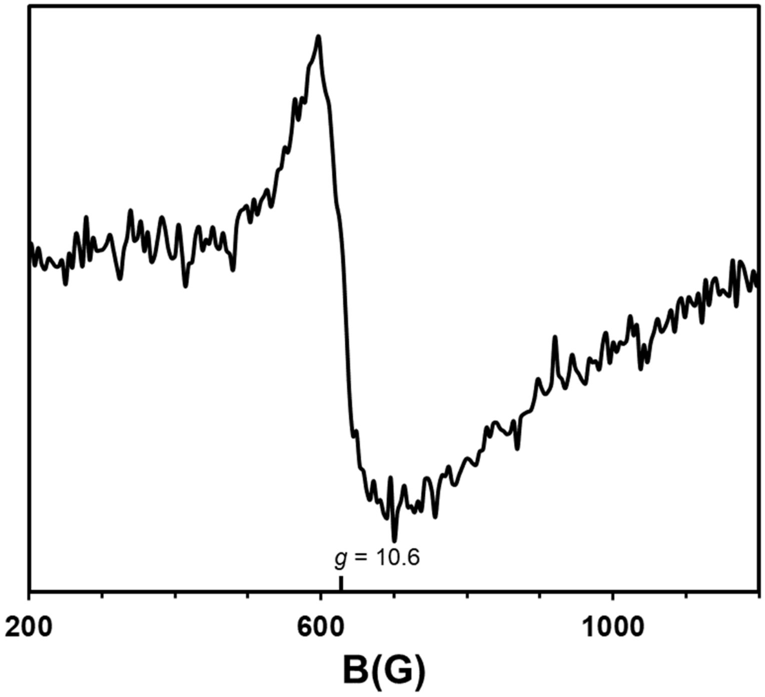

Figure S23. EPR spectroscopy of a 15 mM solution of **3** in toluene at 15 K. Conditions: MW frequency, 9.381 GHz; MW power, 2.0 mW.

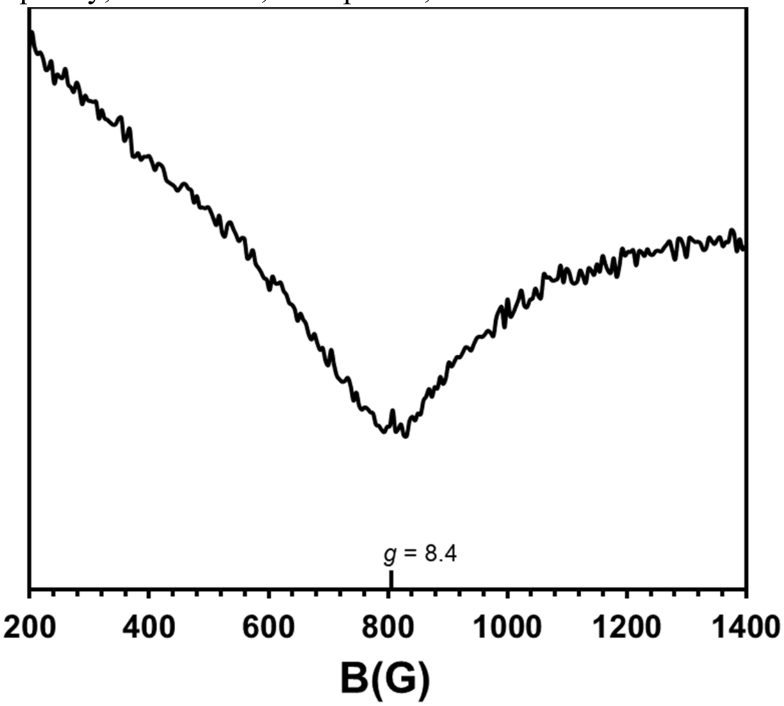

Figure S24. EPR spectroscopy of a 15 mM solution of **1** in toluene at 15 K. Conditions: MW frequency, 9.392 GHz; MW power, 2.0 mW.

Mössbauer Spectroscopy

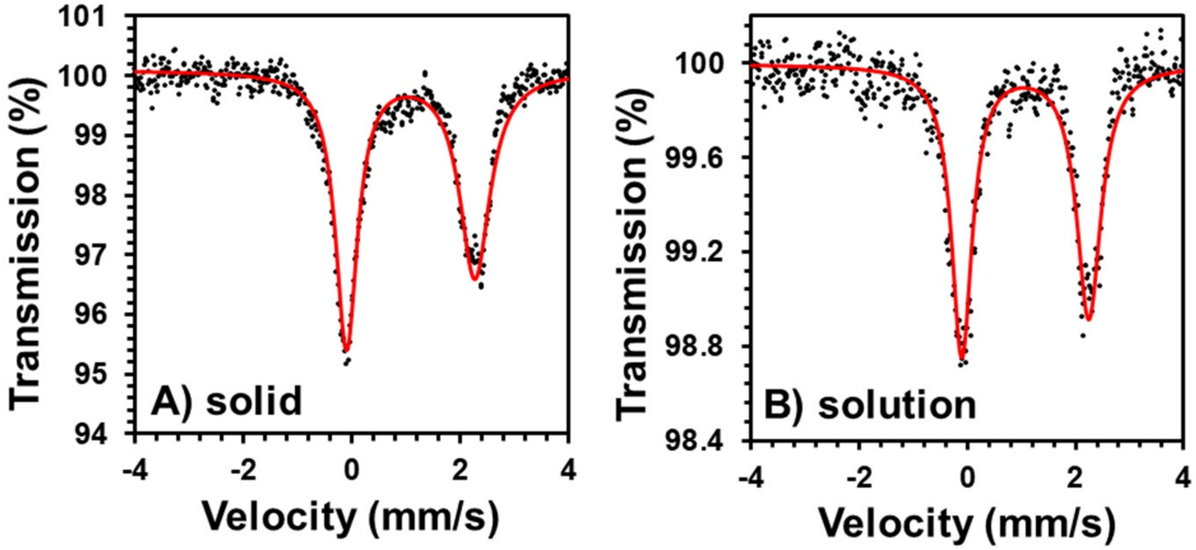

	δ (mm/s)	ΔE_Q (mm/s)
**1** (solid)	1.09(1)	2.37(1)
**1** (solution)	1.08(1)	2.35(2)

• **Raw data**
— **fit**

Figure S25. Mössbauer spectrum of **1** with fits. (A) Prepared as a powder. (B) Prepared as a frozen solution in toluene using ^{57}Fe enriched complex **1**. (Bottom, left) Isomer shift and quadrapole splitting parameters and (bottom, right) legend.

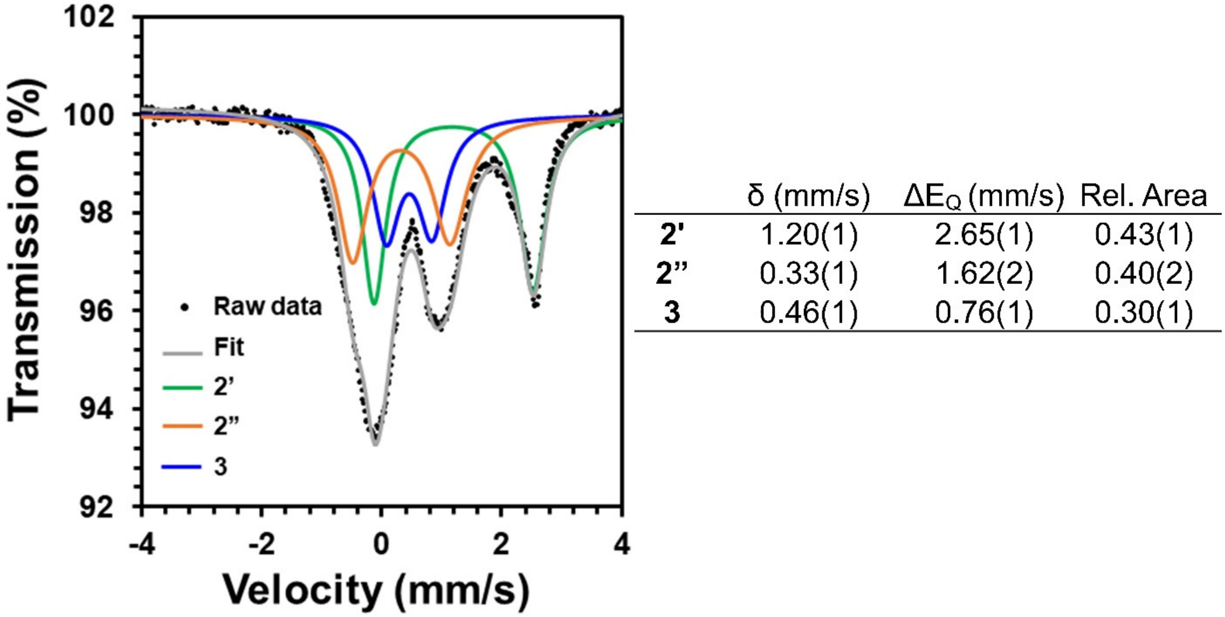

	δ (mm/s)	ΔE_Q (mm/s)	Rel. Area
**2'**	1.20(1)	2.65(1)	0.43(1)
**2"**	0.33(1)	1.62(2)	0.40(2)
**3**	0.46(1)	0.76(1)	0.30(1)

Figure S26. Mössbauer spectrum of **2** with fits. Parameters for all fits used in overall data fitting. Samples were prepared as a frozen solution in toluene using ^{57}Fe enriched complex **1** that were reacted with O_2 for 6 minutes at −60 °C.

As suggested by other data, the speciation of **2** is complicated. By UV-vis, growth of **3** can be seen while **2** still has features. Therefore, when fitting these data, one Fe center was constrained to the parameters for **3** in all aspects except relative area. The rest was fit using one or two additional Fe species, and the best overall fit was found to require two Fe species in addition to **3**. It is unclear due to the convoluted nature of this reaction exactly what the assignments for **2’** and **2”** are, but from these data it seems that one is an Fe(III) complex and the other is an Fe(II) complex. It seems probable that **2”** may be an Fe(III)-superoxo, but this cannot be definitively assigned from the current data. We suspect, based on the kinetic data presented in the paper, that this complexity arises from variable ligation of the Fe center on conversion from **1** to ultimately **3**.

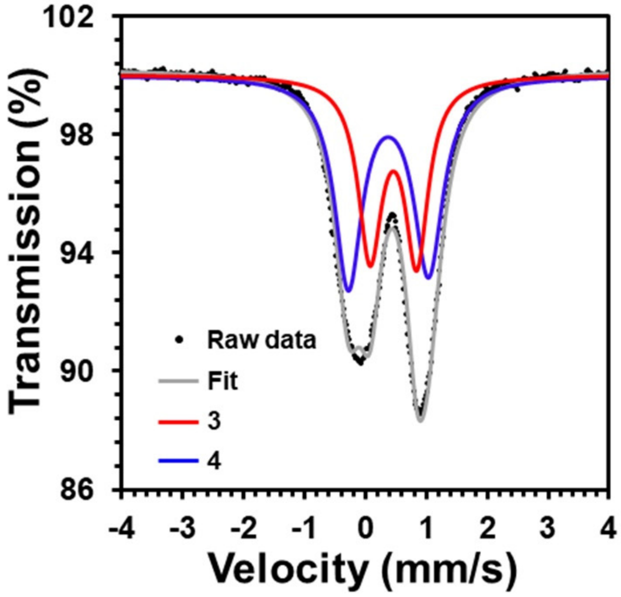

	δ (mm/s)	ΔE_Q (mm/s)	Rel. Area
**3**	0.46(1)	0.76(1)	0.47(1)
**4**	0.38(1)	1.62(2)	0.63(1)

Figure S27. Mössbauer spectrum of **4** as a mixture with **3** with fits. Parameters for all fits used in overall data fitting. Samples were prepared as a frozen solution in toluene using ^{57}Fe enriched complex **1** and allowed to evolve from **3** via warming.

X-ray Absorption Spectroscopy

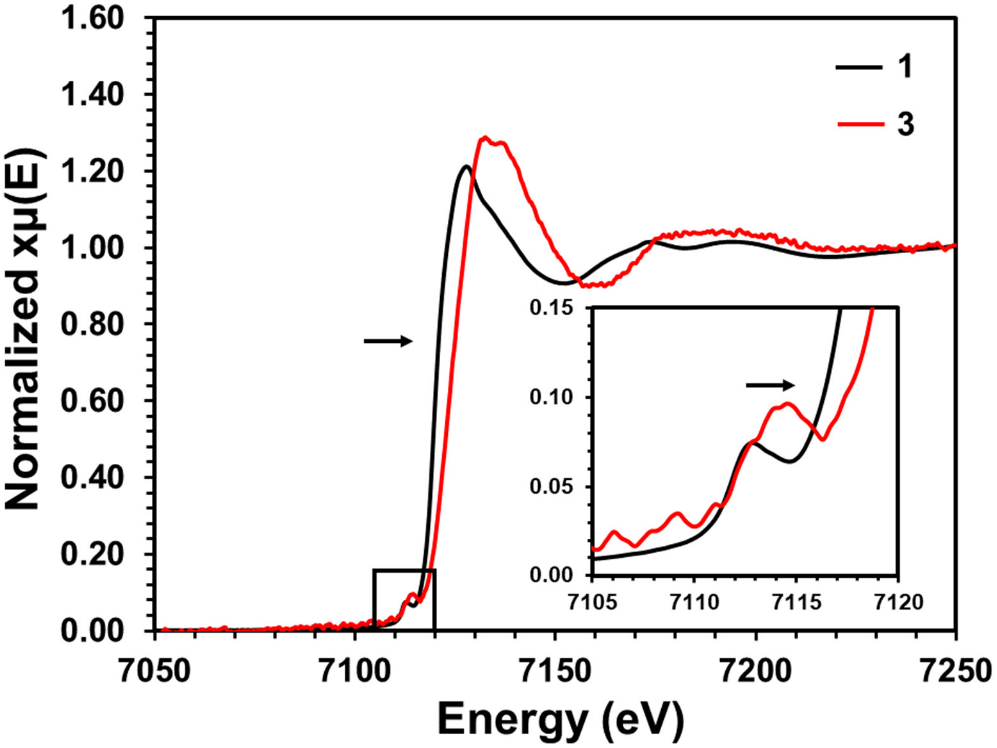

Figure S28. X-ray absorption spectra of **1** and **3** with K-edge inflection points of 7120 and 7124 eV respectively and pre-edge features at 7112 and 7114 eV respectively. **1** was collected as a solid powder at room temperature and **3** was collected a frozen solution in THF. Inset: Pre-edge features.

Single Crystal X-ray Diffraction

X-Ray Structure Determination.

The diffraction data were measured at 100 K on a Bruker D8 VENTURE with PHOTON 100 CMOS detector system equipped with a Mo-target micro-focus X-ray tube (λ = 0.71073 Å. Data reduction and integration were performed with the Bruker APEX3 software package (Bruker AXS, version 2015.5-2, 2015). Data were scaled and corrected for absorption effects using the multi-scan procedure as implemented in SADABS (Bruker AXS, version 2014/5, 2015, part of Bruker APEX3 software package). The structure was solved by the dual method implemented in SHELXT1 and refined by a full-matrix least-squares procedure using OLEX232 software package (XL refinement program version 2014/7[3,]3). Suitable crystals were mounted on a cryo-loop and transferred into the cold nitrogen stream of the Bruker D8 Venture diffractometer. C-H hydrogen atoms were generated by geometrical considerations, constrained to idealized geometries, and allowed to ride on their carrier atoms with an isotropic displacement parameter related to the equivalent displacement parameter of their carrier atoms.

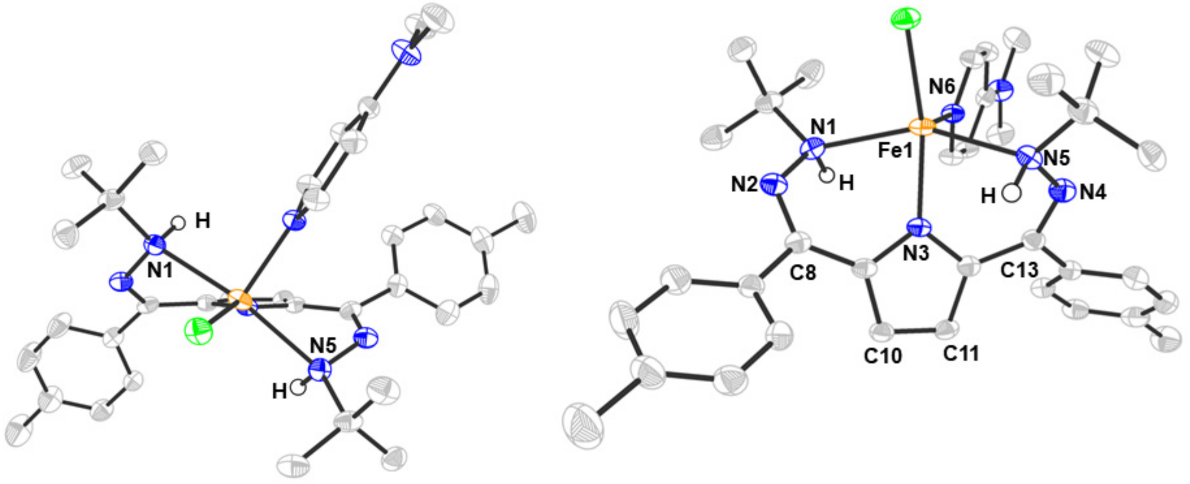

Figure S29. SXRD of **1** looking down the Cl-Fe-N3 bond (left) and looking down the Fe-N6 bond (right). Fe (orange), N (blue), C (gray), Cl (lime green), H (white). N-H protons were found in the difference map and refined. Selected bond lengths (Å). Fe1-Cl1: 2.2651(7), Fe1-N1: 2.399(2), Fe1-N3: 2.035(3), Fe1-N5: 2.362(2), Fe1-N6: 2.098(2), N1-N2: 1.416(3), N2-C8: 1.293(3), N4-N5: 1.410(3), N4-C13: 1.288(3), C10-C11: 1.390(4). Selected bond angles (°). N1-Fe1-N5: 152.93(8), N3-Fe1-Cl1: 153.00(1).

Table S1. SXRD of **1**.

Empirical formula	C39H50ClFeN6.5
Formula weight	701.15
Temperature/K	100(2)
Crystal system	triclinic
Space group	P-1
a/Å	14.4920(9)
b/Å	16.1734(10)
c/Å	18.9260(12)
α/°	107.242(2)
β/°	112.381(2)
γ/°	98.388(2)
Volume/Å3	3745.6(4)
Z	4
ρcalcg/cm3	1.243

μ/mm-1	0.510
F(000)	1490.0
Crystal size/mm3	0.1 × 0.1 × 0.02
Radiation	MoKα (λ = 0.71073)
2Θ range for data collection/°	4.082 to 56.844
Index ranges	-19 ≤ h ≤ 19, -21 ≤ k ≤ 21, -25 ≤ l ≤ 25
Reflections collected	127038
Independent reflections	18820 [Rint = 0.0664, Rsigma = 0.0569]
Data/restraints/parameters	18820/0/899
Goodness-of-fit on F2	1.020
Final R indexes [I>=2σ (I)]	R1 = 0.0579, wR2 = 0.1236
Final R indexes [all data]	R1 = 0.1001, wR2 = 0.1402
Largest diff. peak/hole / e Å-3	1.24/-0.73

Kinetic Measurements

To perform an Eyring analysis for the reaction of **2** to **3** under O_2, the intensity of the absorbance at 996 nm was monitored at a variety of temperatures. This feature was chosen because it was the only feature from the mixture of products in **2** that was not convoluted by features of **3** and because the feature is at its most intense when the transformation of **2** to **3** begins. Thus, the rate of the formation of **3** was determined by the rate of disappearance of the feature at 996 nm using an exponential fit to the data. The spectrum with the greatest intensity at 996 nm was not included in the fit as this should be a transition point between the growth of **2** and the conversion of **2** to **3**.

The rates calculated for the Eyring analysis of the reaction of **2** to **3** give a line of best fit with an R^2 value of 0.95:

$$y = -380.2x + 6.5208$$

Error in the y-intercept used to determine $\Delta S^{\ddagger}$ was determined by propagation of error in the line of best fit (i.e. error in the y-intercept for the line of best fit). This resulted in $\Delta S^{\ddagger} = -34 \pm 4.9$ cal/mol.

Error in the slope used to determine $\Delta H^{\ddagger}$ was determined by propagation of error in the line of best fit (i.e. error in the slope calculated for the line of best fit). This resulted in $\Delta H^{\ddagger} = 7.6 \pm 1.0$ kcal/mol.

Table S2. Calculated data for Eyring analysis of **2** to **3** at 996 nm.

1/T (1/K)	average ln(k/T)	standard deviation
0.004484305	−10.92381523	0.276300638

0.004587156	−11.04313989	0.239081968
0.004694836	−11.27880141	0.072683884
0.004807692	−11.82830624	0.07226749
0.004926108	−12.14165682	0.112592654

Table S3. Rates of the reaction of **2** to **3** at 996 nm.

Temperature (K)	rate	average rate (1/s)	1/T (1/K)	ln(k/T)	average ln(k/T)	standard deviation
223	0.00315	0.004135	0.004484	−11.16752	−10.92381523	0.276300638
	0.00519		0.004484	−10.66819		
	0.00318		0.004484	−11.15805		
	0.00502		0.004484	−10.7015		
218	0.00263	0.0035625	0.004587	−11.32527	−11.04313989	0.239081968
	0.00323		0.004587	−11.11977		
	0.00376		0.004587	−10.96783		
	0.00463		0.004587	−10.75969		
213	0.00266	0.002696667	0.004695	−11.29072	−11.27880141	0.072683884
	0.00291		0.004695	−11.20089		
	0.00252		0.004695	−11.34479		
208	0.00161	0.00152	0.004808	−11.76906	−11.82830624	0.07226749
	0.00155		0.004808	−11.80704		
	0.0014		0.004808	−11.90882		
203	0.00096	0.001088333	0.004926	−12.26178	−12.14165682	0.112592654
	0.0012		0.004878	−12.03864		
	0.001		0.004878	−12.22096		
	0.00127		0.004878	−11.98194		
	0.001		0.004878	−12.22096		
	0.0011		0.004878	−12.12565		

Table S4. Raw data for kinetic studies of **2** to **3** at −50 °C at 996 nm with a 0.7 mM solution in toluene.

50-1		50-2		50-3		50-4	
time (s)	intensity (a.u.)	time (s)	intensity (a.u.)	time (s)	intensity (a.u.)	time (s)	intensity (a.u.)
0	0.060367345	0	0.020802446	0	0.032047698	0	0.087381992
60	0.062211044	60	0.031027336	60	0.034477294	60	0.085973132
120	0.113161851	120	0.101999368	120	0.104371667	120	0.169699914
180	0.199385083	180	0.135781131	180	0.178863934	180	0.21977466
240	0.190726103	240	0.102634789	240	0.168836413	240	0.197595498
300	0.186178803	300	0.083724249	300	0.161091224	300	0.176583048
360	0.17455941	360	0.073013088	360	0.15334971	360	0.158349048
420	0.164192677	420	0.066659199	420	0.144541405	420	0.149174056

480	0.151188456	480	0.061674259	480	0.134548262	480	0.143297092
540	0.1379204	540	0.058417521	540	0.121438069	540	0.139520477
600	0.132764305	600	0.055152592	600	0.109927621	600	0.135400408
660	0.127506203	660	0.05351778	660	0.106053717	660	0.132970462
720	0.124907371	720	0.052205589	720	0.100733607	720	0.130464315
780	0.120676461	780	0.049477951	780	0.097191661	780	0.128917574
840	0.1188846	840	0.049794991	840	0.093564548	840	0.127232357
900	0.114258233	900	0.04762592	900	0.091678269	900	0.125760265
960	0.112768737	960	0.047421647	960	0.089230782	960	0.124608582
1020	0.109741714	1020	0.046109701	1020	0.085716787	1020	0.123576768
1080	0.108883698	1080	0.046301856	1080	0.084748855	1080	0.122435026
1140	0.10707703	1140	0.044758912	1140	0.083544204	1140	0.122276091
1200	0.106711538	1200	0.046551924	1200	0.079980764	1200	0.121261625
1260	0.105872208	1260	0.044855158	1260	0.079245849	1260	0.121857316
1320	0.104081662	1320	0.045775567	1320	0.077475427	1320	0.120396569
1380	0.102515702	1380	0.045847072	1380	0.076471059	1380	0.12093631
1440	0.10325185	1440	0.045609517	1440	0.076279856	1440	0.120314822

Table S5. Raw data for kinetic studies of **2** to **3** at −55 °C at 996 nm with a 0.7 mM solution in toluene.

55-1		55-2		55-3		55-4	
time (s)	intensity (a.u.)	time (s)	intensity (a.u.)	time (s)	intensity (a.u.)	time (s)	intensity (a.u.)
0	0.122669559	0	0.112180011	0	0.127406134	0	0.072407242
90	0.14435754	90	0.130778579	60	0.136138297	60	0.064009807
180	0.173077546	180	0.19059057	120	0.166797143	120	0.084685388
270	0.205497466	270	0.236579604	180	0.217680809	180	0.110170227
360	0.197338867	360	0.20609797	240	0.247902939	240	0.168534454
450	0.185537411	450	0.18769878	300	0.22528987	300	0.168637429
540	0.176982684	540	0.176902408	360	0.209027293	360	0.147780475
630	0.169602219	630	0.171150229	420	0.198413495	420	0.133048685
720	0.164921315	720	0.164745614	480	0.187713227	480	0.124566116
810	0.164476195	810	0.161871043	540	0.185163272	540	0.117082313
900	0.158206843	900	0.15875192	600	0.179594681	600	0.112228019
990	0.158121237	990	0.155154089	660	0.176002419	660	0.106899585
1080	0.153768033	1080	0.154890979	720	0.17369164	720	0.105371846
1170	0.153695476	1170	0.153696892	780	0.171026957	780	0.102241693
1260	0.153406123	1260	0.151096668	840	0.170433836	840	0.100205405
1350	0.150523995	1350	0.14973321	900	0.166018197	900	0.101120404
1440	0.150738243	1440	0.149520907	960	0.164814908	960	0.097214332
1530	0.148752478	1530	0.148332942	1020	0.163462749	1020	0.094760758
1620	0.149123888	1620	0.150092164	1080	0.162337816	1080	0.095142682
1710	0.148491818	1710	0.148394816	1140	0.162074698	1140	0.095799538
1800	0.147344385	1800	0.146959203	1200	0.161467575	1200	0.095221888

1890	0.147209969	1890	0.146785276	1260	0.159786348	1260	0.093901491
1980	0.146819099	1980	0.147852051	1320	0.158934911	1320	0.096048637
2070	0.147488335	2070	0.146538283	1380	0.15887744	1380	0.097279449
2160	0.148206703	2160	0.148408152	1440	0.159289601	1440	0.094076418

Table S6. Raw data for kinetic studies of **2** to **3** at −60 °C at 996 nm with a 0.7 mM solution in toluene.

60-1		60-2		60-3	
time (s)	intensity (a.u.)	time (s)	intensity (a.u.)	time (s)	intensity (a.u.)
0	0.038173918	0	0.031454992	0	0.055902154
90	0.046992605	90	0.035336517	90	0.067253089
180	0.070231125	180	0.067352131	180	0.082868718
270	0.102667692	270	0.106486734	270	0.113924596
360	0.142522428	360	0.139116698	360	0.155460343
450	0.126710999	450	0.117240443	450	0.154118526
540	0.111066177	540	0.098577616	540	0.138222161
630	0.098589549	630	0.089339646	630	0.12731475
720	0.090757405	720	0.078336858	720	0.117361032
810	0.08349553	810	0.07277705	810	0.111632827
900	0.077956815	900	0.067173524	900	0.105603637
990	0.074165846	990	0.063179638	990	0.097530933
1080	0.071851809	1080	0.05985622	1080	0.097088808
1170	0.06749191	1170	0.057483306	1170	0.092499769
1260	0.065421919	1260	0.055486793	1260	0.090881479
1350	0.063573005	1350	0.053968366	1350	0.088277244
1440	0.063388163	1440	0.052506304	1440	0.086896969
1530	0.060197258	1530	0.052325177	1530	0.086217788
1620	0.059666832	1620	0.050176005	1620	0.084564681
1710	0.060002091	1710	0.049525483	1710	0.084589641
1800	0.058807658				
1890	0.059104174				

Table S7. Raw data for kinetic studies of **2** to **3** at −65 °C at 996 nm with a 0.7 mM solution in toluene.

65-1		65-2		65-3	
time (s)	intensity (a.u.)	time (s)	intensity (a.u.)	time (s)	intensity (a.u.)
0	−0.008098454	0	−0.019902931	0	0.005023601
90	−0.015098372	90	−0.035465894	90	0.00214342
180	−0.010957906	180	−0.025711474	180	0.005707166
270	−0.003380267	270	−0.018687968	270	0.013750184
360	0.006992907	360	−0.004316518	360	0.025841984
450	0.023930803	450	0.012995775	450	0.041866409

540	0.052604096	540	0.040022507	540	0.072608848
630	0.115386475	630	0.09405642	630	0.114477221
720	0.162643302	720	0.142060011	720	0.134640819
810	0.153716057	810	0.140806006	810	0.138315322
900	0.139966979	900	0.137523927	900	0.13253262
990	0.123391266	990	0.129617235	990	0.120445362
1080	0.106579301	1080	0.12091972	1080	0.108559054
1170	0.092587229	1170	0.110100543	1170	0.099833806
1260	0.081080301	1260	0.093687865	1260	0.095569286
1350	0.070818262	1350	0.077647466	1350	0.086431166
1440	0.064181893	1440	0.069060898	1440	0.078830247
1530	0.055405251	1530	0.061644014	1530	0.074075219
1620	0.049619151	1620	0.052283168	1620	0.071682442
1710	0.045081861	1710	0.046700145	1710	0.066645755
1800	0.040373331	1800	0.04058199	1800	0.064743134
1890	0.03626537	1890	0.037992811	1890	0.059220324
1980	0.034681954	1980	0.034858346	1980	0.057504147
2070	0.0300981	2070	0.03178249	2070	0.05433391
2160	0.028723344	2160	0.026880324	2160	0.049219587

Table S8. Raw data for kinetic studies of **2** to **3** at −70 °C at 996 nm with a 0.7 mM solution in toluene.

70-1		70-2		70-3	
time (s)	intensity (a.u.)	time (s)	intensity (a.u.)	time (s)	intensity (a.u.)
0	−0.111086231	0	−0.047571219	0	−0.049016509
180	−0.113321874	180	−0.049448628	180	−0.045707223
360	−0.105994867	360	−0.0343815	360	−0.036968307
540	−0.101684529	540	−0.013193093	540	−0.022103876
720	−0.097664892	720	0.021146982	720	−0.003055319
900	−0.091458606	900	0.047809018	900	0.017737603
1080	−0.04406514	1080	0.029648363	1080	0.009835747
1260	−0.010567412	1260	0.017534631	1260	0.002669914
1440	0.026639319	1440	0.007276382	1440	−0.002435897
1620	0.011514591	1620	0.001952698	1620	−0.006123566
1800	−0.003168782	1800	−0.004119997	1800	−0.011442167
1980	−0.015450314	1980	−0.008556121	1980	−0.012940603
2160	−0.024974117	2160	−0.011210453	2160	−0.015530918
2340	−0.032652367	2340	−0.01567444	2340	−0.018462281
2520	−0.038747538	2520	−0.017610412	2520	−0.020184523
2700	−0.043972625	2700	−0.019530379	2700	−0.021930238
2880	−0.049626415	2880	−0.022292334	2880	−0.022829331
3060	−0.052732598	3060	−0.022157311	3060	−0.02359425
3240	−0.056571639	3240	−0.024912142	3240	−0.023952484

3420	−0.059044883	3420	−0.024370416	3420	−0.025226962
3600	−0.062663564	3600	−0.024298329	3600	−0.026712345
3780	−0.064041643	3780	−0.024988319	3780	−0.028086301
3960	−0.066978446	3960	−0.027191158	3960	−0.026466567
4140	−0.067155758	4140	−0.027855564	4140	−0.028905362
4320	−0.069596353	4320	−0.026368292	4320	−0.025987672

Table S8. Raw data for kinetic studies of **2** to **3** at −70 °C at 996 nm with a 0.7 mM solution in toluene. (continued)

70-4		70-5		70-6	
time (s)	intensity (a.u.)	time (s)	intensity (a.u.)	time (s)	intensity (a.u.)
0	0.00526947	0	0.074129176	0	0.096511924
180	0.003181292	180	0.075904449	180	0.097017546
360	0.007936812	360	0.084922389	360	0.109875984
540	0.01262428	540	0.095134391	540	0.124227115
720	0.022402183	720	0.108311649	720	0.138357787
900	0.034274308	900	0.130299284	900	0.166562038
1080	0.056161811	1080	0.163563658	1080	0.195176288
1260	0.097701934	1260	0.166300529	1260	0.183394631
1440	0.107968484	1440	0.150496021	1440	0.16980488
1620	0.084779464	1620	0.138973443	1620	0.160256923
1800	0.069873362	1800	0.131012377	1800	0.15227035
1980	0.060384784	1980	0.125029533	1980	0.147986292
2160	0.051190898	2160	0.118193783	2160	0.14327852
2340	0.043213012	2340	0.111983961	2340	0.139314339
2520	0.037983119	2520	0.108816732	2520	0.136449256
2700	0.034515965	2700	0.103255842	2700	0.134355937
2880	0.031846629	2880	0.101862274	2880	0.132033334
3060	0.027735758	3060	0.10075897	3060	0.130166687
3240	0.026353908	3240	0.098254518	3240	0.128078842
3420	0.023577265	3420	0.095069223	3420	0.126719427
3600	0.022623359			3600	0.124583773
3780	0.02173668			3780	0.123363445
3960	0.020826048			3960	0.124855821
4140	0.020161671			4140	0.122264148
4320	0.018557667			4320	0.121692849
				4500	0.122958441
				4680	0.121673643
				4860	0.121835691
				5040	0.122779357
				5220	0.119718142

Table S9. Raw data for kinetic studies of **2** to **3** at −65 °C at 996 nm with a 0.7 mM solution in toluene using deuterated ligand.

65-1, 1-D_2		65-2, **1-D_2**	
time (s)	intensity (a.u.)	time (s)	intensity (a.u.)
1260	−0.0503609	840	−0.0347548
1440	−0.0577697	960	−0.0437469
1620	−0.062108	1080	−0.0518273
1800	−0.066227	1200	−0.0558909
1980	−0.0693164	1320	−0.061047
2160	−0.0693303	1440	−0.0643078
2340	−0.0723233	1560	−0.0687214
2520	−0.0730058	1680	−0.0714665
2700	−0.0740769	1800	−0.0722919
2880	−0.0743885	1920	−0.0727373
3060	−0.0749013	2040	−0.0742963
3240	−0.0761959	2160	−0.0758303
3420	−0.0775325	2280	−0.0761745
		2400	−0.0773556
		2520	−0.078203
		2640	−0.0794579
		2760	−0.0795198
		2880	−0.080817
		3000	−0.0804472
		3120	−0.0826779
		3240	−0.0799076
		3360	−0.0820425
		3480	−0.0825121

Table S10. Raw data for kinetic studies of **2** to **3** at −50 °C at 996 nm with a 0.7 mM solution in toluene using deuterated ligand.

50-1, **1-D_2**		50-2, **1-D_2**		50-3, **1-D_2**	
time (s)	intensity (a.u.)	time (s)	intensity (a.u.)	time (s)	intensity (a.u.)
300	0.23798722	300	−0.0540242	300	−0.0346024
360	0.23058259	360	−0.0631177	360	−0.0418951
420	0.22446459	420	−0.0671787	420	−0.0455482
480	0.22222824	480	−0.0712744	480	−0.0505973
540	0.21782386	540	−0.0725571	540	−0.0531032
600	0.21778324	600	−0.0762909	600	−0.0543047
660	0.21643005	660	−0.076216	660	−0.0567573
720	0.21532544	720	−0.0771862	720	−0.0555895
780	0.2146879	780	−0.0782558	780	−0.0567197
840	0.21402892	840	−0.0773912	840	−0.0577657

900	0.21439524	900	−0.0798387	900	−0.0581477
960	0.21347061	960	−0.0784421	960	−0.0591111
1020	0.2138512	1020	−0.0787845	1020	−0.0598603
1080	0.21250464	1080	−0.0798932	1080	−0.0600132
1140	0.21360223	1140	−0.0790192	1140	−0.0611053
1200	0.21291062	1200	−0.0793828	1200	−0.0595687
1260	0.21332716	1260	−0.0811807	1260	−0.0599882
1320	0.2144795	1320	−0.079292	1320	−0.0602748
1380	0.21430489	1380	−0.0808056	1380	−0.0601482
1440	0.21424468	1440	−0.080247	1440	−0.0616091
1500	0.2127725	1500	−0.0807857	1500	−0.0615909
1560	0.21353079	1560	−0.0795968	1560	−0.0616691
1620	0.21397466	1620	−0.0799228	1620	−0.0621219
1680	0.21237022	1680	−0.0807522	1680	−0.0614901
1740	0.21290116	1740	−0.080199	1740	−0.0614459

Table S11. Rates of reaction for kinetic isotope effect calculation.

	**1-H₂** rates (s^{-1})		**1-D₂** rates (s^{-1})	
	−50 °C	−65 °C	−50 °C	−65 °C
	0.003150	0.001610	0.006200	0.001600
	0.005190	0.001550	0.004960	0.001630
	0.003180	0.001400	0.003950	
	0.005020			
average:	0.004135	0.001520	0.005037	0.001615
*error:	0.000972	0.000088	0.000920	0.000015

*Error was calculated using standard deviation when 3 or more measurements were taken. Error was calculated using average deviation when only 2 measurements were taken.

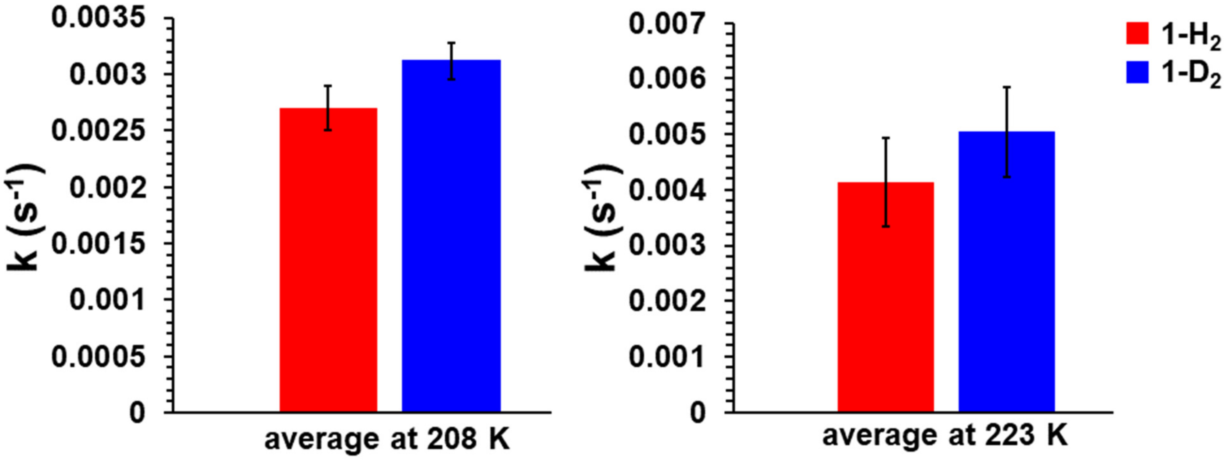

Figure S30. Rates of reaction for kinetic isotope effect experiment at various temperatures.

This set of experiments gives KIE's of 0.82±0.24 at −50 °C and 0.94±0.06 at −65 °C, both of which are within error of 1. This data is therefore either consistent with a small inverse KIE or a small normal KIE. One explanation of a KIE near 1 is that there is not an H-atom or proton transfer in the rate determining step. This might be expected if there was a rate determining ligand association/dissociation, a scenario which is supported by the measured entropy of activation. Alternatively, an inverse KIE may be explained by a stronger O–H bond in the transition state, although an equilibrium isotope effect (EIE) is more likely in this context.

Density Functional Theory (DFT)

Geometry Optimizations

Geometry optimization calculations were performed with ORCA4 software suite using density functional theory (DFT). Geometries were fully optimized starting from coordinates generated from finalized cifs of the compound crystal structures. The B3P functional was used with a basis set of def2-SVP on H, def2-TZVPP on Fe, N, and P, and def2-TZVP on C atoms. The resulting structures were confirmed to be minima on the potential energy surface by frequency calculations using ORCA. Frequency calculations were also conducted using the B3P functional and previously listed basis sets for each atom type.

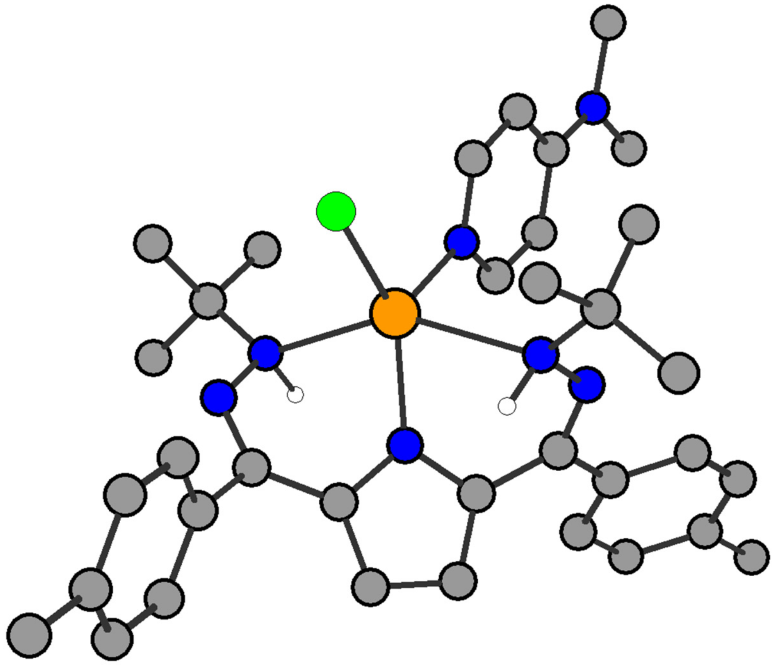

Figure S31. Calculated structure of **1**. All C–H hydrogen atoms have been removed for clarity.

Table S12. Calculated coordinates of **1**.

N	−1.259456	−0.602763	−0.131906
C	−2.384772	−1.379178	−0.136305
C	−2.105695	−2.608823	−0.733955
C	−0.766892	−2.563555	−1.140810
C	−0.265614	−1.323849	−0.742116
H	−0.229242	−3.331987	−1.676225
H	−2.803262	−3.422062	−0.879058
Fe	−1.099062	1.398691	0.284837
C	1.041363	−0.705726	−0.970905
N	1.602045	0.159005	−0.204046
N	0.971178	0.527978	0.984936
C	1.956638	1.109920	1.946195
C	1.164563	1.663467	3.123025
H	0.504416	2.473153	2.803616
H	1.850978	2.055136	3.878795
H	0.553792	0.886296	3.597826
C	2.731828	2.228982	1.265187
H	2.049493	2.962341	0.829596
H	3.361680	1.835809	0.466024
H	3.368643	2.727452	2.003434
C	2.911119	0.014560	2.419771
H	2.375079	−0.764207	2.975971
H	3.677549	0.433049	3.079664
H	3.408846	−0.448530	1.563441
N	−3.322643	1.096808	−0.855564
N	−4.069647	0.349399	0.036064

C	−3.654129	−0.830147	0.354670
C	−4.526378	−1.618394	1.254250
C	−1.737295	−0.928301	−2.269753
C	−5.850127	−1.227689	1.479804
C	−6.673559	−1.958156	2.317206
C	−6.221136	−3.114228	2.953443
C	−4.900348	−3.498400	2.736230
C	−4.064504	−2.763730	1.906865
H	−6.222672	−0.341643	0.978907
H	−7.699231	−1.633368	2.474782
H	−4.515127	−4.392255	3.220336
H	−3.038833	−3.084208	1.756825
C	−7.129976	−3.911496	3.838318
H	−8.169668	−3.595568	3.721039
H	−6.859715	−3.790174	4.894812
H	−7.071994	−4.980018	3.605891
C	1.911514	0.174177	−3.108542
C	2.520806	0.033864	−4.343504
C	2.986840	−1.204379	−4.783766
C	2.828941	−2.296071	−3.933693
C	2.210149	−2.165090	−2.695463
H	1.554344	1.144776	−2.778826
H	2.634976	0.906166	−4.982516
H	2.114573	−3.034241	−2.052018
H	3.197966	−3.270135	−4.243155
Cl	−0.241042	3.240868	−0.680213
C	−4.167806	2.045113	−1.632478
C	−4.750582	3.082377	−0.685628
H	−5.348478	2.600055	0.091288
H	−5.390869	3.774045	−1.242143
H	−3.954326	3.659529	−0.208759
C	−3.254550	2.701450	−2.657186
H	−2.452830	3.264663	−2.174738
H	−3.838154	3.379582	−3.287114
H	−2.796945	1.952096	−3.314366
C	−5.282710	1.284621	−2.349430
H	−5.929194	0.769929	−1.633525
H	−4.865452	0.535865	−3.032349
H	−5.894945	1.974101	−2.939678
N	−2.108422	2.096353	2.031403
C	−3.386329	1.752473	4.032304
C	−3.581499	3.136244	4.211692
C	−2.990655	3.970128	3.241012
C	−2.288231	3.415306	2.196232
C	−2.660307	1.298027	2.954786
H	0.529637	−0.289158	1.420642
C	3.632246	−1.339583	−6.133435
H	2.933889	−1.059279	−6.929489
H	3.961957	−2.364614	−6.318693
H	4.503091	−0.682184	−6.224199
H	−2.516086	0.230259	2.818607

H	−3.800423	1.026367	4.718859
H	−2.823503	0.487387	−1.511443
H	−1.832917	4.048838	1.439966
H	−3.085145	5.047048	3.285529
N	−4.293237	3.634227	5.256511
C	−4.971568	2.733445	6.161525
C	−4.516484	5.058289	5.369239
H	−5.098580	5.453737	4.524901
H	−3.571090	5.608637	5.422380
H	−5.073188	5.260090	6.284061
H	−5.678112	2.080853	5.631080
H	−5.531294	3.320340	6.889372
H	−4.264167	2.099115	6.709754

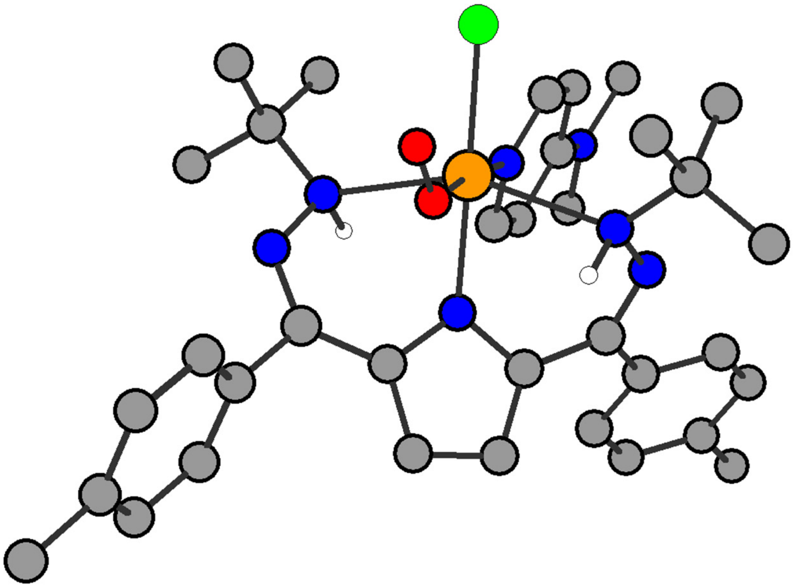

Figure S32. Calculated structure of a high spin Fe(III)(^{tBu,Tol}DHP-H_2)(DMAP)(Cl)(OO$^•$). All C–H hydrogen atoms have been removed for clarity.

Table S13. Calculated coordinates of high spin Fe(III)(^{tBu,Tol}DHP-H_2)(DMAP)(Cl)(OO$^•$).

N	−1.506615	−0.064433	−0.423159
C	−2.622683	−0.849043	−0.513275
C	−2.280722	−2.117676	−0.984665
C	−0.904286	−2.103350	−1.206514
C	−0.445550	−0.836151	−0.825920
H	−0.305121	−2.908636	−1.602879
H	−2.963301	−2.937364	−1.160808
Fe	−1.476139	2.045426	−0.346653
C	0.913841	−0.326718	−0.886505
N	1.388341	0.722267	−0.312793
N	0.568132	1.460491	0.558340

C	1.440411	2.369466	1.381719
C	0.555233	3.085374	2.389109
H	−0.176119	3.723918	1.890518
H	1.181660	3.713052	3.030140
H	0.026349	2.374738	3.034969
C	2.116484	3.367814	0.455397
H	1.376792	3.968307	−0.080702
H	2.745673	2.848294	−0.270159
H	2.746203	4.040496	1.046475
C	2.476721	1.536307	2.131482
H	1.985976	0.822493	2.804399
H	3.108938	2.190354	2.740570
H	3.110766	0.979694	1.437774
N	−3.594987	1.564604	−1.379225
N	−4.377178	0.822568	−0.501435
C	−3.938422	−0.327560	−0.127054
C	−4.805673	−1.097992	0.795784
C	1.905369	−1.064509	−1.721382
C	−6.129948	−0.704208	1.021921
C	−6.922962	−1.369117	1.937576
C	−6.440095	−2.465477	2.658137
C	−5.127987	−2.863293	2.423814
C	−4.322008	−2.192213	1.513063
H	−6.519484	0.146247	0.474205
H	−7.943814	−1.032200	2.100981
H	−4.719240	−3.709791	2.969919
H	−3.296568	−2.514328	1.368173
C	−7.318677	−3.198788	3.627932
H	−7.930131	−2.508174	4.218142
H	−6.728530	−3.811176	4.315397
H	−8.007001	−3.868211	3.097515
C	2.500000	−0.447712	−2.818997
C	3.437820	−1.124799	−3.586275
C	3.819828	−2.428357	−3.278167
C	3.236371	−3.031738	−2.164369
C	2.293838	−2.363400	−1.398219
H	2.219064	0.569320	−3.072029
H	3.886133	−0.626340	−4.441854
H	1.869421	−2.849233	−0.525030
H	3.534397	−4.038145	−1.881717
O	−0.660087	1.911858	−2.212541
C	−4.439427	2.517090	−2.168043
C	−5.050415	3.537581	−1.221266
H	−5.658396	3.041696	−0.461275
H	−5.688199	4.223916	−1.787055
H	−4.266695	4.121388	−0.732019
C	−3.529907	3.197651	−3.178927
H	−2.766435	3.801482	−2.685401
H	−4.129467	3.852270	−3.818528
H	−3.035223	2.466235	−3.828094
C	−5.526916	1.739315	−2.905915

H	−6.177783	1.209889	−2.205414
H	−5.084455	1.003618	−3.587564
H	−6.138511	2.423814	−3.502166
N	−2.451510	2.014251	1.642802
C	−3.144952	0.824832	3.612662
C	−3.811978	1.952881	4.127118
C	−3.758920	3.111932	3.328297
C	−3.086270	3.091125	2.128288
N	−4.464361	1.924628	5.316266
H	−4.244033	4.030587	3.630033
C	−2.497061	0.911304	2.402574
H	0.107365	0.809141	1.204040
C	4.802858	−3.174301	−4.131206
H	5.421842	−2.491456	−4.719790
H	4.281519	−3.836201	−4.833592
H	5.463590	−3.799030	−3.521994
Cl	−1.396147	4.328231	−0.449509
H	−3.034714	3.983336	1.513516
H	−3.140998	−0.122793	4.134468
H	−3.117976	0.939644	−2.039647
C	−5.181530	3.093507	5.774994
C	−4.523687	0.698674	6.081596
H	−5.985605	3.377093	5.084140
H	−4.512414	3.953707	5.893808
H	−5.629728	2.876917	6.743943
H	−5.047751	−0.097230	5.536711
H	−5.061280	0.887250	7.010268
H	−3.521395	0.337412	6.338501
H	−1.997247	0.036508	1.999963
O	−0.217703	2.760876	−2.985738

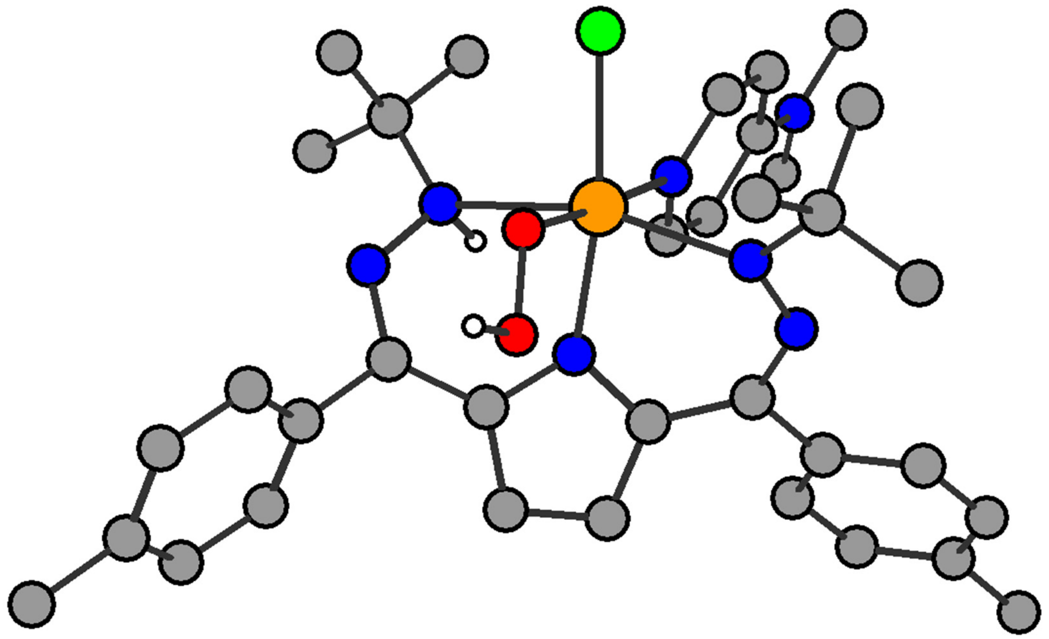

Figure S33. Calculated structure of a high spin Fe(III)(^{tBu,Tol}DHP-H$^{\bullet}$)(DMAP)(Cl)(OOH), **3**. All C–H hydrogen atoms have been removed for clarity.

Table S14. Calculated coordinates of high spin Fe(III)(^{tBu,Tol}DHP-H$^{\bullet}$)(DMAP)(Cl)(OOH), **3**.

N	−1.506731	−0.144120	−0.273740
C	−2.585739	−0.979164	−0.205860
C	−2.169603	−2.312761	−0.447304
C	−0.816580	−2.263900	−0.702969
C	−0.422803	−0.913507	−0.566294
H	−0.182490	−3.090694	−0.980786
H	−2.814691	−3.177948	−0.494272
Fe	−1.659687	1.941493	−0.448400
C	0.897780	−0.335784	−0.776176
N	1.315025	0.783143	−0.301919
N	0.521166	1.507932	0.577135
C	1.385015	2.469534	1.341771
C	0.509810	3.179694	2.362167
H	−0.250487	3.789914	1.873172
H	1.137775	3.831662	2.976897
H	0.018606	2.464053	3.032388
C	1.994976	3.463681	0.365263
H	1.215249	4.006916	−0.173288
H	2.629430	2.945829	−0.356988
H	2.607982	4.183248	0.917365
C	2.477760	1.693126	2.074100
H	2.041891	0.980021	2.784481
H	3.110968	2.384445	2.639295
H	3.103544	1.140808	1.368816
N	−3.729213	1.582251	−1.022828

N	−4.418194	0.606842	−0.570568
C	−3.928466	−0.530826	−0.065230
C	−4.948139	−1.413013	0.519772
C	1.871351	−1.031637	−1.664251
C	−6.293953	−1.294648	0.152397
C	−7.261728	−2.103302	0.720668
C	−6.934664	−3.066097	1.675670
C	−5.595036	−3.179908	2.048957
C	−4.620351	−2.372960	1.488271
H	−6.564757	−0.563779	−0.599892
H	−8.295873	−1.994267	0.404471
H	−5.310524	−3.913282	2.799756
H	−3.590350	−2.473233	1.813412
C	−7.978234	−3.959225	2.274173
H	−8.974082	−3.712771	1.899607
H	−7.995190	−3.871266	3.366452
H	−7.777463	−5.009897	2.033115
C	2.416888	−0.354480	−2.754183
C	3.336715	−0.972954	−3.588100
C	3.750772	−2.284059	−3.361653
C	3.226244	−2.946578	−2.253148
C	2.301324	−2.335403	−1.419699
H	2.117559	0.671911	−2.935126
H	3.743243	−0.424766	−4.434300
H	1.937735	−2.869506	−0.547891
H	3.555762	−3.957783	−2.028406
O	−0.849292	1.968811	−2.153986
C	−4.570079	2.513902	−1.855938
C	−4.906562	3.748898	−1.017631
H	−5.449045	3.458377	−0.112584
H	−5.551588	4.411745	−1.603213
H	−4.006442	4.300407	−0.743291
C	−3.748039	2.899805	−3.082397
H	−2.799930	3.359853	−2.801209
H	−4.318017	3.613366	−3.684979
H	−3.535667	2.019521	−3.695900
C	−5.874536	1.857858	−2.301983
H	−6.551509	1.691471	−1.461759
H	−5.695633	0.901002	−2.799764
H	−6.369300	2.527008	−3.012365
N	−2.505517	1.945891	1.732558
C	−2.978389	0.923592	3.856991
C	−3.575099	2.099270	4.352725
C	−3.626900	3.183295	3.456109
C	−3.091885	3.058363	2.195785
C	−2.475830	0.904482	2.573821
H	0.066180	0.877673	1.245037
C	4.713794	−2.967436	−4.286438
H	5.291282	−2.245913	−4.871042
H	4.180284	−3.613772	−4.994305
H	5.414715	−3.599076	−3.731747

Cl	−1.474165	4.246297	−0.386704
H	−2.021502	−0.003713	2.189898
H	−3.102683	3.900870	1.512947
H	−4.074582	4.127910	3.734490
H	−2.907828	0.026555	4.458397
N	−4.068812	2.190521	5.613544
C	−4.688367	3.420106	6.056816
C	−3.952548	1.074628	6.524594
H	−5.516634	3.711012	5.400679
H	−3.970503	4.250332	6.091327
H	−5.090780	3.274386	7.058401
H	−2.904570	0.797937	6.695783
H	−4.483423	0.189864	6.152094
H	−4.388928	1.353363	7.483156
O	−0.935083	0.798721	−2.939653
H	−0.469141	1.084102	−3.741083

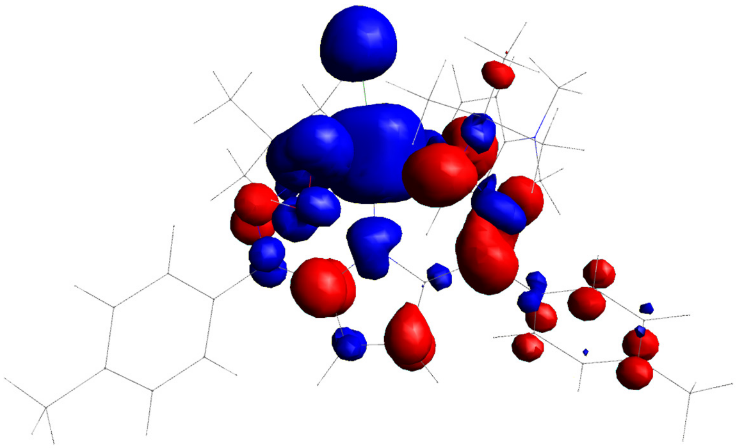

Figure S34. Spin density plot of **3** at an iso value of 0.003.

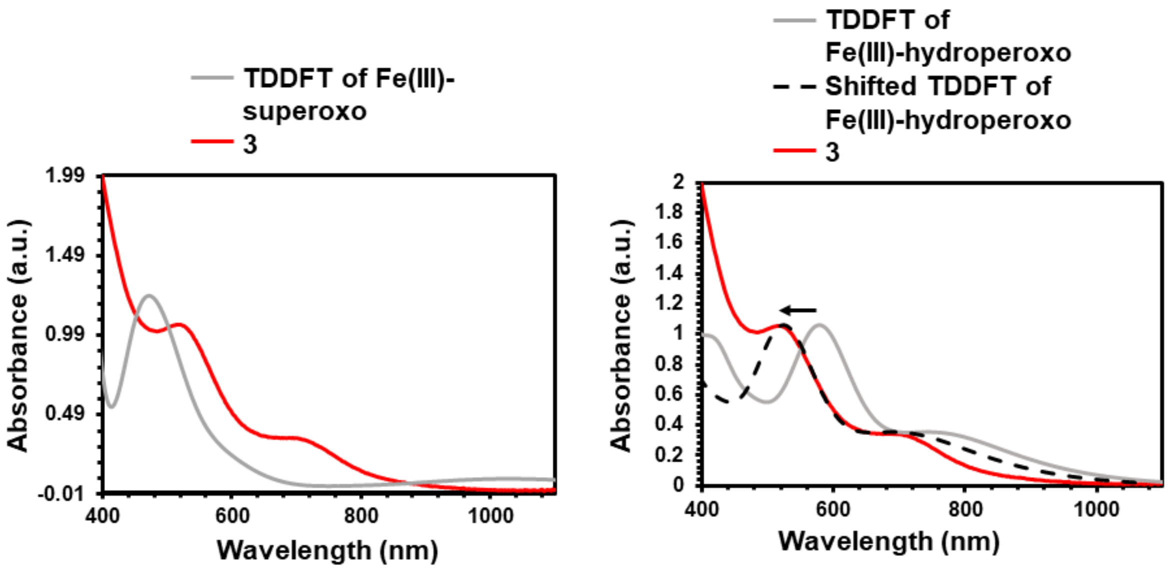

Figure S35. TDDFT of Fe(III)(^{tBu,Tol}DHP-H$_2$)(DMAP)(Cl)(OO$^\bullet$) (left) and Fe(III)(^{tBu,Tol}DHP-H$^\bullet$)(DMAP)(Cl)(OOH) (right), as compared to **3**. Note that TD-DFT typically underestimates the energies of transitions and a blue-shift to match experimental data of 50-75 nm is common in the related Ni-DHP complexes.[2]

State 4: 795 nm

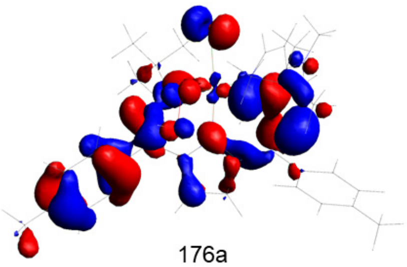

176a

→

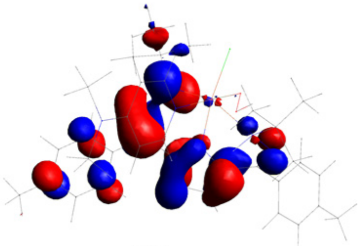

179a

15%

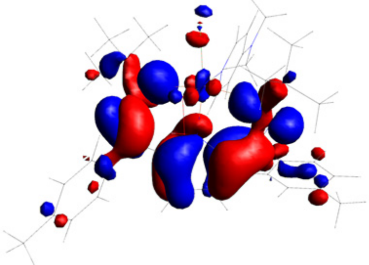

178a

→

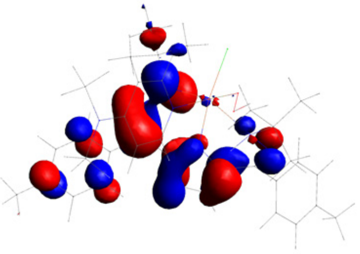

179a

65%

State 5: 714 nm

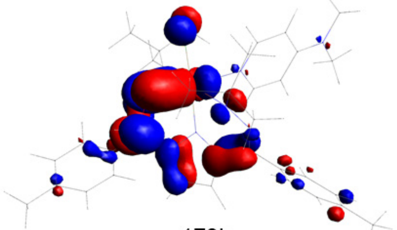

173b

→

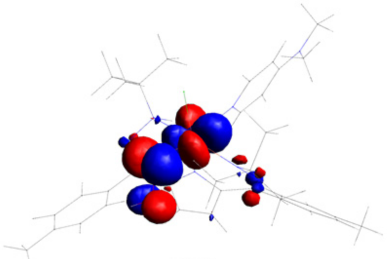

175b

11%

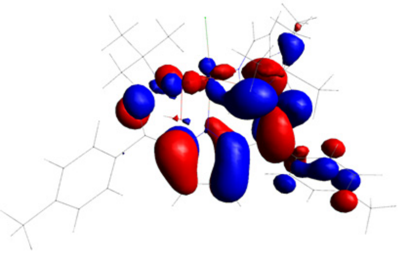

174b

→

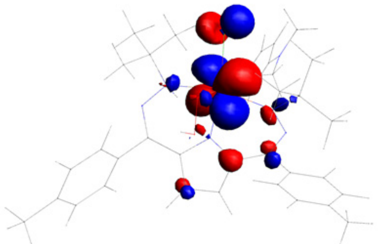

176b

70%

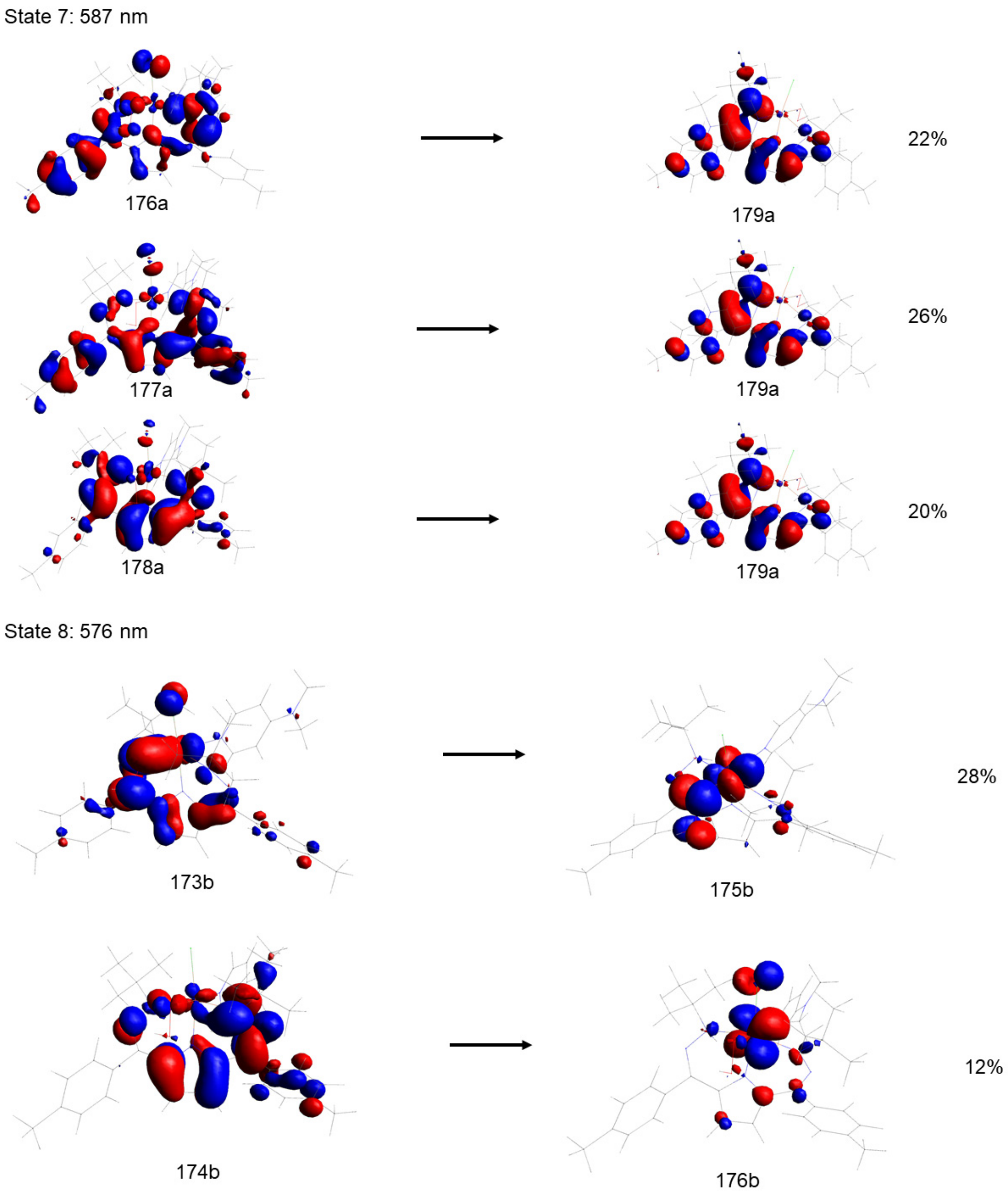

Figure S36. Molecular orbitals involved in transitions contributing to states involved in the major features by UV-vis spectroscopy in 3 as calculated by TDDFT. Percentages to the right of the transition show the contribution of that transition to each calculated state. Only contributions above 10% are listed.

Table S15. Calculated vs. experimental values.

	δ[a]	ΔE_Q[a]	O^{16}–O^{16} [b]	O^{18}–O^{18}	O^{16}–H	O^{16}–D	N–H	N–D
experimental value	0.460(2)	0.765(3)	882	840	3420	2546	3230	2375-2404
theoretical value	0.47	−0.85	969	-	3707	-	3354	-
Exp./Theor. Ratio	-	-	0.91	-	0.92	-	0.96	-

[a] units of mm/s
[b] units of cm^{-1}

The theoretical stretching frequencies were determined from the B3P DFT calculation for the structure of **3**. Stretching frequencies calculated by DFT need to be scaled to reflect experimental data. In this case, the scalings for the DFT calculated frequencies are 0.91, 0.92, and 0.96 for the O–O, O–H, and N–H stretches respectively. While the scaling for the N–H stretch is higher, this scaling (0.96) is consistent with that found for the N-H stretch in **1**.

Gas Chromatography-Mass Spectrometry (GC-MS)

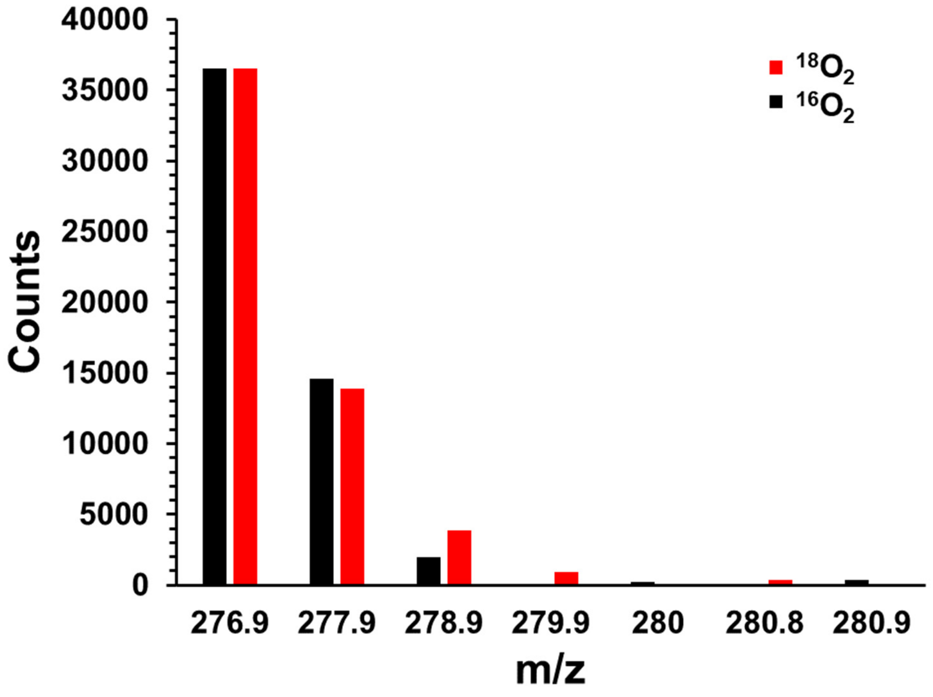

Figure S37. Mass spectrometry of the reaction of **1** with $^{16}O_2$ or $^{18}O_2$ and PPh_3 to form $OPPh_3$.

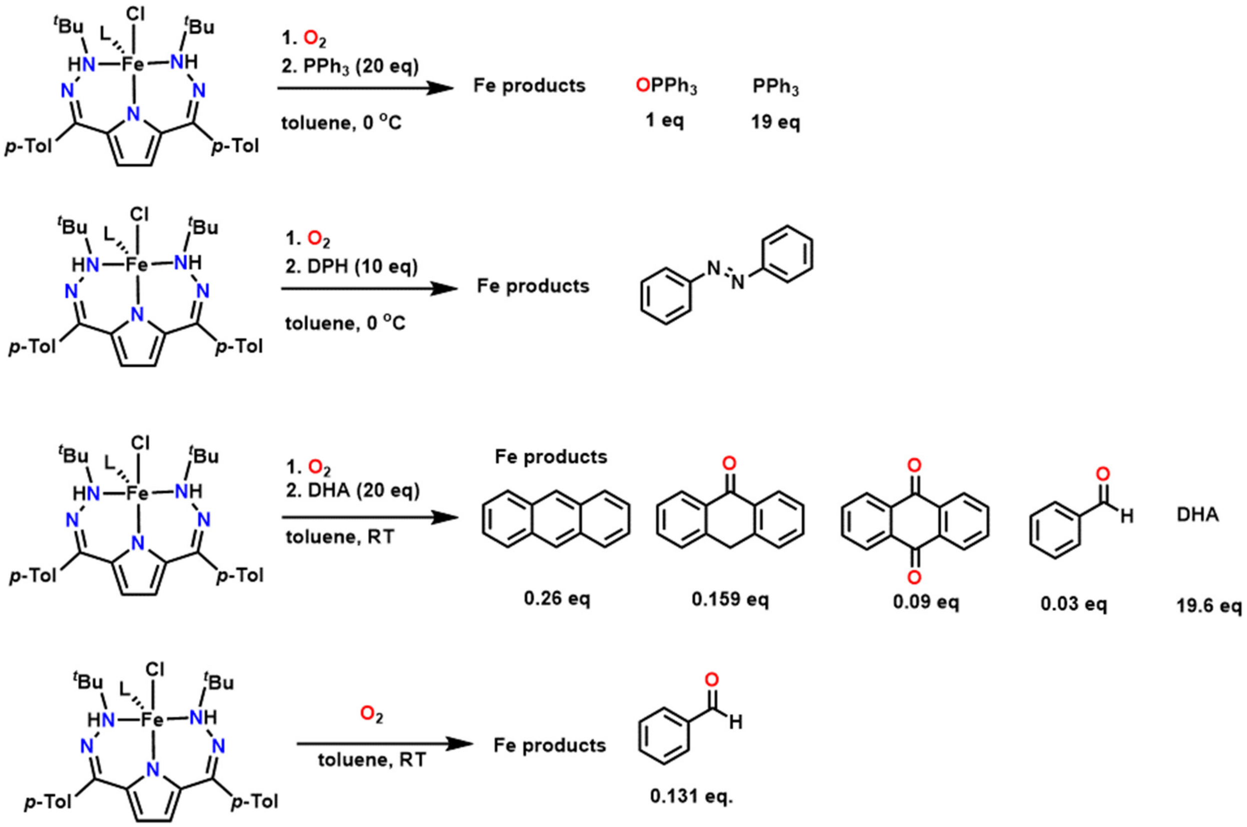

Figure S38. Conditions and resulting products observed by GC-MS. Yields are relative to 1 equivalent of **1.**

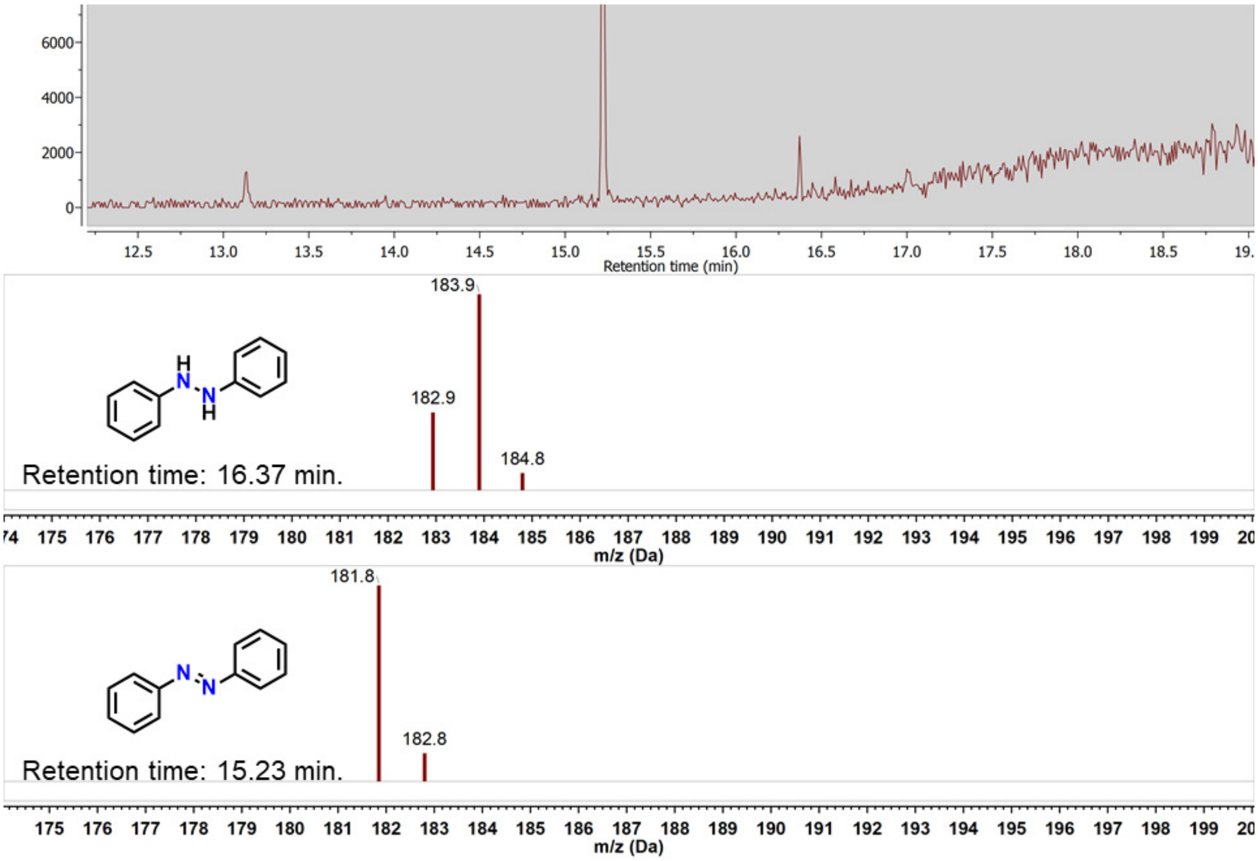

Figure S39. GC-MS the resulting product mixture of the reaction of **1** with excess O_2 and 10 equivalents of diphenylhydrazine (DPH) at 0 °C in toluene.

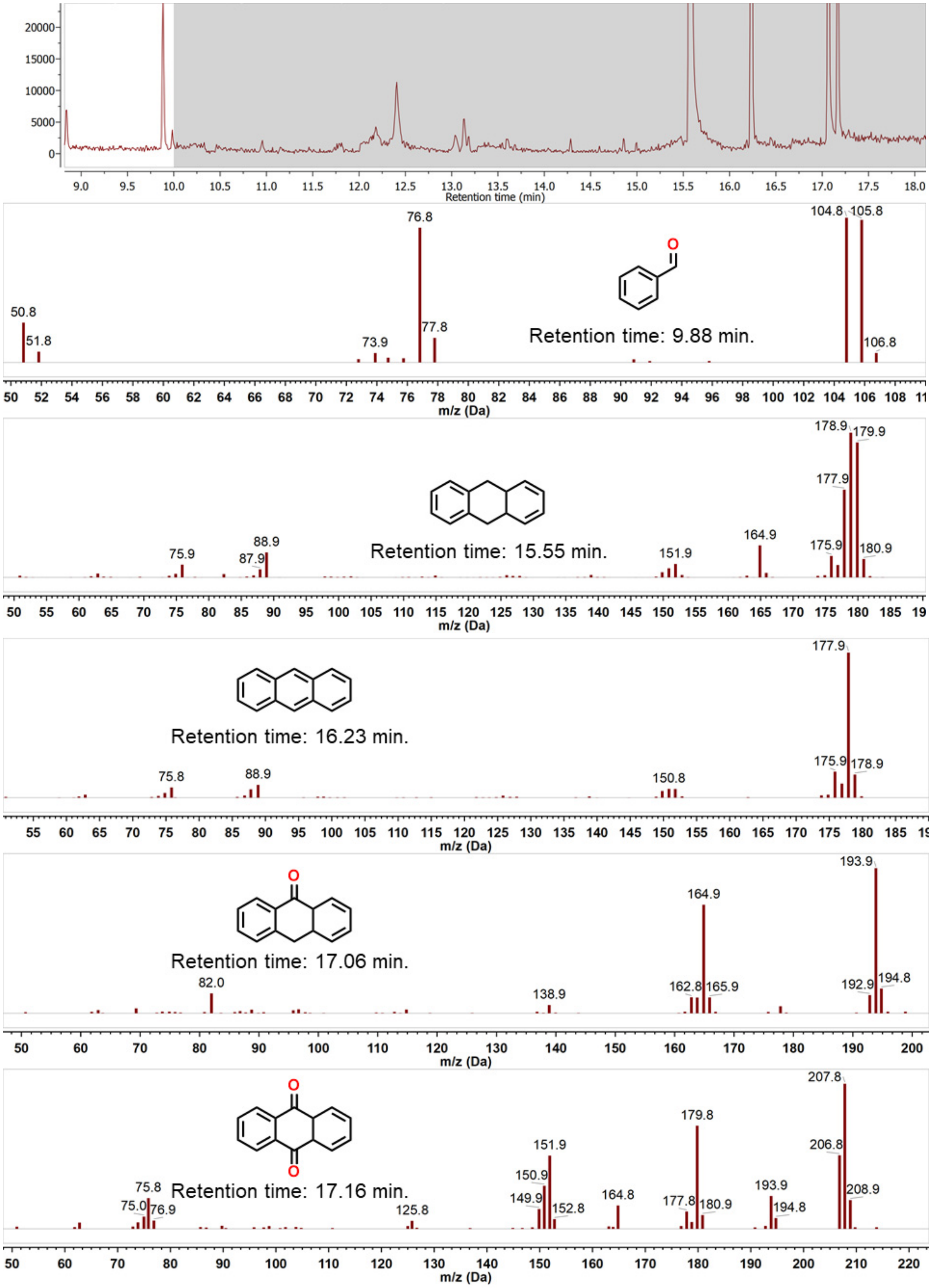

Figure S40. GC-MS the resulting product mixture of the reaction of **1** with excess O_2 and 20 equivalents of dihydroanthacene (DHA) at room temperature in toluene.

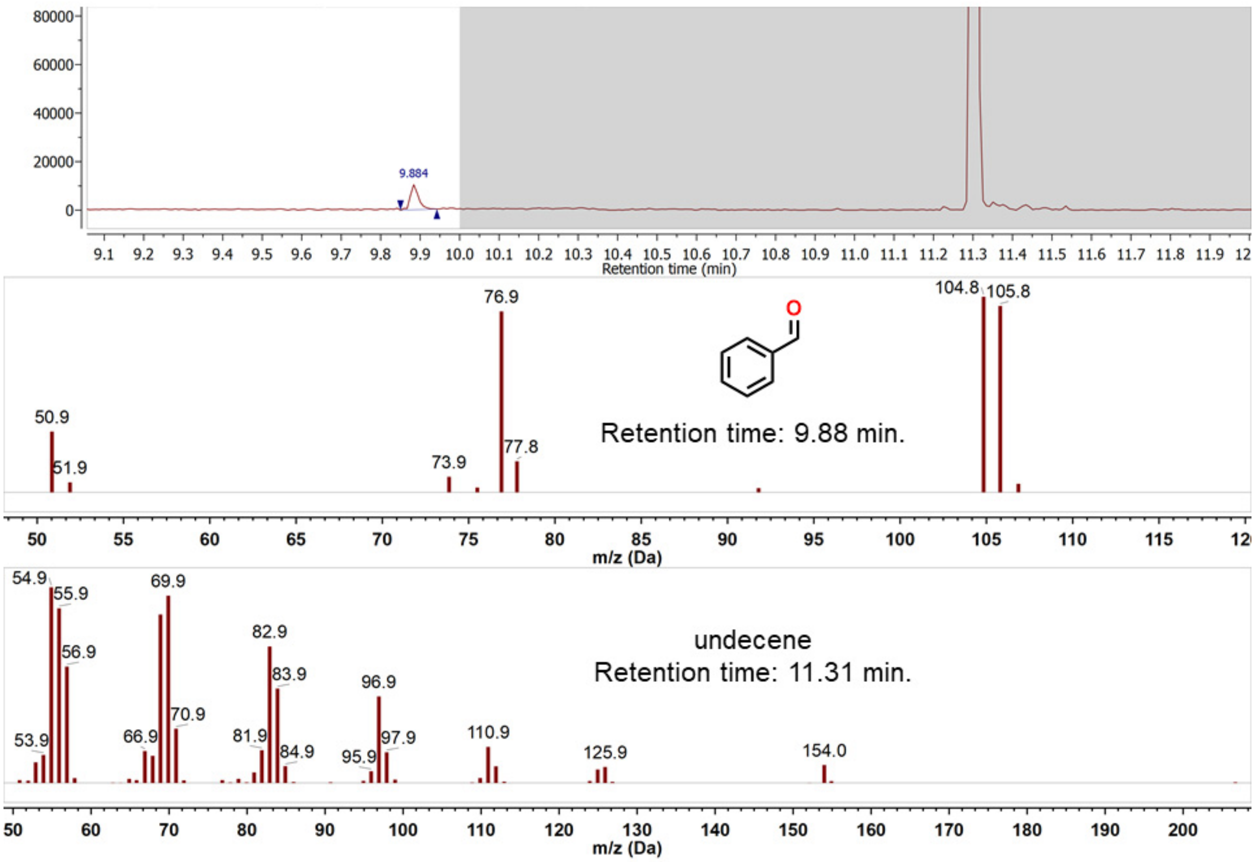

Figure S41. GC-MS of the resulting product mixture of the reaction of **1** with excess O_2 in toluene at room temperature. 1-undecene (10.4 equivalents) was also included as an internal standard for GC-MS.

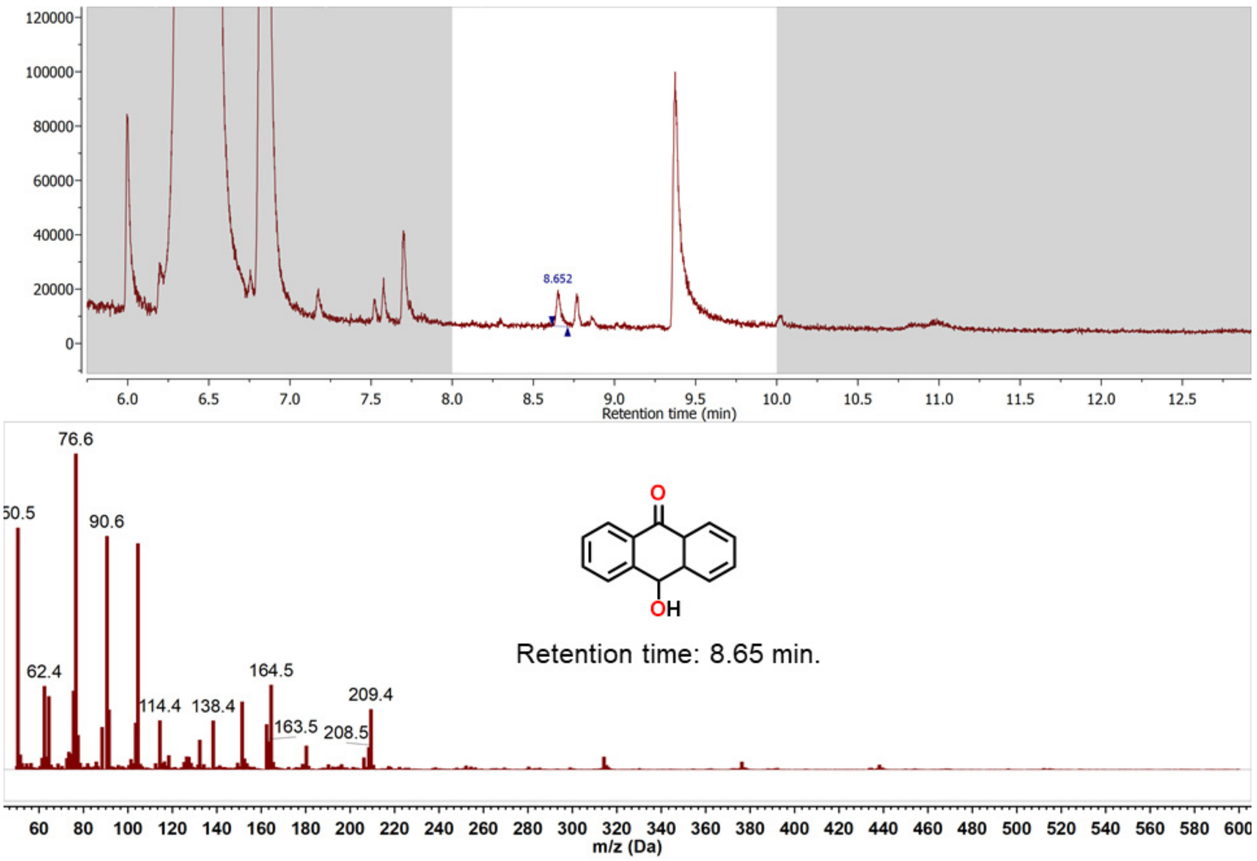

Figure S42. GC-MS of the reaction of **1** with $^{16}O_2$ or $^{18}O_2$ and DPBF to form 9-hydroxyanthracen-10(9H)-one.

Electrospray Ionization-Mass Spectrometry (ESI-MS

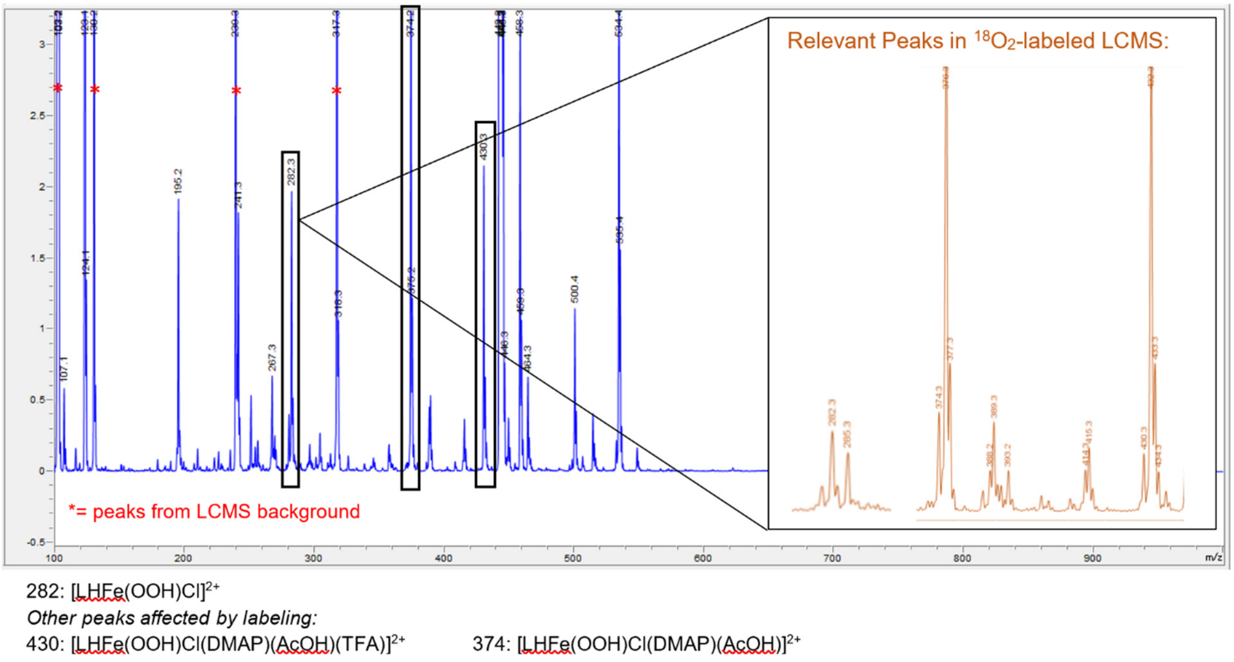

Figure S43. ESI-MS of the reaction of **1** with O_2 at −40 °C to form **3**. Isotope sensitive peaks and possible assignments are listed. Blue: $^{16}O_2$. Brown: $^{18}O_2$.

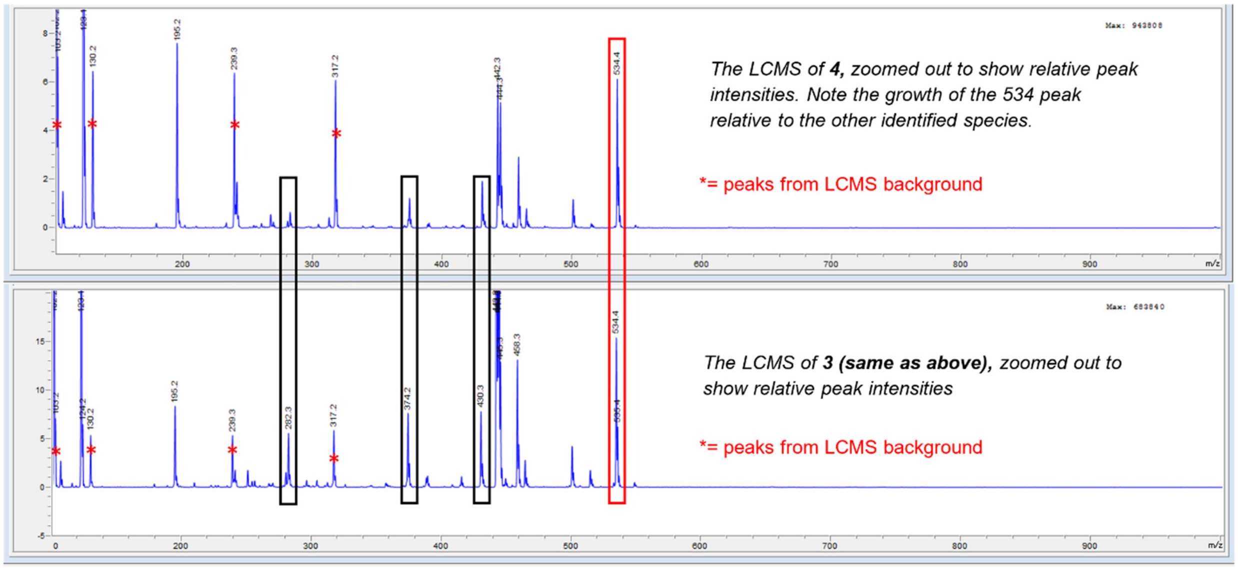

Figure S44. ESI-MS of the reaction of **1** with O_2 at −40 °C to form **4** (top). The peak at 534 m/z is potentially consistent with **4** where the DMAP auxiliary ligand has dissociated. Additionally, the features associated with **3** have decreased in intensity when comparing the ESI-MS of **3** (bottom) to **4** (top).

References

1 G. M. Sheldrick, *Acta Cryst*. **2015,** *C71*, 3-8.

2 O. V. Dolomanov, L. J. Bourhis, R. J. Gildea, A. K. Howard, H. Puschmann, *J. Appl. Cryst*. **2009**. *42*, 339.

3 G. M. Sheldrick. *Acta Cryst*. **2008***, A64*, 112-122.

4 (a) Neese, F. "The Orca Program System" *Wiley Interdisciplinary Reviews: Computational Molecular Science* **2012**, *2*, 73-78. (b) H - Kr: A. Schaefer, H. Horn and R. Ahlrichs, *J. Chem. Phys.* **1992**, *97*, 2571. (c) Rb - Xe: A. Schaefer, C. Huber and R. Ahlrichs, *J. Chem. Phys.* **1994** *100*, 5829. (d) F. Weigend, R. Ahlrichs, *Phys. Chem. Chem. Phys.* **2005**, 7, 3297.
